# Palm oil and dietary change: Application of an integrated macroeconomic, environmental, demographic, and health modelling framework for Thailand

**DOI:** 10.1016/j.foodpol.2018.12.003

**Published:** 2019-02

**Authors:** Henning Tarp Jensen, Marcus R. Keogh-Brown, Bhavani Shankar, Wichai Aekplakorn, Sanjay Basu, Soledad Cuevas, Alan D. Dangour, Shabbir H. Gheewala, Rosemary Green, Edward J.M. Joy, Nipa Rojroongwasinkul, Nalitra Thaiprasert, Richard D. Smith

**Affiliations:** a London School of Hygiene and Tropical Medicine, United Kingdom; b University of Copenhagen, Denmark; c SOAS University of London, United Kingdom; d Ramathibodi Hospital, Mahidol University, Thailand; e Stanford University, United States; f King Mongkut’s University of Technology Thonburi (KMUTT), Thailand; g Institute of Nutrition, Mahidol University, Thailand; h Chiang Mai University, Thailand; i University of Exeter, United Kingdom

## Abstract

Palm oil is a cooking oil and food ingredient in widespread use in the global food system. However, as a highly saturated fat, palm oil consumption has been associated with negative effects on cardiovascular health, while large scale oil palm production has been linked to deforestation. We construct an innovative fully integrated Macroeconomic-Environmental-Demographic-health (MED-health) model to undertake integrated health, environmental, and economic analyses of palm oil consumption and oil palm production in Thailand over the coming 20 years (2016–2035). In order to put a health and fiscal food policy perspective on policy priorities of future palm oil consumption growth, we model the implications of a 54% product-specific sales tax to achieve a halving of future energy intakes from palm cooking oil consumption. Total patient incidence and premature mortality from myocardial infarction and stroke decline by 0.03–0.16% and rural-urban equity in health and welfare improves in most regions. However, contrary to accepted wisdom, reduced oil palm production would not be environmentally beneficial in the Thailand case, since, once established, oil palms have favourable carbon sequestration characteristics compared to alternative uses of Thai cropland. The increased sales tax also provokes mixed economic impacts: While real GDP increases in a second-best Thai tax policy environment, relative consumption-to-investment price changes may reduce household welfare over extended periods unless accompanied by non-distortionary government compensation payments. Overall, our holistic approach demonstrates that product-specific fiscal food policy taxes may involve important trade-offs between nutrition, health, the economy, and the environment.

## Highlights

• Fully integrated Macroeconomic-Environmental-Demographic-health model.
• Palm oil sales tax creates dietary and health improvements.
• Palm oil sales tax causes temporary economic losses and adverse environment impacts.
• Policymakers should consider trade-offs when using fiscal food policy instruments.
• Fiscal food policy research should include both food and non-food policy indicators.

## 1 Introduction

Palm oil, a ubiquitous food ingredient, has brought economic benefit to Asia, but has also been linked with negative health and environmental outcomes. The rapid expansion of oil palm production in South-East Asia, with particularly high returns to land, labour, and capital in Indonesia and Malaysia, has been driven by growing demand for palm oil for food (Sheil et al., 2009). Palm oil has a high Saturated Fatty Acid (SFA) content relative to other cooking oils (Dubois et al., 2007), and the World Health Organization (WHO) reports ”convincing evidence” that consumption of palmitic acids contributes to increased risk of cardiovascular disease (CVD) (WHO, 2003). The health argument has, however, received little attention in the palm oil debate, which has mainly focused on adverse environmental and biodiversity consequences of oil palm cultivation (Koh and Wilcowe, 2008, Carlson et al., 2012). In order to study the important interconnections and trade-offs between health, environment and economic dimensions of palm oil production and consumption, we simulate a set of palm oil-related fiscal food policy scenarios, based on a newly constructed Macroeconomic-Environmental-Demographic-health (MED-health) simulation model with a CVD-focused health pathway, with Thailand as the test-case setting.

Thailand, the third-largest oil palm producer in the world, produces to meet domestic demand, and increased palm oil consumption is a stated policy priority. It therefore provides an appropriate test case for studying how palm oil-related fiscal food policies may affect economic and non-economic policy outcomes in a holistic way. The combination of high productivity and advantageous industrial properties has stimulated global demand for palm oil, and oil palm plantations currently account for 5% of the global harvested oil crop area and 40% of edible oil production (Petchseechoung, 2016). Over the past 30 years, Thai oil palm fruit production has mirrored international trends and exponentially increased to 12.4 mega-tonnes (Mt) in 2014 (FAO, 2017), and Thai palm oil production now amounts to 2.0 Mt or 1.2% of global supply (Petchseechoung, 2016). In contrast to Indonesia and Malaysia, where large scale plantations dominate, smallholders account for more than 85% of oil palm production in Thailand, and, while production efficiency and oil extraction rates remain low, palm oil prices have remained stable over recent years (Rewtarkulpaiboon, 2015). Increased production has relied on increased land use (FAO, 2017), but environmental degradation has so far been limited by small-holder conversion of degraded land and abandoned rice paddy fields in the south region (Silalertruksa and Gheewala, 2012a) where more than 87% of harvested areas are located (Rewtarkulpaiboon, 2015).

Oil palm is a strategic sector in Thailand. Previous development plans have focused on the production of edible oil and biofuel for domestic use (Dallinger, 2011). The current Thai Oil Palm and Palm Oil Industries Development Plan for 2015–2026 maintains sustainability, self-sufficiency, and food security as priorities (Rewtarkulpaiboon, 2015) and aims to expand palm plantation areas by 40%, increase productivity by 10%, improve oil extraction rates from current Thai rates of 14–17% to Malaysia’s 20%, and encourage oil consumption growth by 3% p.a. as well as increase the use of palm oil for biofuel (Petchseechoung, 2016).

At the same time, Thailand is in the middle of a dietary, nutrition and health transition. Since the 1980s, consumption of sugar and edible oils has increased rapidly at the expense of fruits and vegetables, and this has contributed to rapid growth in obesity and other diet-related diseases, with the prevalence of obesity quadrupling between 1991 and 2010 (Kelly et al., 2010). Thai household consumption of edible oils is dominated by palm oil, accounting for 75% of edible oil energy intake in the 2004–5 National Thai Food Consumption Survey (Kosulwat et al., 2006). Concurrent with the increase in oil palm production and palm oil consumption, crude death rates for ischaemic heart disease and stroke increased during 2000–2012, and these CVDs, now, constitute the two largest contributors to Thai mortality accounting for respectively 13.7% (68,800 deaths) and 10.3% (51,800 deaths) of all deaths in 2012 (WHO, 2015).

While available evidence on Thai palm oil consumption and CVD disease burden is suggestive, the significance of the clinical pathway, linking palm oil consumption to increased risk of CVD, is debated. Palm oil contains high ratios of SFA to Mono Unsaturated Fatty Acids (MUFA) and Poly Unsaturated Fatty Acids (PUFA). Substitution of SFA for MUFA and PUFA has been linked to increased serum cholesterol levels (Mensink et al., 2003) which, in turn, have been linked to increased incidence and mortality of Stroke and Myocardial Infarction (MI) (Basu et al., 2013). While a more recent ”Saturated Fat Controversy” literature has questioned the reduced form link between SFA intake and clinical health outcomes (Ravnskov et al., 2016, Beluri, 2016), other compelling evidence has forcefully argued that ”findings support current dietary recommendations to replace saturated fat … with unsaturated fats” (Wang et al., 2016a, Wang et al., 2016b).

We adopt the above-mentioned clinical health pathway, and proceed to quantify nutritional, environmental, demographic and health sub-modules in order to construct a fully integrated MED-health model with sector-specific detail for palm oil and other substitute edible oils. Specifically, we calibrate a recursive-dynamic Computable General Equilibrium (CGE) economic model for Thailand for the years 2016–2035, and proceed to integrate a nutrition-related serum cholesterol biomarker pathway, which is extended to measure CVD-related clinical health outcomes and feedback effects on labour market participation and health system costs, and a Land Use Change (LUC) environmental module. Our health pathway has previously been investigated in a partial equilibrium study of palm oil taxation and health outcomes in India (Basu et al., 2013), but it has not been studied within a macroeconomic general equilibrium framework or within a holistic MED-health model.

In order to put the stated policy priorities of future palm oil consumption growth into perspective, our policy scenario imposes a product-specific sales tax to achieve a halving of future energy intakes from palm cooking oil consumption. The fiscal food policy literature supports that taxation and subsidy interventions influence dietary behaviours (Niebylski et al., 2015), but evidence has, so far, been limited to nutritional policy outcomes. We add to this literature by applying our general equilibrium and holistic MED-health model to compute consistent and holistic sets of nutrition, health, economic, demographic, and environmental impacts. Moreover, since nutrient taxes have previously been cancelled due to inelastic demand responses and bureaucratic burdens (Stafford, 2012), we focus on employing a sales tax instrument for moving towards a healthier national diet in Thailand.

## 2 Background literature

Socio-economic quantitative studies of oil palm are few. The literature consists of household-level econometric studies which highlight the benefits of oil palm cultivation and contract farming for smallholder producers in Indonesia (Cahyadi and Waibel, 2013, Euler et al., 2017), and a few macroeconomic simulation studies, including a global CGE study which demonstrates the potential benefits of acceleration in oil palm yield growth in Indonesia and Malaysia (Villoria et al., 2013) and a single country CGE study which argues that increased export taxes and higher world market prices for palm oil has been beneficial for household welfare in Indonesia (Ikhsan et al., 2016).

Economy-wide simulation studies of health policy scenarios have previously been applied to assess either disease-specific epidemics, or large-scale environmental problems with multiple-disease impacts (Ciscar et al., 2010, Jensen et al., 2013). However, to our knowledge, no previous CGE model studies have fully integrated illness-specific health pathways with feedback effects on labour markets and health system costs. The only published Thai-specific CGE study of health impacts analysed a set of greenhouse gas (GHG) mitigation scenarios, with a focus on carbon taxation and ancillary benefits of reduced local air pollution, but, while it did attempt to capture feedback effects on labour markets and health system costs, these were implemented recursively and not endogenously (Li, 2002). Importantly, this is, to our knowledge, the first time a structural health pathway has been integrated within a framework which allows for consistent measurement of health, environment, and economic policy outcomes.

The broader quantitative literature on pathways between income, nutrition, health, and labour markets is diverse. Most econometric studies of income and nutrition focus on child nutrition (e.g. Smith and Haddad, 2002) while most econometric studies of income and health focus on reduced form impacts of income on well-being indicators, but without accounting for underlying nutritional and health system pathways (e.g. Deaton and Arora, 2009). The empirical literature on returns to improved nutrition generally distinguishes between studies of (1) long-term impacts of childhood nutrition, and (2) contemporaneous impacts of nutrition on adult productivity, where the latter is mainly focused on the efficiency wage theory and how wages respond to nutritional intakes of workers (Alderman and Sahn, 2016). Little evidence is available from macroeconomic simulation models. Soft integration of CGE and epidemiological models, i.e. application of satellite epidemiological models to provide inputs to CGE models, has been pursued for infectious diseases (see e.g. Kambou et al., 1992, Jefferis et al., 2008, Thurlow et al., 2009) and a small number of nutritional CGE studies have relied on fixed caloric food coefficients to measure nutritional outcomes, but without fully integrated clinical health feedbacks to the economy (Minot, 1998, Pauw and Thurlow, 2011). Furthermore, existing nutritional CGE studies tend to measure caloric intakes and nutrition deficits without measuring intakes of specific nutrients and excess nutritional intakes (e.g. leading to high cholesterol and obesity).

The ambitious and ongoing SUSTRANS project aims for soft model integration of a comprehensive economic, environmental, nutritional, and clinical health model framework for the EU (Rutten et al., in press). While Rutten et al. aim to construct a multi-purpose tool which is broad in scope, we aim to take their work one step further and fully integrate a concrete nutrient-focused health pathway within a macroeconomic and environmental CGE model. Specifically, we construct a fully integrated MED-health model for Thailand, with a nutrient-related serum cholesterol biomarker health pathway: (1) endogenous calculation of food demand and changes in fatty acid exposures, and their subsequent impacts on (2) regional population distributions of serum cholesterol biomarker levels, (3) regional clinical outcomes in terms of CVD-related incidence and excess mortality rates of stroke and MI, and (4) economic feedback effects on labour market outcomes and health system costs. Since we analyse nutritional impacts over a 20 year period (2016–35) and since generational impacts tend to have a longer time perspective, we do not include long-term child nutrition pathways in our model. Instead, we focus on contemporaneous labour force impacts of changes in excess mortality rates and absenteeism.

Our MED-health model also includes an agro-environmental module to measure LUC-related GHG emissions of CO_2_-equivalents (CO_2_-eq). A few CGE studies have previously analysed environmental policy impacts in Thailand, including single-country studies of GHG and local air pollution from Thai trade liberalization and carbon tax policy (Li, 2002, Li, 2005), and a global CGE study of GHG emissions from forest conversion associated with biofuel sector expansion (Timilsina and Mevel, 2013). While excluding environmental indicators, a recent CGE study of Thai biofuel production also provided suggestive evidence on how future biofuel expansion may lead to increased demand for palm oil and land use for oil palm production (Wianwiwat and Asafu-Adjaye, 2013). Other Thai-specific environmental studies of oil palm and palm oil production include more detailed LUC studies of oil palm production (Saswattecha et al., 2016), as well as partial life-cycle (Saswattecha et al., 2015) and full life-cycle (Silalertruksa et al., 2017) LUC- and process-oriented studies. While the latter studies provide a variety of environmental and ecological indicators which we do not include in our study, e.g. water use and biodiversity, they all share our agro-environmental focus on GHG emissions and carbon sequestration.

## 3 Model framework and data sources

**Fig. 1:**
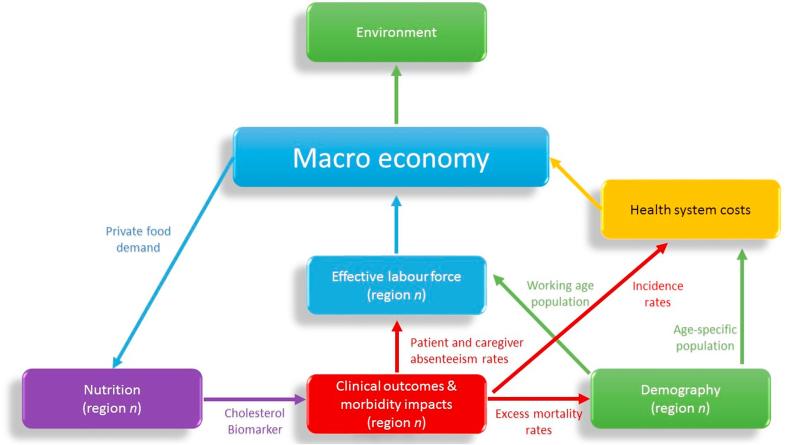
MED-health model framework and feedback effects between the macroeconomy and regional sub-models.

Our MED-health model framework for cholesterol-related CVDs is illustrated in Fig. 1. The central feature is that economic incentives from the macroeconomic sub-model affect regional nutritional intakes in the nutrition sub-module (through food consumption demand exposure) and, via serum cholesterol biomarker build-up, clinical health outcomes in the clinical health outcome sub-module (deriving distributions of illness-specific incidence and mortality rates from distributions of nutrition-related biomarker values), and this affects regional effective labour force participation rates (through working-age patient and caregiver time losses) and regional population distributions (through patient mortality). Morbidity and demographic outcomes, subsequently, interact to produce labour force and health system cost impacts which feed back into the macroeconomic model. The endogenous feedback effects are specified, separately, for nine regional Thai household types (Bangkok and rural/urban splits of south, central, north, and northeast regions), allowing intervention strategies to have region-specific impacts in the south, where oil palm is a cornerstone of rural livelihoods and regional development, and allowing simulated disease burdens to reflect regional variations in dietary exposure. An environmental sub-module is used to measure LUC-related GHG emissions.

We calibrated our CGE model from a 2007 Thailand SAM data set (NESDB, 2015). The original 260 commodities were aggregated into 49 commodities with the aim to (1) reduce computational and analytical complexity, but also to (2) maintain all major intermediate demand pathways, and (3) maintain key commodities (primary oil palm, processed edible oils sectors including palm oil, coconut oil, and other edible oils, and bio-fuel sectors including methanol, ethanol, Gasoline 91/95, Gasohol E10/91, Diesel, Diesel B2/B5). Furthermore, we disaggregated the single household account into 9 regional households based on prior information obtained from the 2011 Household Socio-Economic Survey (NSO, 2014). The resulting unbalanced SAM matrix was balanced using standard cross minimum entropy techniques (Golan et al., 1994, Robinson et al., 2001).

The CGE model was further extended to include an Almost Ideal Demand System (AIDS) specification of private demand for each of our nine regional households, based on price and income elasticities derived from the literature (Suebpongsakorn, 2008). Due to a lack of detail about primary agricultural and edible oil sectors, the original Suebpongsakorn data set was complemented by uncompensated cross-price elasticities for primary agricultural commodities from a recently published Thai-specific food demand system (Lippe and Isvilanonda, 2010), and non-Thai edible oil cross-price elasticities from the literature (Yen and Chern, 1992, Kim and Chern, 1999). Neither the original nor expanded cross-price elasticity matrices satisfied basic regularity conditions (symmetry and adding-up). In addition, the original Suebpongsakorn data set did not match the 49 sector classification underlying our CGE model, and the demand share patterns, underlying the Suebpongsakorn data set, did not match either the overall or the disaggregated household demand patterns for our nine regional households. In order to properly parameterize the AIDS demand systems for each of our nine regional households, we therefore constructed a sector mapping and employed standard uncompensated price and income elasticity formulae (Green and Alston, 1990, Green and Alston, 1991) to produce prior unbalanced parameter values for our region-specific AIDS-demand systems, based on household-specific relative demand shares. A Bayesian minimum cross-entropy analysis, specified with a full set of regularity constraints, was subsequently used to derive a full set of posterior parameter values including a 49 × 49 cross-price elasticity matrix (satisfying all regularity conditions) and a 49 income elasticity vector (satisfying adding-up constraints) for each of our nine regional households.

In order to measure nutritional changes from private food demand exposure, we constructed separate nutrition modules for each of our nine regional households. Nutritional coefficients were derived from the 2004–5 National Thai Food Consumption Survey (TFCS) (Kosulwat et al., 2006, Jitnarin et al., 2010) and the 2011 Household Socio-Economic Survey (HSES) (NSO, 2014). The former 2004–5 TFCS survey contained ready-calculated intakes of nutrients including fatty acids, while nutrient contents of food consumption from the 2011 HSES survey were based on nutritional values derived from the Thai food composition table of the SMILING database (SMILING, 2016) and the USDA food composition database (USDA, 2014). Food categories were mapped to the 49 commodities of the SAM database, and separate household- and commodity-specific coefficients were established for total energy intake, and energy intakes from individual nutrients including Saturated Fatty Acids (SFA), Monounsaturated Fatty Acids (MUFA), and Polyunsaturated Fatty Acids (PUFA).

It has previously been shown how changes in the composition of fatty acid energy intakes affect changes in consumers’ serum cholesterol levels (Mensink et al., 2003). However, since our AIDS demand systems are based on standard utility maximization (without iso-caloric constraints), our dynamically-recursive model produces changes in both levels and compositions of energy intakes. Due to a lack of evidence about increased energy intake levels, we follow Basu et al. (2013) and focus on compositional effects. Based on parameter estimates of Mensink et al. (Table 1), the household-specific nutritional coefficients allowed for establishing a nutrition module which computes household-specific average energy intake shares from SFA, MUFA and PUFA, and, subsequently, uses them to calculate average and stratified household-specific changes in Total:HDL serum cholesterol ratio (ΔC) levels from Eq. (1):

(1) $$\Delta {\text{C}}_{\text{h},\text{t}}=\alpha^{\text{SFA}}\;\Delta {\text{SFA}}_{\text{h},\text{t}}+\alpha^{\text{MUFA}}\;\Delta {\text{MUFA}}_{\text{h},\text{t}}+\alpha^{\text{PUFA}}\;\Delta {\text{PUFA}}_{\text{h},\text{t}}\;\text{h}\;\varepsilon \;\text{H},\;\text{t}\;\varepsilon \;\text{T}$$

**Table 1:** Parameters linking changes in Total:HDL serum cholesterol ratio to changes in SFA, MUFA and PUFA energy intake shares.

	α^SFA^	α^MUFA^	α^PUFA^
Central parameter estimates	0.003	−0.026	−0.032

for each of our nine household types (H) and 20 time periods (T). While our simulation framework is set up to model average serum cholesterol biomarker changes at the regional household level, the availability of individual-specific biomarker level data allowed us to compute intra-household biomarker distributions and thereby refine the calculation of clinical health outcomes. Specifically, we shift intra-household distributions of biomarker levels by the mean according to Eq. (2):

(2) $${\text{C}}_{\text{h},\text{s},\text{t}}^{\text{strata}}={\text{C}}_{\text{h},\text{s},\text{t - 1}}^{\text{strata}}+\Delta {\text{C}}_{\text{h},\text{t}}\;\text{h}\;\varepsilon \;\text{H},\;\text{s}\;\varepsilon \;\text{STRATA},\;\text{t}\;\varepsilon \;\text{T}$$

A set of 10 support points (STRATA) were specified for each intra-household biomarker distribution, and this was matched by the construction of 11 sets of lookup-tables for clinical health outcomes covering 10 intervals stratified to include the 10 initial support points as their mid-points. Frequency distributions for the 10 intervals were based on individual-level biomarker data from the 2008–2009 Thailand National Health and Examination THES Survey (NHESO, 2009, Aekplakorn et al., 2011).

Simulated age-, gender-, and rural/urban location-specific lookup tables for clinical outcomes were derived from random sampling of 10,000 simulated individuals from normal distributions of biomarker data derived from the 2008–2009 THES Survey (NHESO, 2009) (see online appendix A.1 for details). Stratification covered 13 five-year age groups including 11 working age groups (15–64 years), and two retirement age groups (‘65–69 years’ and ‘70+ years’). The 11 sets of simulated lookup tables were established, to match the end-points of the 10 equidistant sub-intervals of the Total:HDL cholesterol ratio biomarker interval [2.0; 7.0]. For current purposes, a set of age-, gender-, and rural/urban location-specific 10th degree polynomials were fitted for calculation of incidence and excess mortality rates for MI and stroke.

The demographic module was designed to mirror the population stratification of the health and nutrition modules. A set of 2010–35 Thai regional population projections was obtained from the National Economic and Social Development Board (NESDB, 2013a, NESDB, 2013b), and the age-, gender, and rural/urban location-specific projections were subsequently employed to calibrate our demographic model, encompassing a compact set of equations for calculating Births, Deaths, Migration, and Population levels (see online appendix B for details). Due to lack of information about underlying parameter assumptions, age- and gender-specific demographic parameters were obtained from the 2015 Revision of UN population projections (UN, 2015).

The clinical health outcome module, combined with the demographic module, produces numbers of MI and stroke incident cases and premature deaths. Patient incidence numbers are subsequently employed to calculate Years Lost due to Disability (YLD) patient morbidity impacts, informal caregiver time losses, and formal hospital costs, while the numbers of age-specific premature deaths are used to calculate demographic population impacts and patient-specific mortality-related changes to the workforce. Computation of YLD morbidity impacts for MI and stroke are based on YLD weights from the literature (WHO, 2013) (see online appendix A.2 for details), while calculations of caregiver time losses for stroke, including leisure and worktime losses, are based on Thai-specific average time loss estimates (Riewpaiboon et al., 2009).^1^ Thai-specific hospital unit costs were also derived from the literature, including MI-related hospital unit costs (Anukoolsawat, Sritara and Teerawattananon, 2006) and stroke-related hospital unit costs (Khiaocharoen, Pannarunothai and Zungsontiporn, 2012), while workforce impacts were corrected for Thai-specific workforce participation rates (NSO, 2008).

Finally, the environment module focuses on croplands and LUC-related GHG emissions. Thailand was among the first signatory countries to the UN Framework Convention on Climate Change in 1992 and a signatory to the Paris Agreement, and, although a non-Annex I country, Thailand has chosen to pursue an ambitious low carbon economy strategy (OEPP, 2010). There is a debate over the relative importance of direct LUC (dLUC) vs. indirect LUC (iLUC). Recent Thai-specific evidence from the key Tapi river basin in the south region indicates that, while dLUC dominated during 2000–2009, iLUC has increased, more recently, due to expansion of oil palm in rubber cultivation areas (Saswattecha et al., 2016). Hence, oil palm expansion may, indirectly, have led to relocation of rubber plantations into previously forested areas. Nonetheless, other evidence suggests that environmental degradation has so far been limited by smallholder conversion of marginal lands including former paddy fields, abandoned fruit orchards, and wasteland (Silalertruksa and Gheewala, 2012a). Since the potential for marginal land conversion has not yet been exhausted, moderate future Thai oil palm expansion should be able to proceed without requiring iLUC-related deforestation.

While recognizing the potential danger of indirect impacts on forested areas, we specify our environmental module to focus, narrowly, on measurement of dLUC impacts on carbon sequestration, based on Thai-specific LUC-coefficients (Silalertruksa and Gheewala, 2012b). The database on LUC-related GHG emissions covers 20 primary agricultural crops, and a mapping between these crops and the 7 primary agricultural crops in our CGE model allowed us to derive a 7 × 7 matrix of LUC-related GHG emission coefficients which was used to calibrate the environmental module of our integrated model framework.

The following section presents results of our policy scenario: Introduction of a product-specific sales tax to achieve a 50% reduction in per capita energy intakes from palm cooking oil consumption. While previous simulation studies have indicated that nutrient taxes could be more effective than product-specific taxes in achieving nutrition targets (Harding and Lovenheim, 2017), existing fat taxes have proved to be fairly inelastic (Chouinard et al., 2007, Allais et al., 2010), and the Danish attempt at introducing a nutrient tax on foods, high in saturated fats, was repealed after only one year, due to bureaucracy, spiralling administrative costs and the threat of job losses (Stafford, 2012). There is no Thai-specific evidence on price elasticities of palm cooking oil, but non-Thai evidence suggests that demand for palm oil is fairly elastic compared to other types of edible oils (Kim and Chern, 1999). We therefore chose to employ a product-specific sales tax instrument to achieve our nutrition target.

Our counterfactual 2016–35 growth path is based on historical real (3.9% p.a.) and nominal (6.2% p.a.) Thai GDP growth rates over the past 15 years (1998–2014) and on a balanced macro closure with a fixed government consumption-to-absorption ratio. We employ a neo-classical model closure with price clearing of all goods and factor markets, and with the maintained assumption of full employment in factor markets. The supply of capital is governed by a standard capital accumulation specification, while the expansion of skilled and unskilled labour supplies are derived from the demographic working age population (15–64 years) based on a fixed skilled-to-unskilled labour ratio (NSO, 2008) and corrected for fixed workforce participation rates (NSO, 2008). For the policy simulation, we impose an iso-government budget constraint whereby the government consumption budget is fixed at the counterfactual growth path to ensure that the growth path of government expenses (in terms of the CPI price numeraire) is not affected by increased sales tax revenues. Increased revenues are returned to households through uniform additive reductions in direct tax rates, which do not affect incentives in our model. In terms of free market response, the Thai palm oil market has been heavily regulated since the 1980s. The Thai government has been managing palm oil imports since 1982, and they are currently running a two-tiered tariff-system under WTO with a 20% tariff for imports below 4860 tonnes, and a prohibitive 143% tariff above this level (Petchseechoung, 2016). In addition, the Thai government is operating price targets, including (occasional changes to) price ceilings on palm cooking oil and price floors on oil palm fruit, in order to simultaneously support low-income consumers and smallholder producers. Nonetheless, the conflicting targets have led to occasional imports, and domestic and world market prices of crude palm oil have been correlated over the past decade (Petchseechoung, 2016). In this paper, we assume that long-term world market price changes will feed-through to domestic prices, and, for the policy simulation, we specifically assume that the introduction of our sales tax instrument is accompanied by a relaxation of price controls.

## 4 Policy simulations and results

We simulate one scenario, a 50% reduction in average per capita energy intakes from palm cooking oil consumption. A 54% product-specific sales tax is required to meet our target over the long run (2035), and the resulting economic, nutritional, health, demographic, and environmental impacts are presented in Table 2. The table includes a results decomposition which isolates economic (and non-economic) welfare consequences of our health pathway from our product-specific sales tax pathway. Since palm oil and other edible oils turn out to be the main drivers of changes in fatty acid consumption and composition, we believe that the health pathway impacts may provide clues to impacts, not only of our current sales tax instrument, but of more targeted interventions which could potentially avoid broader substitution patterns and welfare impacts.

**Table 2:** 50% reduction in palm oil consumption: Long-run and cumulative impact indicators for 2016–35.

Economic Indicators			Nutrition, biomarker, health indicators			Demographic, environment indicators		
***Real GDP & sales tax (cumulative and long run indicators)***			***Nutrition (long run indicators)***			***Demographic (cumulative indicators)***		

	mn USD	% of GDP		%-points	% of share		pers-yrs	% of total
Δreal GDP (cum.)	25,145	0.227%	ΔSFA energy intake share (2035)	−0.322%	−3.58%	Δpopulation	13,621	0.0010%
sales tax pathway (cum.)	25,050	0.226%	ΔMUFA energy intake share (2035)	−0.164%	−2.29%	Bangkok^2^	886	0.0005%
health pathway (cum.)	95	0.001%	ΔPUFA energy intake share (2035)	0.298%	5.80%	Central region (exc Bangkok)^1^	7387	0.0018%
	mn USD	% of GDP	***Biomarker (cumulative indicator)***			North region^2^	2544	0.0011%
Δreal GDP (cum.)	25,145	0.227%		cum. chg.	% of total	Northeast region^2^	2721	0.0007%
Private Consumption (cum.)	−9882	−0.177%	ΔTotal-to-HDL cholesterol ratio	−0.101	−2.16%	South region^2^	83	0.0000%
Government Consumption (cum.)	8633	0.854%	***Health (cumulative indicators)***			Urban^2^	5019	0.0007%
Investment (cum.)	26,394	0.558%		cases	% of total	Rural^2^	8602	0.0015%
Exports (cum.)	35,857	0.468%	ΔPatient Incident Cases	−3570	−0.095%		pers-yrs	% of total
Imports (cum.)	35,857	0.455%	Myocardial infarction	−2704	−0.160%	Δworkforce	4450	0.0007%
	%-points		Stroke	−866	−0.042%	Urban^2^	1621	0.0004%
Δsales tax (2035)	53.5%		ΔPatient premature deaths	−1861	−0.098%	Rural^2^	2829	0.0010%
Δinvestment price index (2035)	−0.84%		Myocardial infarction	−1560	−0.152%	***Environment (cumulative indicator)***		
Δreal exchange rate (2035)	−0.85%		Stroke	−301	−0.034%		Mt CO_2_-eq	
***Real Consumption (cumulative indicators)***				pers-yrs	% of total	GHG emissions	7.52	
	mn USD	% of rHC	ΔPatient Disease Burden (YLD)	−777	−0.043%			
Δreal Household Consumption	−9882	−0.177%	Myocardial infarction	−4	−0.160%			
sales tax pathway	−9930	−0.178%	Stroke	−773	−0.043%			
Bangkok^1^	−362	−0.041%	ΔPatient Worktime Loss	−362	−0.060%			
Central region (exc Bangkok)^1^	−3336	−0.249%	Myocardial infarction	−2	−0.182%			
North region^1^	−935	−0.139%	Stroke	−360	−0.060%			
Northeast region^1^	−982	−0.089%	ΔCaregiver Time Loss (stroke)	−1587	−0.042%			
South region^1^	−4315	−0.563%	Work time	−643	−0.043%			
health pathway	48	0.0009%	Leisure time	−944	−0.041%			
Bangkok^1^	8	0.0008%		mn USD	% of GDP			
Central & East region^1^	19	0.0013%	ΔHealth Expenses (Formal hospital)	39	0.0003%			
North region^1^	7	0.0009%	Myocardial infarction	34	0.0003%			
Northeast region^1^	9	0.0008%	Stroke	5	0.0000%			
South region^1^	5	0.0007%						
								

Note: Own calculations. ^1^ Regional consumption %-impacts calculated as share of projected regional totals ^2^ Regional population %-impacts calculated as share of projected regional totals.

### 4.1 Economic impacts from sales tax pathway and feasibility of policy instrument

The sales tax pathway has dichotomous macroeconomic impacts, raising Thai real GDP by USD 25.0bn and lowering real household consumption by USD 9.9bn over the coming 20 years (Table 2). Our government budget-neutral model closure keeps household budgets stable, but the tax-induced increase in relative food vs. investment prices lowers real household consumption and increases real investment and long run real GDP. Thus, our palm oil sales tax (without compensating government transfers) leads to intergenerational welfare redistribution in favour of future generations. The relatively steep increase in capital accumulation and real GDP (not shown) indicates that the benefits accrued by future generations, beyond our 20 year time horizon, is likely to exceed what current generations forgo in terms of real welfare. While the break-even point is beyond our time horizon, our set of iso-household consumption sensitivity analyses, which isolates efficiency gains on the producer side, confirm that government compensation policies can avert household welfare losses and lead to real GDP gains. Nonetheless, with unchanged government budgets, palm oil sales taxes are likely to lead to net private consumption welfare losses both in a static context and over prolonged periods of time (see sensitivity analyses below and online appendix C.5).

**Fig. 2:**
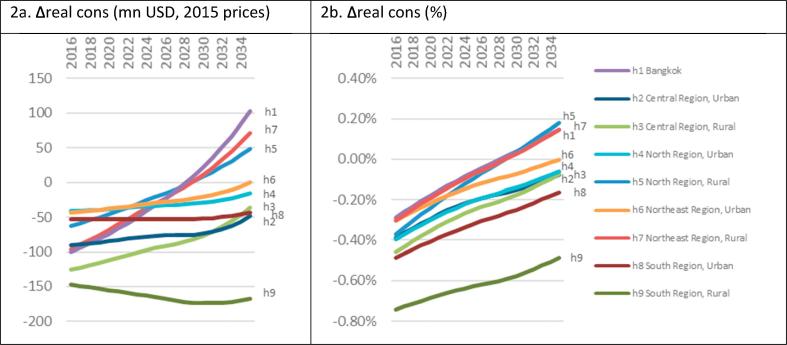
Real household consumption impacts of −50% palm oil consumption. Note: own calculations.

The sales tax pathway has a particularly strong adverse consumption welfare impact in the south region amounting to −0.72% over the coming 20 years (Table 2). However, dynamic regional consumption impacts (Fig. 2) show that other regions will suffer less. All regions experience initial welfare losses (Fig. 2a), but increased capital accumulation reduce relative welfare losses over time and increase welfare for some regions in the long run. Bangkok experiences the largest long run welfare gain in absolute terms, but substitution in aggregate food demand, away from palm oil and oil palm produced in the south, means that remaining non-south rural areas also benefit and bear smaller burdens relative to their counterpart urban areas (Fig. 2b). Importantly, Thai food demand turns out to be responsive to our fiscal instrument. The (transitional) welfare losses are therefore complemented by improvements in dietary composition.

### 4.2 Economic impacts from the health pathway

Overall, the health pathway raises Thai real GDP and real household consumption by respectively USD 95mn and USD 48mn over the next 20 years. A shift in the balance of energy intake shares between SFA (−0.32%-points), MUFA (−0.16%-points) and PUFA (+0.30%-points), leads to a 2.2% reduction in the Total:HDL serum cholesterol ratio, and up to 0.2% reductions in CVD-related clinical outcomes and caregiver time losses (Table 2). The Thai population and labour force expands by 13,621 and 4450 person-years, respectively, and, together with saved medical expenses, this drives real GDP impacts of the health pathway. Consumption welfare gains benefit all Thai regions (0.0007–0.0013%) (Table 2), but rural areas benefit more (0.0010%) compared to urban areas (0.0007%) (not shown). Due to the unequal distribution of income and health services in favour of urban areas, the health pathway is therefore likely to be beneficial, progressive and pro-poor in economic terms.

### 4.3 Nutrition, biomarker, health, and population impacts from the health pathway

**Fig. 3:**
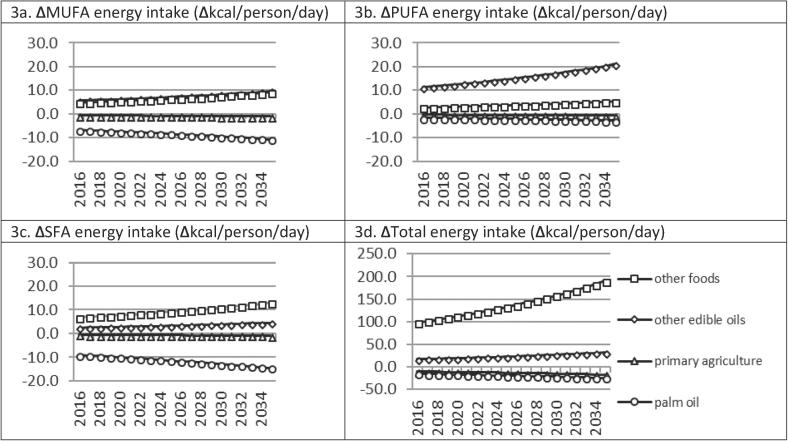
Commodity- and nutrient-specific energy intake impacts of −50% palm oil consumption. Note: own calculations.

**Fig. 4:**
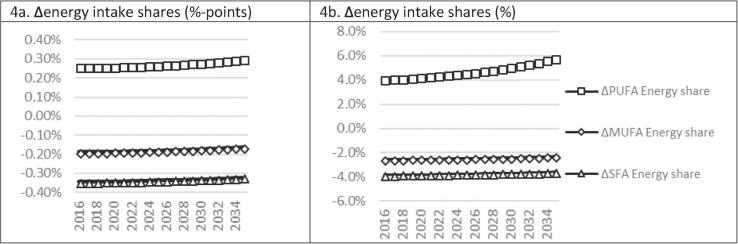
SFA, MUFA and PUFA nutrient-specific energy intake share impacts of −50% palm oil consumption. Note: own calculations.

Health pathway impacts are driven by changes in nutritional exposure (Fig. 3). Reduced palm oil consumption leads, on average, to complementary reductions in primary agricultural energy intakes and substitution towards other edible oils and other processed foods (Fig. 3d). The halving of palm oil consumption, by itself, lowers long run energy intakes from SFA (−14.6 kcal/day), MUFA (−10.6 kcal/day), and PUFA (−2.8 kcal/day), but substitution in Thai diets leads to positive net increases of 2.1 kcal/day, 7.0 kcal/day and 22.8 kcal/day respectively (Fig. 3a–c). Overall, average energy intake shares decline over the long run for SFA (−3.6%) and MUFA (−2.3%), and increase for PUFA (5.8%) (Table 2; Fig. 4b).

The combination of reduced SFA intake shares and increased PUFA intake shares causes the lowering of the average cumulative Total:HDL serum cholesterol ratio by 2.2% in 2035 (Table 2). The strong substitution towards PUFA-dense food groups is the main driving force in reducing nutritional exposure. Patient mortality and incident cases decline by 0.15–0.16% for MI and by 0.03–0.04% for stroke, and caregiver work and leisure time losses (for stroke patients) are reduced by 0.04% over the next 20 years. For MI and stroke, respectively, incidence declines by 2704 and 866 cases, YLD declines by 4 and 777 life years, and the cumulative number of premature deaths declines by 1560 and 301 over the next 20 years. While notable, our results suggest that halving of palm cooking oil consumption would have a relatively small impact on health. It would therefore have to form part of a broader dietary and nutritional strategy (perhaps combined with non-behavioural non-lifestyle interventions) to achieve more important improvements in clinical health outcomes.

**Fig. 5:**
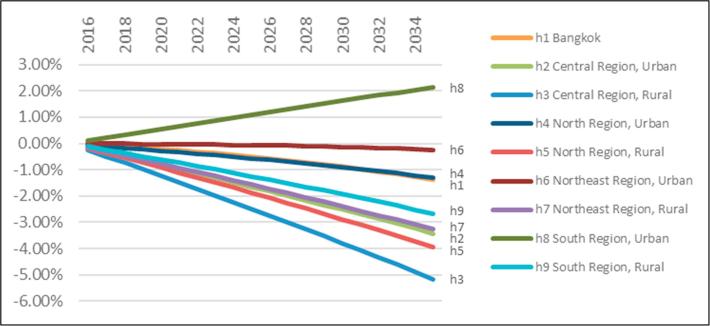
Total:HDL serum cholesterol ratio impacts of −50% palm oil consumption (%). Note: own calculations.

Region-specific nutritional composition impacts (not shown) vary widely: long run SFA energy intake share impacts range from −6% to −1%, while MUFA and PUFA share impacts range from −5% to 0% and from +3% to +7%, respectively. This causes long run cholesterol biomarker impacts to range from −5.2% in the rural central region, to +2.1% in the urban south region (Fig. 5). However, except for urban southern households, where demand substitution leads to adverse nutritional outcomes, Thai consumers, generally, respond to increased palm oil prices through fairly strong healthy demand expansion of PUFA-dense food groups.

**Fig. 6:**
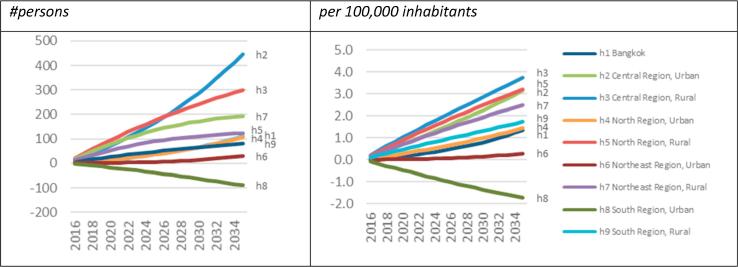
Regional population impacts of −50% palm oil consumption. Note: own calculations.

The switch to healthy eating saves, on average, 1.0 persons per 100,000 inhabitants (13,621 person years) over the next 20 years (Table 2), or 1.9 persons per 100,000 inhabitants (1300 persons) in 2035 (not shown). Aggregate rural and urban long run impacts vary between 2.9 and 1.3 persons per 100,000 inhabitants (not shown), while region-specific long run impacts vary between 1.7 and 3.7 persons per 100,000 inhabitants in rural regions and 0.3–3.2 persons per 100,000 inhabitants in urban regions excluding urban south where the population declines by 1.7 persons per 100,000 inhabitants (Fig. 6). The notable differences in long-run regional population impacts, favouring rural over urban areas, reflect varying food budget shares of palm cooking oil in Bangkok (1.6%), other urban areas (1.3–2.0%), and rural areas (2.0–2.8%), and they underline that health pathway impacts are likely to be progressive and pro-poor in terms of both health, demographic, and economic outcomes.

### 4.4 Environmental impacts

Finally, the palm oil sales tax and related reductions in oil palm production reduces land use for oil palm cultivation, significantly. The resulting land reallocation, away from oil palm towards other agricultural crops with less beneficial carbon sequestration characteristics, increases GHG emissions by a cumulative 7.5 Mt CO_2_-eq over the next 20 years (Table 2). The adverse environmental impact, overwhelmingly, stems from reduced carbon sequestration among smallholder producers in the south province, where oil palm production is mainly conducted on marginal lands with minimal alternative carbon sequestration potential.

### 4.5 Sensitivity analyses

We perform four sets of sensitivity analyses: (1) a nutrition sensitivity analysis to analyse how the parameterization of the empirical cholesterol biomarker specification (Eq. (1)) affects the outcomes of the health pathway, (2) two labour market sensitivity analyses to analyse the importance of labour market structures for the general transmission of health, economic and environmental outcomes, (3) three sets of analyses of palm oil-related price and income demand elasticities, and (4) a set of iso-household welfare sensitivity analyses which isolates efficiency losses on the producer side and allows us to analyse compensating government transfers and economic efficiency of the sales tax pathway. Scenarios and detailed analyses of sensitivity results are presented in online appendix C.

Our nutrition sensitivity analysis (online appendix C.1) varies cholesterol biomarker parameters between lower and upper confidence bounds (Mensink et al., 2003). Four scenarios are distinguished: Two extreme scenarios 1 and 2 where the three parameters are uniformly increased and reduced to their upper/lower bounds, a ‘uniform parameter’ scenario 3 (SFA parameter at upper bound, MUFA/PUFA parameters at lower bounds), and a ‘diverse parameter’ scenario 4 (SFA parameter at lower bound, MUFA/PUFA parameters at upper bounds).

The two extreme scenarios result in strong variation of health pathway indicators between −93% and +86%, indicating that statistical uncertainty may lead to considerable variation in health pathway outcomes. Averted incident cases and averted premature deaths vary between 620–6333 (policy sim = 3570) and between 343–3281 (policy sim = 1861). However, no aggregate impacts change signs. Moreover, the simultaneous attainment of lower and upper confidence bounds for the three SFA, MUFA and PUFA parameter is unlikely. Hence, the fact that aggregate health pathway impacts of our ‘uniform parameter’ and ‘diverse parameter’ scenarios 3–4 only vary between −48% and +42% (online appendix C.1) can be taken as an indication that our results are likely to be fairly robust.

Nonetheless, the lower bound extreme sensitivity scenario raises the possibility of region-specific sign change: population impacts turn negative for Bangkok and the south region, and the overall net population expansion (980 person-years) is composed of an urban population reduction (−3276 person-years) and a rural population expansion (4256 person-years) (online appendix C.1). The particularly strong observed sensitivity of urban health impacts to uncertainty surrounding the SFA parameter suggests, that, while our sensitivity scenarios represent unlikely probabilistic events, the adverse policy health impact in urban south may not be an isolated incident. Hence, urban nutritional and health consequences of palm cooking oil consumption may need further scrutiny in future research.

Our two sets of labour market sensitivity analyses, including four scenarios with equidistant variation in workforce participation rates (65–80%, online Appendix C.2) and two scenarios with equidistant variation in retirement age (59–69 years, online appendix C.3) generally produce the same correlation patterns with aggregate indicators: mixed correlations with economic indicators (real GDP and private consumption: negative, real government consumption and investment: positive), positive correlations with clinical health outcomes (i.e. higher participation rates/retirement ages lead to smaller reductions in incident cases and fewer lives saved), and positive correlations with our environmental indicator (i.e. higher participation rates/retirement ages lead to larger increases in emissions). This suggests that reductions in labour market participation rate/retirement age parameters *ceteris paribus* produces more beneficial aggregate results and vice versa. In terms of real GDP, health pathway impacts vary between 17% and 29% (65%/59 yr) and −9% to −23% (80%/69 yr), while sales tax pathway impacts vary between 2.4% and 2.5% (65%/59 yr), and −1.6% to −1.8% (80%/69 yr). Health and nutrition indicators vary between −22% to +27%, while our environmental indicator varies from −4% to +5%. Overall, our labour market sensitivity specifications have small relative impacts on sales tax pathway indicators (economic, environment) and somewhat larger impacts on health pathway indicators (economic, nutritional, health), but results are generally robust.

Our three sets of palm cooking oil demand elasticity sensitivity analyses (online appendix C.4) include five scenarios with equidistant variation in own-price elasticities (−0.75 to −1.15; baseline ≈ −0.94 to −0.95), six scenarios with equidistant variation in cross-price elasticities between palm cooking oil and other edible oils (0.00–0.25; baseline ≈ 0.02–0.03), and five scenarios with equidistant variation in income elasticities (0.90–1.30; baseline ≈ 1.12–1.14). Sales tax pathway outcomes prove to be fairly robust with macroeconomic and environmental outcomes, generally, varying by ±10%, except for the own-price elasticity scenarios where macroeconomic outcomes, generally, vary by ±30% (real private and government consumption vary by 34–46% in the extreme scenario with an own-price elasticity of −0.75). The impact sensitivity to own-price elasticities stems from the fact, that required sales tax rates to achieve the policy target vary noticeably (46.3–61.7%). Hence, smaller absolute elasticities (less negative) may somewhat reduce the potency of our sales tax instrument. The (small) policy scenario health pathway outcomes proves to be more sensitive with macroeconomic and health outcomes varying by ±30% (income elasticity sensitivity), ±50% (own-price elasticity sensitivity), and factors of 16–18 (cross-price elasticity sensitivity). The upper bounds for the latter results are based on an extreme cross-price elasticity of 0.25. This is not likely to characterize Thai society. Nonetheless, cross-price elasticities in a reasonable range between 0.00 and 0.05 can change nutritional, health, and demographic outcomes by a factor of ±2, indicating that health pathway outcomes are very sensitive to the cross-price elasticity between palm cooking oil and other edible oils.

Finally, our set of iso-household consumption scenarios (online appendix C.5) include (1) an iso-household budget scenario (household-specific consumption budgets fixed relative to the CPI numeraire), (2) an iso-household real consumption scenario (household-specific real consumption fixed in terms of 2015 base year prices), and (3) a combined iso-household and government real consumption scenario (both household and government-specific real consumption fixed in terms of 2015 base period prices). In each case, non-distorting government transfers are used to maintain household (and government) consumption expenses and isolate efficiency losses on the producer side. Regardless of the type of compensating transfers, they turn out to leverage already positive nutritional, health, and demographic impacts through increased private consumption and improved dietary composition. While environmental harms persist, macroeconomic outcomes are affected quite differently. Unless government compensation measures are complemented by additional transfers funded by reduced government budgets, maintaining household real budgets (scenario 1) or real consumption expenses (scenario 2) lead to real GDP losses (USD −34.0bn and USD −2.9bn). However, if additional non-distortionary transfers are designed to fix real government consumption (scenario 3), real GDP could improve by USD 21.0bn over our time horizon. This indicates that Thailand is characterized by a second-best environment where higher palm oil sales taxes can improve economic efficiency and increase real GDP, and that this, depending on the scope of accompanying government transfers, may be achieved without reducing household welfare. This does, however, require that that the government is willing to fix their real consumption budget/reduce their nominal consumption budget.

We conclude that our tax instrument, combined with appropriate government compensating transfers and government real budget restraint, can be effective in achieving both nutritional and health targets and, at the same time, bring positive real GDP and (future) welfare outcomes for the Thai population. However, unless the government undertake complementary reductions in their budget (relative to the CPI numeraire), palm oil taxes may lead to declining household welfare over extended periods of time. Furthermore, adverse environmental outcomes persist, implying that, regardless of government action and the sign of welfare outcomes, the implementation of our proposed tax instrument continues to represent a case of policy trade-offs between nutritional and health benefits on the one hand, and environmental harms on the other.

## 5 Conclusion and discussion

### 5.1 Policy implications

Thailand is experiencing a nutritional transition, and, as part of this transition, palm oil consumption has been growing since the start of the millennium. Based on evidence linking palm oil consumption to adverse nutritional and clinical health outcomes, we have analysed a fiscal food policy scenario to halve energy intakes from palm cooking oil, based on a newly constructed 2016–2035 Thai MED-health model with a CVD-focused nutritional health pathway. Thai non-health policy priorities include palm oil consumption growth, sustainable agricultural development, and food safety, and our results indicate that these policy priorities are consistent. Attempts to limit palm oil consumption, through application of a sales tax instrument without government compensation, cause adverse welfare impacts, including particularly adverse welfare impacts on poor rural southern smallholder farmers in the Tapi River basin, and adverse environmental outcomes since oil palm plantations, once established, have favourable carbon sequestration characteristics compared to alternative crop uses. However, while discontinuation of the pursuit of palm oil consumption growth would be at odds with sustainable agricultural development and food safety, the accompanying dietary and health outcomes tell a somewhat different story. A product-specific sales tax would reduce patient mortality and incident cases from MI and stroke by 0.03–0.16%, translating into 13,621 person-years saved over the next 20 years.

A 54% palm oil sales tax was required to halve energy intakes from palm cooking oil consumption. Hence, Thai palm oil demand is fairly elastic, and, while our 54% tax rate can be categorized as fairly high, and certainly above the 15% minimum threshold for effectiveness alluded to in the fiscal food policy literature (Niebylski et al., 2015), our product-specific sales tax is more elastic and likely to be more efficient in achieving food policy targets compared to previously implemented types of fat taxes. A recent multi-country report, based on ecosystem value transfer techniques and non-market monetization of externalities, indicates that the value of natural capital externalities of oil palm production amounts to ≈52% in Thailand (Raynaud et al., 2016). While their results may seem to provide an additional rationale for our 54% palm oil sales tax, it is important to remember that production and sales taxes may have very different impacts depending on price transmission and price pass-through from producer to consumer prices. Furthermore, pricing metrics, based on non-market and global value transfer methods, may provide inaccurate country-specific value measurements. Hence, a recent Thailand-specific study, based on an alternative budget constraint method, found that external costs amount to 19% of the Thai retail price of palm biodiesel (Kaenchan and Gheewala, 2017). In contrast, our market-based approach is specifically designed to assess pecuniary economic and welfare impacts, and, at the same time, provide holistic and consistent modelling of a range of (non-monetized) multi-dimensional outcomes, including non-pecuniary nutritional, clinical health and environmental outcomes, and thereby allow policymakers to make informed policy choices based on established trade-offs (and based on their own implicit weighting of pecuniary and non-pecuniary outcomes).

In summary, our results indicate that (Thai) policymakers would do well in broadening their perspective and include health and nutrition impacts, alongside sustainability, food safety and rural livelihood considerations, when outlining (palm oil-related) food and agriculture strategies.

### 5.2 Future research

Our results highlight the limitations of fiscal food policy studies’ singular focus on nutritional and dietary outcomes. The narrow focus has a tendency of overemphasizing the benefits of fiscal food policies and overlook the important policy trade-offs to which fiscal instruments give rise. Researchers, focusing on highly policy-relevant fiscal food policy research, would do well in broadening their research to include, not only nutritional and dietary indicators, but also complementary non-food related policy indicators, in order to provide a broader holistic and more informative evidence base for (Thai) politicians to make informed decisions about fiscal food policies.

Whether a Thai palm cooking oil sales tax should be implemented is up to Thai policymakers, and should be decided by said policymakers on the basis of political priorities and weighing of policy impacts. The small economic impact of the health pathway does not, by itself, provide an economic rationale for undertaking the fiscal food policy intervention, and the modest clinical health impacts of our product-specific sales tax provides additional evidence that this is not a Columbus’ egg. However, sensitivity analyses suggest that modest variations in cross-price elasticities between palm oil and other edible oils can change health impacts by a factor two. The need for further studies of this issue is underlined by our iso-household and iso-government real consumption sensitivity analysis, which indicates that Thailand is characterized by a second-best environment where higher palm oil sales taxes improve economic efficiency, and where government transfers can fully compensate household welfare reductions, avoid crowding-out of investment, and ensure that introduction of our tax instrument yields positive real GDP gains (>USD 20bn) over our 20 year time horizon. If the government were to implement such a scenario (involving reduced nominal government/unchanged real household consumption expenses), there would be a stronger case for implementing palm oil sales taxes since both nutritional, health, and real GDP economic outcomes would improve, household welfare would be unchanged and only environmental impacts would worsen.

A more likely scenario, the cheapest in terms of compensation payments and one which doesn’t involve reductions in (possibly non-productive and non-welfare enhancing) government consumption budgets, would maintain real household consumption and result in real GDP losses of USD 2.9bn. From a public health perspective, the latter scenario, based on a USD 2.9bn real GDP loss and 13,621 person-years saved, suggests a cost-effectiveness ratio of >USD 200,000 per person-year saved. This exceeds standard cost-effectiveness thresholds for healthcare interventions in developed countries by an order of magnitude (and for developing countries by even more), and, since strong regulation to curb the growth of palm oil is not on the horizon and strategic rebalancing towards the use of palm oil for biofuels and oleochemicals can only be envisaged in the long term (Shankar et al., 2017), it may be worth investigating other behavioural interventions, as well as non-behavioural non-lifestyle interventions, which may compare favourably to this scenario and be more effective in addressing CVD-illness in Thailand. Non-pecuniary valuation of long run GHG emission reduction benefits according to World Bank Guidelines (WB, 2017) would not change this conclusion. The suggested valuation at USD 50–100 per tonne of CO_2_-eq GHG emissions (WB, 2017) would value the 7.5 Mt CO_2_-eq emission reduction at <USD 750mn, and reduce the efficiency cost by less than 30% leaving the implied cost-effectiveness ratio at >USD 150,000 per person-year saved.

Apart from strong sensitivity of health pathway outcomes to cross-price elasticities between palm oil and other edible oils, our results were generally found to be robust. However, some of our nutritional sensitivity analyses indicated that the adverse policy health impact in urban south may not be an isolated incident, and that the width of impact bounds was driven by urban nutritional sensitivity to the SFA parameter of the serum cholesterol specification. While unlikely to occur, these results indicate that urban health consequences of palm cooking oil consumption, including the question of how urban demand substitution and dietary composition interacts with nutritional transmission, may need closer scrutiny in future research. Future research should focus on resolving the ”Saturated Fat Controversy”, which has questioned the link between SFA intakes and clinical health outcomes, and on expanding the focus of the nutrition-related serum cholesterol pathway literature from a singular focus on fatty acid composition to a broader understanding of how increased energy intake levels and obesity affects serum cholesterol outcomes. Due to our focus on non-obesity related CVD illness and the observed policy-related increases in energy intakes, our analyses may have slightly overstated health benefits in urban areas, but this is likely to be balanced by understated health benefits in food insecure rural areas.

### 5.3 Final caveat and key lessons

A final caveat is in order. Extrapolation of our results to make inference about the current Thai Oil Palm and Palm Oil Industries Development Plan for 2015–2026 and argue for further oil palm expansion should be made with caution. While there remains some potential for marginal land conversion, historical forest burning in the North, related to increasing returns to maize cultivation, indicates, that future large-scale Thai oil palm expansion, based on increased productivity and returns to Southern farmers, may lead to similar direct or indirect deforestation which could affect the sustainability of such a strategy.

Thinking more broadly about other palm oil producing and consuming countries, our product-specific sales tax should be effective in achieving product-specific food policy targets among consumers (unless own-price elasticities are exceedingly low). Whether our surprising finding, that Thailand is characterized by a second-best environment, where increased sales taxes produce efficiency gains, applies to other countries, is an empirical matter. In any case, lack of complementary government compensation policies and consumption adjustment could cause sales taxes to reduce household welfare for extended periods of time, and overall dietary change and sustainability outcomes would depend on country-specific substitution patterns in food demand and production-related direct and indirect deforestation. While our multi-dimensional set of policy impacts are bound to vary from country to country, sustainability issues could be more important and potentially tip the balance of policy trade-offs in net-producing countries such as Indonesia and Malaysia. Similarly, dietary and health benefits could be more important and potentially tip the balance in countries with little or no domestic production such as India and China. The key lesson is that national policy makers, when deciding about palm oil development strategies and the possible use of product-specific fiscal food policy instruments, should give due attention to the broader holistic set of dietary, health, and environmental outcomes alongside economic impacts.

## Footnotes

1 Caregiver time losses for MI were considered small due to short illness duration, and therefore not included in the study. E.g. the 2010 GBD study only attributes MI disease burden to the first 28 days of illness (WHO, 2013).

## References

[b0005] Aekplakorn W., Chariyalertsak S., Kessomboon P., Sangthong R., Inthawong R., Putwatana P., Taneepanichskul S. (2011). Prevalence and management of diabetes and metabolic risk factors in Thai adults. Diabetes Care.

[b0010] Alderman, H., Sahn, D.E. 2016. Public and private returns to investing in nutrition. In: Komlos J., Kelly, I.R. (Eds.), Chapter 20 in Oxford Handbook of Economics and Human Biology.

[b0015] Allais O., Bertail P., Nichèle V. (2010). The effects of a fat tax on French households’ purchases: a nutritional approach. Am. J. Agric. Econ..

[b0020] Anukoolsawat P., Sritara P., Teerawattananon Y. (2006). Costs of lifetime treatment of acute coronary syndrome at Ramathibodi Hospital. Thai Heart J..

[b0025] Basu S., Babiarz K.S., Ebrahim S., Vellakkal S., Stuckler D., Goldhaber-Fiebert J.D. (2013). Palm oil taxes and cardiovascular disease mortality in India: economic-epidemiologic model. BMJ.

[b0030] Beluri M.A. (2016). Association of specific dietary fats with mortality. To the editor. JAMA Intern. Med..

[b0035] Cahyadi E.R., Waibel H. (2013). Is contract farming in the Indonesian oil palm industry pro-poor?. J. Southeast Asian Econ..

[b0040] Chouinard, H.H., Davis, D.E., LaFrance, J.T., Perloff, J.M., 2007. Fat taxes: big money for small change. Forum Health Econ. Policy 10(2) Article 2:1–28.

[b0045] Ciscar J.-C., Iglesias A., Feyenc L., Szabóa L., van Regemortera D., Amelunge B., Nicholls R., Watkiss P., Christensen O.B., Dankersc R., Garrotek L., Goodess C.M., Hunt A., Moreno A., Richards J., Soria A. (2010). Physical and economic consequences of climate change in Europe. Proc. Natl. Acad. Sci..

[b0050] Carlson K.M., Curran L.M., Asner G.P., Pittman A.M., Trigg S.N., Adeney J.M. (2012). Carbon emissions from forest conversion by Kalimantan oil palm plantations. Nat. Clim. Change.

[b0055] Dallinger, J., 2011. Oil palm development in Thailand: economic, social and environmental considerations. In: Colchester, M., Chao, S. (Eds.), Chapter 1 in Oil Palm Expansion in South East Asia: Trends and implications for local communities and indigenous peoples. Forest Peoples Programme and SawitWatch.

[b0060] Deaton A., Arora R. (2009). Life at the top: the benefits of height. Econ. Hum. Biol..

[b0065] Dubois V., Breton S., Linder M., Fanni J., Parmentier M. (2007). Fatty acid profiles of 80 vegetable oils with regard to their nutritional potential. Eur. J. Lipid Sci. Technol..

[b0070] Euler M., Krishna V., Schwarze S., Siregar H., Qaim M. (2017). Oil palm adoption, household welfare, and nutrition among smallholder farmers in Indonesia. World Dev..

[b0075] FAO, 2017. Electronic crop data. FAOSTAT. Food and Agriculture Organization of the United Nations: Rome. URL: <http://www.fao.org/faostat/en/#data/QC> (accessed 6. April 2017).

[b0080] Golan A., Judge G., Robinson S. (1994). Recovering information from incomplete or partial multisectoral economic data. Rev. Econ. Stat..

[b0085] Green R., Alston J.M. (1990). Elasticities in AIDS models. Am. J. Agric. Econ..

[b0090] Green R., Alston J.M. (1991). Elasticities in AIDS models: a clarification and extension. Am. J. Agric. Econ..

[b0095] Harding M., Lovenheim M. (2017). The effect of prices on nutrition: comparing the impact of product-and nutrient-specific taxes. J. Health Econ..

[b0100] Ikhsan S., Anindita R., Hanani N., Koestiono D. (2016). The change in world price and export tax of crude palm oil and their impact on the economy and welfare in Indonesia: using a computable general equilibrium (CGE) model. Russ. J. Agric. Socio-econ. Sci..

[b0105] Jefferis K., Kinghorn A., Siphambe H., Thurlow J. (2008). Macroeconomic and household-level impacts of HIV/AIDS in Botswana. AIDS.

[b0110] Jensen H.T., Keogh-Brown M.R., Smith R.D., Chalabi Z., Dangour A.D., Davies M., Edwards P., Garnett T., Givoni M., Griffiths U., Hamilton I., Jarrett J., Roberts I., Wilkinson P., Woodcock J., Haines A. (2013). The importance of health co-benefits in macroeconomic assessments of UK Greenhouse Gas emission reduction strategies. Clim. Change.

[b0120] Jitnarin N., Kosulwat V., Rojroongwasinkul N., Boonpraderm A., Haddock C.K., Poston W.S.C. (2010). Risk factors for overweight and obesity among Thai adults: results of the National Thai Food Consumption Survey. Nutrients.

[b0125] Kaenchan P., Gheewala S.H. (2017). Budget constraint and the valuation of environmental impacts in Thailand. Int. J. Life Cycle Assessment.

[b0130] Kambou G., Devarajan S., Over M. (1992). The economic impact of AIDS in an African country: simulations with a computable general equilibrium model of cameroon. J. Afr. Econ..

[b0135] Kelly M., Banwell C., Dixon J., Seubsman S., Yiengprugsawan V., Sleigh A. (2010). Nutrition transition, food retailing and health equity in Thailand. Aust. Epidemiol..

[b0140] Khiaocharoen O., Pannarunothai S., Zungsontiporn C. (2012). Cost of acute and sub-acute care for stroke patients. J. Med. Assoc. Thai..

[b0145] Kim S.-R., Chern W.S. (1999). Alternative measures of health information and demand for fats and oils in Japan. J. Consumer Affairs.

[b0150] Koh L.P., Wilcowe D.C. (2008). Is oil palm agriculture really destroying tropical biodiversity?. Conserv. Lett..

[b0155] Kosulwat V., Rojroongwasinkul N., Boonpraderm A., Viriyapanich T., Jitnarin N., Sornkaew N., Vanicchakul C. (2006). Food Consumption Data of Thailand (in Thai) National Bureau of Agricultural Commodity and Food Standards.

[b0160] Li J.C. (2002). Including the feedback of local health improvement in assessing costs and benefits of GHG reduction. Rev. Urban Regional Dev. Stud..

[b0165] Li J.C. (2005). Is there a trade-off between trade liberalization and environmental quality? A CGE assessment on Thailand. J. Environ. Dev..

[b0170] Lippe R.S., Isvilanonda S. (2010). Food demand elasticities among urban households in Thailand. Thammasat Econ. J..

[b0175] Mensink R.P., Zock P.L., Kester A.D.M., Katan M.B. (2003). Effects of dietary fatty acids and carbohydrates on the ratio of serum total to HDL cholesterol and on serum lipids and apolipoproteins: a meta-analysis of 60 controlled trials. Am. J. Clin. Nutr..

[b0180] Minot N.W. (1998). Distributional and nutritional impact of devaluation in Rwanda. Econ. Dev. Cult. Change.

[b0185] NESDB, 2013a. Population projection for Thailand 2010-2040. Office of the National Economic and Social Development Board. Bangkok.

[b0190] NESDB, 2013b. Population projections at regional and municipality level 2010–2035. Electronic data. Office of the National Economic and Social Development Board, Bangkok.

[b0195] NESDB. 2015. Electronic data. 2007 Social Accounting Matrix for Thailand. National Economic and Social Development Board, Bangkok.

[b0200] NHESO (2009). Thailand National Health and Examination Survey 2008–2009.

[b0205] Niebylski M.L., Redburn K.A., Duhaney T., Campbell N.R. (2015). Healthy food subsidies and unhealthy food taxation: a systematic review of the evidence. Nutrition.

[b0210] NSO (2008). Key statistics of Thailand 2003–2007.

[b0215] NSO, 2014. 2011 Household Socio-Economic Survey. Electronic data. National Statistical Organization Thailand, Bangkok.

[b0220] OEPP, 2010. Thailand’s Second National Communication under the United Nations Framework Convention on Climate Change. Ministry of Natural Resources and Environment, Bangkok. URL: <http://unfccc.int/essential_background/library/items/3599.php?rec=j&priref=7460#beg> (accessed 25. January 2017).

[b0225] Pauw K., Thurlow J. (2011). Agricultural growth, poverty, and nutrition in Tanzania. Food Policy.

[b0230] Petchseechoung, W., 2016. Oil Palm Industry. Thailand Industry Outlook 2016–18. Krungsri Research. URL: <https://www.krungsri.com/bank/getmedia/0d7baae2-f679-4269-b461-89937533c9e1/IO_OilPalm_2016_EN.aspx> (accessed 6. April 2017).

[b0235] Ravnskov U., Okuyama H., Harcombe Z. (2016). Association of specific dietary fats with mortality. To the editor. JAMA Intern. Med..

[b0240] Raynaud, J., Fobelets, V., Georgieva, A., Joshi, S., Kristanto, L., de Groot Ruiz, A., Bullock, S., Hardwicke, R., 2016. Improving business decision making: valuing the hidden costs of production in the palm oil sector. A study for The Economics of Ecosystems and Biodiversity for Agriculture and Food (TEEBAgriFood) Program. URL: <http://www.teebweb.org/wp-content/uploads/2016/12/TEEBAgriFood_PalmOil_Report.pdf>. (accessed 10. September 2018).

[b0245] Rewtarkulpaiboon, L., 2015. Thai Palm Oil Industry and Roadmap for Implementation of Strategic Agricultural Crops. Electronic slides from Asia Palm Oil Conference presentation. Office of Agricultural Economics, Ministry of Agriculture and Cooperatives. URL: <http://www.palmoil-conference.com/upload/file/1%20Mr.Lersak%20Rewtarkulpaiboon_Ministry%20of%20Agriculture%20and%20Cooperatives_TH.pdf> (accessed 10. May 2017).

[b0250] Riewpaiboon A., Riewpaiboon W., Ponssongnern K., van den Berg B. (2009). Economic valuation of informal care in Asia: a case study of care for disabled stroke survivors in Thailand. Soc. Sci. Med..

[b0255] Robinson S., Cattaneo A., El-Said M. (2001). Updating and estimating a social accounting matrix using cross entropy methods. Econ. Syst. Res..

[b0260] Rutten M., Achterbosch T.J., de Boer I.M., Cuaresma J.C., Geleijnse J.M., Havlík P., Heckelei T., Ingram J., Leip A., Marette S., van Meijl H., Soler L.-G., Swinnen J., van't Veer P., Vervoort J., Zimmermann A., Zimmermann K.L., Zurek M. (2018). Metrics, models and foresight for European sustainable food and nutrition security: the vision of the SUSFANS project. Agric. Syst..

[b0265] Saswattecha K., Hein L., Kroeze C., Jawjit W. (2016). Effects of oil palm expansion through direct and indirect land use change in Tapi river basin, Thailand. Int. J. Biodiversity Sci. Ecosyst. Services Manage..

[b0270] Saswattecha K., Kroeze C., Jawjit W., Hein L. (2015). Assessing the environmental impact of palm oil produced in Thailand. J. Clean. Prod..

[b0275] Shankar B., Thaiprasert N., Gheewala S., Smith R.D. (2017). Policies for healthy and sustainable edible oil consumption: a stakeholder analysis for Thailand. Public Health Nutr..

[b0280] Sheil D., Casson A., Meijaard E., van Noordwijk M., Gaskell J., Sunderland-Groves J., Wertz K., Kanninen M. (2009). The impacts and opportunities of oil palm in Southeast Asia. What do we know and what do we need to know? CIFOR Occasional paper No. 51.

[b0285] Silalertruksa T., Gheewala S.H. (2012). Environmental sustainability assessment of palm biodiesel production in Thailand. Energy.

[b0290] Silalertruksa T., Gheewala S.H. (2012). Food, fuel, and climate change. J. Ind. Ecol..

[b0295] Silalertruksa T., Gheewala S.H., Pongpat P., Kaenchan P., Permpool N., Lecksiwilai N., Mungkung R. (2017). Environmental sustainability of oil palm cultivation in different regions of Thailand: greenhouse gases and water use impact. J. Clean. Prod..

[b0300] SMILING, 2016. Electronic data. Sustaining Micronutrient Interventions to Control Deficiencies and Improve Nutritional Status and General Health in Asia project. Institut de Recherche pour le Developpement, France. URL: <http://www.nutrition-smiling.eu/> (accessed 15. October 2015).

[b0305] Smith L.C., Haddad L. (2002). How potent is economic growth in reducing undernutrition? What are the pathways of impact? New cross-country evidence. Econ. Dev. Cult. Change.

[b0310] Stafford N. (2012). Denmark cancels “fat tax” and shelves “sugar tax” because of threat of job losses. Br. Med. J..

[b0315] Suebpongsakorn A. (2008). The Development of a Multisectoral Model for the Thai Economy (MUTE).

[b0320] Thurlow J., Gow J., George G. (2009). HIV/AIDS, growth and poverty in KwaZulu-Natal and South Africa: an integrated survey, demographic and economy-wide analysis. J. Int. AIDS Soc..

[b0325] Timilsina G.R., Mevel S. (2013). Biofuels and climate change mitigation. A CGE analysis incorporating land-use change. Environ. Resource Econ..

[b0330] UN, 2015. Electronic data. World Population Prospects, the 2015 Revision. United Nations. URL: <http://esa.un.org/unpd/wpp/index.htm> (accessed 14. September 2015).

[b0335] USDA, 2014. Electronic data. USDA food composition database. United States Department of Agriculture Agricultural Research Service, United States. URL: <https://ndb.nal.usda.gov/ndb/> (accessed 17. September 2014).

[b0340] Villoria N.B., Golub A., Byerlee D., Stevenson J. (2013). Will yield improvements on the forest frontier reduce greenhouse gas emissions? A global analysis of oil palm. Am. J. Agric. Econ..

[b0345] Wang D.D., Li Y., Chiuve S.E., Stampfer M.J., Manson J.E., Rimm E.B., Willett W.C., Hu F.B. (2016). Association of specific dietary fats with total and cause-specific mortality. JAMA Int. Med..

[b0350] Wang D.D., Willet W.C., Hu F.B. (2016). Association of specific dietary fats with mortality. In reply. JAMA Int. Med..

[b0355] WB, 2017. Guidance note on shadow price of carbon in economic analysis (English). World Bank Group, Washington, D.C. <http://documents.worldbank.org/curated/en/621721519940107694/Guidance-note-on-shadow-price-of-carbon-in-economic-analysis>.

[b0360] WHO, 2003. Diet, Nutrition and the Prevention of Chronic Diseases. Technical Report 916 (160pp). 12768890

[b0365] WHO, 2013. WHO methods and data sources for global burden of disease estimates 2001–2011. Global Health Estimates Technical Paper WHO/HIS/HSI/GHE/2013.4. WHO, Geneva.

[b0370] WHO, 2015. Thailand: WHO statistical profile. World Health Organization, Geneva. URL: <http://www.who.int/gho/countries/tha.pdf> (accessed 1. May 2017).

[b0375] Wianwiwat S., Asafu-Adjaye J. (2013). Is there a role for biofuels in promoting energy self sufficiency and security? A CGE analysis of biofuel policy in Thailand. Energy Policy.

[b0380] Yen S.T., Chern W.S. (1992). Flexible demand systems with serially correlated errors: fat and oil consumption in the United States. Am. J. Agric. Econ..

