# Supplementary material for: Palm oil and dietary change: Application of an integrated macroeconomic, environmental, demographic, and health modelling framework for Thailand
Source: Food Policy. 2019 Feb;83:92–103. doi: 10.1016/j.foodpol.2018.12.003 (PMC6472326; doi:10.1016/j.foodpol.2018.12.003)
Supplement: Supplementary data 1 [file mmc1.docx]

# Appendix A. Lookup tables for clinical health outcomes, and disability weights.

## A.1. Lookup tables for clinical health outcomes

Simulated age- and gender-specific lookup tables for clinical outcomes were also based on biomarker data from the 2008-2009 Thai-HES Survey (NHESO 2009). Random sampling of 10,000 simulated individuals from normal distributions with means and standard deviations derived from the Thai-HES survey, was complemented by calculation of relative hazards for key events including non-fatal MI, non-fatal stroke, fatal MI, fatal stroke using an established empirical methodology (Lim et al. 2007) and relying on previously established log relative risks (Lewington et al. 2007). Individual risk was, subsequently, calculated from a competing risks algorithm accounting for alternative causes of fatality (Prentice et al. 1978), and relying on Global Burden of Disease data on rates of MI and stroke (Lozano et al. 2012) and WHO data on all-cause mortality (WHO 2014).

Relative hazards were corrected for a range of co-risk factors including systolic blood pressure, total cholesterol, tobacco smoking, diabetes, and cardiovascular event history, and stratified according to gender, rural/urban location, and 13 five-year age groups including 11 working age groups (15-64), and two retirement age groups ‘65-69’ and ‘70+’. Lookup tables were established for incidence and excess mortality of two cholesterol-related CVD illnesses, myocardial infarction (MI) and stroke, and the chosen biomarker support range ([2.0;7.0]) was chosen so as to encompass the full range of potential consumer-specific biomarker levels. ^[[1]](#footnote-1)^ Altogether, 11 sets of lookup tables were established, covering 10 equidistant intervals for the biomarker. For the current paper, the health module utilized a set of fitted 10^th^ degree polynomials for calculation of gender, rural/urban, and age group-specific clinical health outcomes covering incidence and excess mortality rates for MI and stroke.^[[2]](#footnote-2)^

## A.2. Disability weights

In our study, we employ Years Lost due to Disability (YLD) as our measure of patient morbidity. This section explains how we derived our set of Thai-specific disability weights for, respectively, stroke and Myocardial Infarction (MI). The severity of stroke differs strongly between cases. This is evidenced by the distribution of Thai stroke cases over four Barthel index-based disability categories (Riewpaiboon et al. 2009). In order to capture the distribution of case severity, the four Barthel-categories of the latter paper (all defined by Barthel index scores<95) were mapped to the four least severe categories (out of five) of 2010 Global Burden of Disease YLD disability weights for stroke (WHO 2013). Together, the two distributions of Thai-specific case severity shares and severity-specific YLD disease burdens were used to compute average Thai YLD disability weights for stroke (to be used for our patient disease burden calculations). In terms of MI, the two categories of 2010 Global Burden of Disease YLD disability weights (ibid.) are defined for stage 1 (first 2 days, most severe) and stage 2 (following 26 days, much less severe), and we therefore decided to use the relative duration of the two stages as shares for computing an average YLD disability weight for MI.

# Appendix B. Demographic Module specification.

The demographic module was designed to mirror the population stratification of the health and nutrition modules. A set of 2010-35 Thai regional population projections was obtained from the National Economic and Social Development Board (NESDB 2013a, NESDB 2013b). The projections were age- and gender-specific (5 year age groups), and covered 8 regions which mapped one-to-one with our 5 region aggregation and allowed for rural/urban population splits of all regions except Bangkok. The aggregated data set was subsequently employed to calibrate our demographic model, encompassing a compact set of equations for calculating Births, Deaths, Migration (Migr), and Population (Pop) levels for our nine households (H), 20 time periods (T), thirteen quinquennial age categories (AGE) and two gender types (GEN):

(3) Births_h,gen,t_ = sexratio_gen_*Σ_(gen,age)|gen=’female’, age ε [15;49]_asfr_age,t_*Pop_h,gen,age,t-1_, h ε H, t ε T, gen ε GEN

(4) Deaths_h,gen,age,t_ = µ_h,gen,age,t_*Pop_h,gen,age,t-1_, h ε H, g ε GEN, t ε T, age ε AGE

(5) Migr_h,gen,age,t_ = α^imigr^_h,gen,age,t_*(1-µ_h,gen,age,t_)*Pop_h,gen,age,t-1_, h ε H, g ε GEN, t ε T, age ε AGE

(6a) Pop_h,gen,age,t|age=0-4_ = (1-p^trans^_h,gen,age,t_)*(1-α^imigr^_h,gen,age,t_)*(1-µ_h,gen,age,t_)*Pop_h,gen,age,t-1_

+ Births_h,gen,t_, h ε H, g ε GEN, t ε T

(6b) Pop_h,gen,age,t|age>0-4 and age<70+_ = p^trans^_h,gen,age-1,t_*(1-α^imigr^_h,gen,age-1,t_)*(1-µ_h,gen,age-1,t_)*Pop_h,gen,age-1,t-1_

+ (1-p^trans^_h,gen,age,t_)*(1-α^imigr^_h,gen,age,t_)*(1-µ_h,gen,age,t_)*Pop_h,gen,age,t_,

h ε H, g ε GEN, t ε T, age ε AGE\{’0-4’, ’70+’}

(6c) Pop_h,gen,age,t|age=70+_ = p^trans^_h,gen,age-1,t_*(1-α^imigr^_h,gen,age-1,t_)*(1-µ_h,gen,age-1,t_)*Pop_h,gen,age,t-1_

+ (1-α^imigr^_h,gen,age,t_)*(1-µ_h,gen,age,t_)*Pop_h,gen,age,t-1_, h ε H, g ε GEN, t ε T

where sexratio_gen,t_ are gender- and household-specific sex-ratios at birth, asfr_age,t_ are age-specific fertility rates, µ_h,gen,age,t_ are age-, gender- and household-specific mortality rates, α^imigr^_h,gen,age,t_ are age- gender- and household-specific net immigration rates, and p^trans^_h,gen,age,t_ are age-, gender- and household-specific annual transition probabilities between 5 year age groups. The 2010-35 population projection data set did not include information about underlying demographic parameter assumptions. Instead, age- and gender-specific parameters were obtained from the 2015 Revision of UN population projections (UN 2015), and the demographic model calibration was completed through dynamic calibration of transition probabilities between 5 year age groups (p^trans^_h,gen,age,t_).^[[3]](#footnote-3)^

# Appendix C. Sensitivity Analyses.

## C.1. Sensitivity to cholesterol ratio biomarker equation parameters

The nutrition module computes household-specific average energy intake shares from SFA, MUFA and PUFA, and, subsequently, calculates average and stratified household-specific changes in Total-to-HDL serum cholesterol ratio biomarker (ΔC) levels from formula (C.1.1):

(C.1.1) ΔC_h,t_ = α^SFA^ ΔSFA_h,t_ + α^MUFA^ ΔMUFA_h,t_ + α^PUFA^ ΔPUFA_h,t_ h ε H, t ε T

The above equation was parameterized based on estimated correlations between changes in nutritional intakes and consumers’ serum cholesterol levels (Mensink et al. 2003). The latter study provided both central estimates and confidence bounds (see Table C.1.1).

| **Table C.1.1. Total:HDL cholesterol ratio elasticities wrt. SFA, MUFA and PUFA** | | | | |
| --- | --- | --- | --- | --- |
|  | point estimate |  | Confidence interval | |
|  |  |  | lower bound | upper bund |
| α^SFA^ | 0.003 |  | -0.008 | 0.013 |
| α^MUFA^ | -0.026 |  | -0.017 | -0.035 |
| α^PUFA^ | -0.032 |  | -0.022 | -0.042 |
| Source: Mensink et al. 2003. | | | | |

Mensink et al’s point estimates were used to parameterize the model, while the lower and upper bounds was used to simulate four sensitivity scenarios (see table C.1.2). Results are provided in Tables C.1.3-C.1.5.

| **Table C.1.2. Sensitivity Scenarios: Variation in MENSINK parameters** | |
| --- | --- |
| Scenarios | Description |
| Policy Scenario | -50% average household consumption of palm cooking oil (central MENSINK parameter estimates) |
| Sensitivity Scenario 1 | MENSINK parameter lower confidence bound estimates  (-2 standard deviations) |
| Sensitivity Scenario 2 | MENSINK parameter upper confidence bound estimates  (+2 standard deviations) |
| Sensitivity Scenario 3 | SFA upper confidence bound (+2 standard deviations)  MUFA/PUFA lower confidence bound (-2 standard deviations) |
| Sensitivity Scenario 4 | SFA lower confidence bound (-2 standard deviations)  MUFA/PUFA upper confidence bound (+2 standard deviations) |
| Instrument | Product-specific sales tax on palm cooking oil |

| **Table C.1.3. Sensitivity Analysis: Variation in MENSINK parameters, SFA vs MUFA/PUFA, central ± 2 standard deviations (economy)** | | | | | | | | | | | | | |
| --- | --- | --- | --- | --- | --- | --- | --- | --- | --- | --- | --- | --- | --- |
|  | Policy Scenario (central parms) |  | Sensitivity Scenario 1. (low parms: -2 std. dev.) | |  | Sensitivity Scenario 2.  (high parms: +2 std. dev.) | |  | Sensitivity Scenario 3.  (high SFA: + 2 std.dev.) (low MUFA/PUFA: -2 std. dev.) | |  | Sensitivity Scenario 4.  (low SFA: -2 std. dev.)  (high MUFA/PUFA: +2 std. dev.) | |
| ***Real GDP & sales tax (cumulative and long run indicators)*** | | | | | | | | | | | | | |
|  | mn USD |  | mn USD | %-change |  | mn USD | %-change |  | mn USD | %-change |  | mn USD | %-change |
| Δreal GDP (cumulative) | 25,145 |  | 25,063 | -0.3% |  | 25,222 | 0.3% |  | 25,177 | 0.1% |  | 25,108 | -0.1% |
| - health pathway (cumulative) | 95 |  | 13 | -85.9% |  | 172 | 80.2% |  | 127 | 33.5% |  | 58 | -39.2% |
| - sales tax pathway (cumulative) | 25,050 |  | 25,049 | 0.0% |  | 25,050 | 0.0% |  | 25,049 | 0.0% |  | 25,050 | 0.0% |
|  | mn USD |  | mn USD | %-change |  | mn USD | %-change |  | mn USD | %-change |  | mn USD | %-change |
| Δreal GDP (cumulative) | 25,145 |  | 25,063 | -0.3% |  | 25,222 | 0.3% |  | 25,177 | 0.1% |  | 25,108 | -0.1% |
| - Private Consumption (cumulative) | -9,882 |  | -9,923 | 0.4% |  | -9,843 | -0.4% |  | -9,865 | -0.2% |  | -9,901 | 0.2% |
| - Government Consumption (cumulative) | 8,633 |  | 8,670 | 0.4% |  | 8,599 | -0.4% |  | 8,621 | -0.1% |  | 8,648 | 0.2% |
| - Investment (cumulative) | 26,394 |  | 26,317 | -0.3% |  | 26,466 | 0.3% |  | 26,421 | 0.1% |  | 26,361 | -0.1% |
| - Exports (cumulative) | 35,857 |  | 35,778 | -0.2% |  | 35,931 | 0.2% |  | 35,887 | 0.1% |  | 35,822 | -0.1% |
| - Imports (cumulative) | 35,857 |  | 35,778 | -0.2% |  | 35,931 | 0.2% |  | 35,887 | 0.1% |  | 35,822 | -0.1% |
|  | %-points |  | %-points | %-point change |  | %-points | %-point change |  | %-points | %-point change |  | %-points | %-point change |
| Δsales tax (long run, 2035) | 53.5% |  | 53.5% | 0.00% |  | 53.5% | 0.00% |  | 53.5% | 0.00% |  | 53.5% | 0.00% |
| Δinvestment price index (long run, 2035) | -0.8% |  | -0.8% | 0.00% |  | -0.8% | 0.00% |  | -0.8% | 0.00% |  | -0.8% | 0.00% |
| Δreal exchange rate (long run, 2035) | -0.9% |  | -0.9% | 0.00% |  | -0.9% | 0.00% |  | -0.9% | 0.00% |  | -0.9% | 0.00% |
| ***Real Consumption (cumulative indicators)*** | | | | | | | | | | | | | |
|  | mn USD |  | mn USD | %-change |  | mn USD | %-change |  | mn USD | %-change |  | mn USD | %-change |
| Δreal Household Consumption | -9,882 |  | -9,923 | 0.4% |  | -9,843 | -0.4% |  | -9,865 | -0.2% |  | -9,901 | 0.2% |
| - sales tax pathway | -9,930 |  | -9,930 | 0.0% |  | -9,929 | 0.0% |  | -9,930 | 0.0% |  | -9,929 | 0.0% |
| - Bangkok^1^ | -362 |  | -362 | 0.0% |  | -362 | 0.0% |  | -362 | 0.0% |  | -362 | 0.0% |
| - Central region (exc Bangkok)^1^ | -3,336 |  | -3,336 | 0.0% |  | -3,336 | 0.0% |  | -3,336 | 0.0% |  | -3,336 | 0.0% |
| - North region^1^ | -935 |  | -935 | 0.0% |  | -935 | 0.0% |  | -935 | 0.0% |  | -935 | 0.0% |
| - Northeast region^1^ | -982 |  | -982 | 0.0% |  | -982 | 0.0% |  | -982 | 0.0% |  | -982 | 0.0% |
| - South region^1^ | -4,315 |  | -4,315 | 0.0% |  | -4,315 | 0.0% |  | -4,315 | 0.0% |  | -4,315 | 0.0% |
| - health pathway | 48 |  | 6 | -86.7% |  | 87 | 80.9% |  | 65 | 34.5% |  | 29 | -40.3% |
| - Bangkok^1^ | 8 |  | -1 | -107.7% |  | 16 | 99.7% |  | 13 | 59.8% |  | 3 | -67.6% |
| - Central region (exc Bangkok)^1^ | 19 |  | 6 | -67.9% |  | 31 | 64.0% |  | 21 | 11.8% |  | 16 | -15.7% |
| - North region^1^ | 7 |  | 1 | -86.0% |  | 12 | 80.3% |  | 9 | 33.5% |  | 4 | -39.3% |
| - Northeast region^1^ | 9 |  | 2 | -82.1% |  | 16 | 76.9% |  | 12 | 28.9% |  | 6 | -34.2% |
| - South region^1^ | 5 |  | -2 | -130.1% |  | 12 | 119.9% |  | 10 | 86.7% |  | 0 | -96.9% |
| Note: Own calculations. ^1^ Regional consumption %-impacts calculated as share of projected regional totals. | | | | | | | | | | | | | |

| **Table C.1.4. Sensitivity Analysis: Variation in MENSINK parameters, SFA vs MUFA/PUFA, central ± 2 standard deviations (nutrition, biomarker, health)** | | | | | | | | | | | | | |
| --- | --- | --- | --- | --- | --- | --- | --- | --- | --- | --- | --- | --- | --- |
|  | Policy Scenario (central parms) |  | Sensitivity Scenario 1. (low parms: -2 std. dev.) | |  | Sensitivity Scenario 2. (high parms: +2 std. dev.) | |  | Sensitivity Scenario 3.  (high SFA: + 2 std.dev.) (low MUFA/PUFA: -2 std. dev.) | |  | Sensitivity Scenario 4.  (low SFA: -2 std. dev.)  (high MUFA/PUFA: +2 std. dev.) | |
| ***Nutrition (long run indicators)*** |  |  |  |  |  |  |  |  |  |  |  |  |  |
|  | %-points |  | %-points | %-change |  | %-points | %-change |  | %-points | %-change |  | %-points | %-change |
| ΔSFA energy intake share (long run, 2035) | -0.322% |  | -0.322% | 0.0% |  | -0.322% | 0.0% |  | -0.322% | 0.0% |  | -0.322% | 0.0% |
| ΔMUFA energy intake share (long run, 2035) | -0.164% |  | -0.164% | 0.0% |  | -0.164% | 0.0% |  | -0.164% | 0.0% |  | -0.164% | 0.0% |
| ΔPUFA energy intake share (long run, 2035) | 0.298% |  | 0.297% | 0.0% |  | 0.298% | 0.0% |  | 0.298% | 0.0% |  | 0.297% | 0.0% |
| ***Biomarker (cumulative indicators)*** |  |  |  |  |  |  |  |  |  |  |  |  |  |
|  | cum. chg. |  | cum. chg. | %-change |  | cum. chg. | %-change |  | cum. chg. | %-change |  | cum. chg. | %-change |
| ΔTotal-to-HDL cholesterol ratio | -0.101 |  | -0.005 | -95.1% |  | -0.190 | 88.5% |  | -0.146 | 44.6% |  | -0.049 | -51.3% |
| ***Health (cumulative indicators)*** |  |  |  |  |  |  |  |  |  |  |  |  |  |
|  | cases |  | cases | %-change |  | cases | %-change |  | cases | %-change |  | cases | %-change |
| ΔPatient Incident Cases | -3,570 |  | -620 | -82.6% |  | -6,333 | 77.4% |  | -4,622 | 29.5% |  | -2,325 | -34.9% |
| - myocardial infarction | -2,704 |  | -466 | -82.8% |  | -4,802 | 77.6% |  | -3,501 | 29.5% |  | -1,759 | -35.0% |
| - stroke | -866 |  | -153 | -82.3% |  | -1,531 | 76.8% |  | -1,121 | 29.5% |  | -566 | -34.7% |
| ΔPatient premature deaths | -1,861 |  | -343 | -81.6% |  | -3,281 | 76.3% |  | -2,389 | 28.3% |  | -1,237 | -33.6% |
| - myocardial infarction | -1,560 |  | -285 | -81.7% |  | -2,755 | 76.6% |  | -2,002 | 28.3% |  | -1,035 | -33.7% |
| - stroke | -301 |  | -58 | -80.7% |  | -527 | 74.7% |  | -387 | 28.3% |  | -202 | -33.0% |
|  | pers-yrs |  | pers-yrs | %-change |  | pers-yrs | %-change |  | pers-yrs | %-change |  | pers-yrs | %-change |
| ΔPatient Disease Burden (YLD) | -777 |  | -163 | -79.0% |  | -1,351 | 73.8% |  | -975 | 25.4% |  | -541 | -30.3% |
| - myocardial infarction | -4 |  | -1 | -82.8% |  | -8 | 77.6% |  | -6 | 29.5% |  | -3 | -35.0% |
| - stroke | -773 |  | -163 | -79.0% |  | -1,343 | 73.8% |  | -969 | 25.4% |  | -539 | -30.3% |
| ΔPatient Worktime Loss | -362 |  | -75 | -79.3% |  | -630 | 74.0% |  | -456 | 25.9% |  | -251 | -30.8% |
| - myocardial infarction | -2 |  | 0 | -82.9% |  | -3 | 77.7% |  | -2 | 29.6% |  | -1 | -35.1% |
| - stroke | -360 |  | -75 | -79.3% |  | -627 | 74.0% |  | -453 | 25.8% |  | -249 | -30.7% |
| ΔCaregiver Time Loss | -1,587 |  | -289 | -81.8% |  | -2,798 | 76.3% |  | -2,045 | 28.8% |  | -1,048 | -34.0% |
| - stroke | -1,587 |  | -289 | -81.8% |  | -2,798 | 76.3% |  | -2,045 | 28.8% |  | -1,048 | -34.0% |
| - work time | -643 |  | -142 | -77.9% |  | -1,111 | 72.8% |  | -798 | 24.1% |  | -457 | -28.9% |
| - leisure time | -944 |  | -147 | -84.4% |  | -1,687 | 78.7% |  | -1,247 | 32.0% |  | -590 | -37.5% |
|  | mn USD |  | mn USD | %-change |  | mn USD | %-change |  | mn USD | %-change |  | mn USD | %-change |
| ΔHealth Expenses |  |  |  |  |  |  |  |  |  |  |  |  |  |
| - formal hospital | -310 |  | -257 | -17.1% |  | -360 | 16.0% |  | -328 | 5.8% |  | -289 | -6.9% |
| - myocardial infarction | -293 |  | -241 | -17.8% |  | -342 | 16.7% |  | -310 | 6.0% |  | -272 | -7.2% |
| - stroke | -17 |  | -16 | -5.6% |  | -18 | 5.3% |  | -18 | 1.9% |  | -17 | -2.3% |
| Note: Own calculations. | | | | | | | | | | | | | |

| **Table C.1.5. Sensitivity Analysis: Variation in MENSINK parameters, SFA vs MUFA/PUFA, central ± 2 standard deviations (demographic, environment)** | | | | | | | | | | | | | |
| --- | --- | --- | --- | --- | --- | --- | --- | --- | --- | --- | --- | --- | --- |
|  | Policy Scenario (central parms) |  | Sensitivity Scenario 1. (low parms: -2 std. dev.) | |  | Sensitivity Scenario 2. (high parms: +2 std. dev.) | |  | Sensitivity Scenario 3.  (high SFA: + 2 std.dev.) (low MUFA/PUFA: -2 std. dev.) | |  | Sensitivity Scenario 4.  (low SFA: -2 std. dev.)  (high MUFA/PUFA: +2 std. dev.) | |
| ***Demographic (cumulative indicators)*** |  |  |  |  |  |  |  |  |  |  |  |  |  |
|  | pers-yrs |  | pers-yrs | %-change |  | pers-yrs | %-change |  | pers-yrs | %-change |  | pers-yrs | %-change |
| Δpopulation | 13,621 |  | 980 | -92.8% |  | 25,384 | 86.4% |  | 19,331 | 41.9% |  | 7,040 | -48.3% |
| - Bangkok^1^ | 886 |  | -925 | -204.4% |  | 2,537 | 186.2% |  | 2,450 | 176.4% |  | -836 | -194.3% |
| - Central region (exc Bangkok)^1^ | 7,387 |  | 2,919 | -60.5% |  | 11,626 | 57.4% |  | 7,601 | 2.9% |  | 6,949 | -5.9% |
| - North region^1^ | 2,544 |  | 248 | -90.3% |  | 4,685 | 84.1% |  | 3,531 | 38.8% |  | 1,400 | -45.0% |
| - Northeast region^1^ | 2,721 |  | 310 | -88.6% |  | 4,969 | 82.6% |  | 3,727 | 37.0% |  | 1,551 | -43.0% |
| - South region^1^ | 83 |  | -1,572 | -1989.1% |  | 1,568 | 1783.7% |  | 2,022 | 2329.2% |  | -2,024 | -2532.2% |
| - urban^1^ | 5,019 |  | -3,276 | -165.3% |  | 12,605 | 151.2% |  | 11,510 | 129.3% |  | -2,172 | -143.3% |
| - rural^1^ | 8,602 |  | 4,256 | -50.5% |  | 12,779 | 48.5% |  | 7,821 | -9.1% |  | 9,212 | 7.1% |
|  | pers-yrs |  | pers-yrs | %-change |  | pers-yrs | %-change |  | pers-yrs | %-change |  | pers-yrs | %-change |
| Δworkforce | 4,450 |  | 485 | -89.1% |  | 8,137 | 82.9% |  | 6,125 | 37.7% |  | 2,511 | -43.6% |
| - urban^1^ | 1,621 |  | -907 | -155.9% |  | 3,931 | 142.5% |  | 3,541 | 118.4% |  | -504 | -131.1% |
| - rural^1^ | 2,829 |  | 1,392 | -50.8% |  | 4,206 | 48.7% |  | 2,584 | -8.6% |  | 3,015 | 6.6% |
| ***Environment (cumulative indicator)*** |  |  |  |  |  |  |  |  |  |  |  |  |  |
|  | Mt CO_2_-eq |  | Mt CO_2_-eq | %-change |  | Mt CO_2_-eq | %-change |  | Mt CO_2_-eq | %-change |  | Mt CO_2_-eq | %-change |
| ΔGHG emissions | 7.52 |  | 7.52 | 0.0% |  | 7.52 | 0.0% |  | 7.52 | 0.0% |  | 7.52 | 0.0% |
| Note: Own calculations. ^1^ Regional population %-impacts calculated as share of projected regional totals. | | | | | | | | | | | | | |

This nutrition sensitivity analysis is focused on the nutritional transmission of the health pathway. The sales tax pathway is therefore only affected by secondary effects (delta-delta terms). Given the relatively small magnitude of the health pathway, the sales tax pathway impacts turn out to be negligible (variation of Δreal GDP of <0.01%/< USD 1mn in absolute terms). Similar reasoning explains why GHG emission impacts measured by mega-tonnes (Mt) of CO_2_-equivalents (CO_2_-eq), are negligible (variation of <0.01%/<0.001 Mt CO_2_-eq). We therefore focus on the health pathway results.

Sensitivity scenarios 1 and 2 are extreme nutrition impact scenarios with uniform changes in SFA, MUFA and PUFA elasticities to respectively lower and upper confidence bounds (Mensink et al. 2003). The results show strong variation in health pathway impacts: real GDP impacts vary by 13- USD 172mn (policy sim=USD 95mn), real household consumption impacts vary by USD 6-87mn (policy sim=USD 48mn), cholesterol biomarker impacts vary from -0.01 to -0.19 (policy sim=-0.10), saved incident cases and saved premature deaths vary, respectively, by 620-6,333 (policy sim=3,570) and by 343-3,281 (policy sim=1,861), and population impacts vary by 980-25,384 (policy sim=13,621). In general, aggregate policy indicators (excluding the Total:HDL serum cholesterol biomarker) vary by between -93% and +86% in Scenarios 1 and 2, indicating that statistical uncertainty associated with the SFA, MUFA and PUFA elasticities (Mensink et al. 2003) may lead to considerable uncertainty in simulated health pathway outcomes including nutrition, cholesterol biomarker, clinical health outcome, and economic indicators. Nonetheless, it should be noted that realizations of extreme values for all three parameters in scenarios 1 and 2 are very unlikely events (p-value ≈ 0.0016%). Moreover, it should be noted that aggregate health pathway impacts remain uniformly positive, even for the most adverse re-specification of parameters in scenario 2 (but see discussion of possible household-level impacts sign change, below).

Scenarios 3 and 4 takes SFA and MUFA/PUFA to opposite bounds. Scenario 3 increases the SFA elasticity to its upper statistical bound and MUFA/PUFA elasticities to their lower bounds (implying that the elasticities are relatively uniform and all positive), while Scenario 4 changes elasticities in opposite directions. The ‘uniform elasticity’ scenario 3 increases aggregate health pathway indicators (except health expenses) by 24-42%, while the ‘diverse elasticity’ scenario 4 reduces aggregate health pathway indicators (except health expenses) by 30-48%. The nutritional cholesterol biomarker indicator expands/contracts by 45% and 51% respectively.

The result patterns suggest that aggregate impacts are particularly sensitive to variation in the SFA parameter (e.g. compare difference between scenarios 1 and 3, to difference between scenarios 1 and 2). Hence, a four standard deviation increase in the SFA parameter increases health impacts of reduced palm oil consumption by about twice as much (or more) as a combined four standard deviation increase in both MUFA and PUFA parameters (and vice versa). This is likely to reflect the particularly high SFA contents of palm cooking oil as well as the relatively high baseline SFA intake shares (relative to PUFA/MUFA intake shares) of Thai households and their behavioural substitution responses to increased palm cooking oil prices.

Turning to household-level impacts, it is interesting to note the possibility of sign change for regional outcomes. In the lower bound scenario 1, population and real consumption impacts are negative for Bangkok and the South region. Furthermore, the rural/urban breakdown indicates a net population expansion (980 person-years) composed of an urban population reduction (-3,276 person-years) and a rural population expansion (4,256 person-years). The same pattern is observed for rural/urban workforce impacts where a net workforce expansion (485 person-years) is composed of an urban workforce reduction (-907 person-years) and a rural workforce expansion (1,392 person-years).

These results indicate that variation in nutrition parameter values (within confidence bounds) may reverse the perception that palm cooking oil consumption has adverse health consequences for all Thai population strata. Hence, observed food substitution patterns associated with reduced palm cooking oil consumption is not likely to affect the positive sign of rural health impacts, but may lead to adverse overall health outcomes for some strata of urban Thai households. Nonetheless, it should be reiterated that realization of scenario 2 extreme values for all three parameters is a very unlikely event (p-value ≈ 0.0016%).

Comparing household-level impacts across all four nutritional sensitivity scenarios, it is clear that urban outcomes, e.g. urban population impacts (-3,276 to 12,605 person-years), vary relatively strongly compared to rural outcomes (4,256 to 12,779 person-years). Interestingly, the result patterns of scenarios 3 and 4 suggests that urban impacts are most sensitive to SFA parameter variations (e.g. compare the difference between scenarios 1 and 3 with the difference between scenarios 1 and 2), while rural impacts are most sensitive to MUFA/PUFA parameter variations (e.g. compare the difference between scenarios 1 and 4 with the difference between scenarios 1 and 2).

Overall, a four standard deviation increase in the SFA parameter increases urban health impacts of reduced palm oil consumption by about ten times as much as a combined four std. dev. increase in both MUFA and PUFA parameters, while a four stdandard deviation increase in the SFA parameter increases rural health impacts by less (about 75%) than a similar increase in MUFA and PUFA parameters. It is the strong urban sensitivity pattern which explains the sensitivity of aggregate impacts to SFA parameter variation (see discussion above). The particularly strong sensitivity of urban health impacts to the SFA parameter is likely to reflect relatively high baseline SFA intake shares of urban (relative to rural) Thai households and their behavioural substitution responses to increased palm cooking oil prices.

| **Table C.2.1. Sensitivity Analysis Scenarios: Variation in workforce participation rates** | |
| --- | --- |
| Scenarios | Description |
| Policy Scenario | -50% average household consumption of (palm) cooking oil (workforce participation rate=72.3%) |
| Sensitivity Scenario 1 | -50% average household consumption of (palm) cooking oil (workforce participation rate=65%) |
| Sensitivity Scenario 2 | -50% average household consumption of (palm) cooking oil (workforce participation rate=70%) |
| Sensitivity Scenario 3 | -50% average household consumption of (palm) cooking oil (workforce participation rate=75%) |
| Sensitivity Scenario 4 | -50% average household consumption of (palm) cooking oil (workforce participation rate=80%) |
| Instrument | Product-specific sales tax on palm cooking oil |

## C.2. Sensitivity to workforce participation rates

The second set of sensitivity analyses, with a focus on workforce participation rates (p. rates), contains the first set (of two) of labour market sensitivity analyses (the second focuses on retirement age, see section C.3 below). The specification of workforce p. rates (unskilled and skilled) defines the rates of employment among both healthy and sick individuals, and thereby affects the economic impacts of both health and sales tax pathways. In order to investigate the sensitivity of our results to our baseline assumptions (uniform p. rates = 72.3%), we simulate four equally spaced sensitivity scenarios where p. rates are uniformly varied between 65%-80% (Table C.2.1). Results are presented in Tables C.2.2-C.2.4.

Notably, the results suggest that p. rates are mixed correlated with economic indicators (real GDP and private consumption: negative, real government consumption and investment: positive), positively correlated with health indicators, and negatively related to our environmental indicator. Hence, increasing p. rates lead to worsening of all main indicators including economic welfare (real GDP and private consumption) as well as nutritional, health, and environmental outcomes. The main exceptions are real government consumption and investment which expands with increasing p. rates.

| **Table C.2.2. Sensitivity Analysis: Variation in workforce participation rates (economy)** | | | | | | | | | | | | | |
| --- | --- | --- | --- | --- | --- | --- | --- | --- | --- | --- | --- | --- | --- |
|  | Policy Scenario (p. rate=72.3%) |  | Sensitivity Scenario 1. (p. rate=65%) | |  | Sensitivity Scenario 2. (p. rate=70%) | |  | Sensitivity Scenario 3. (p. rate=75%) | |  | Sensitivity Scenario 4. (p. rate=80%) | |
| ***Real GDP & sales tax (cumulative and long run indicators)*** |  |  |  |  |  |  |  |  |  |  |  |  |  |
|  | mn USD |  | mn USD | %-change |  | mn USD | %-change |  | mn USD | %-change |  | mn USD | %-change |
| Δreal GDP (cumulative) | 25,145 |  | 25,739 | 2.4% |  | 25,314 | 0.7% |  | 24,972 | -0.7% |  | 24,701 | -1.8% |
| - health pathway (cumulative) | 95 |  | 122 | 28.5% |  | 103 | 8.2% |  | 87 | -8.7% |  | 73 | -23.0% |
| - sales tax pathway (cumulative) | 25,050 |  | 25,616 | 2.3% |  | 25,211 | 0.6% |  | 24,885 | -0.7% |  | 24,628 | -1.7% |
|  | mn USD |  | mn USD | %-change |  | mn USD | %-change |  | mn USD | %-change |  | mn USD | %-change |
| Δreal GDP (cumulative) | 25,145 |  | 25,739 | 2.4% |  | 25,314 | 0.7% |  | 24,972 | -0.7% |  | 24,701 | -1.8% |
| - Private Consumption (cumulative) | -9,882 |  | -8,794 | -11.0% |  | -9,562 | -3.2% |  | -10,221 | 3.4% |  | -10,785 | 9.1% |
| - Government Consumption (cumulative) | 8,633 |  | 8,377 | -3.0% |  | 8,555 | -0.9% |  | 8,719 | 1.0% |  | 8,869 | 2.7% |
| - Investment (cumulative) | 26,394 |  | 26,156 | -0.9% |  | 26,321 | -0.3% |  | 26,474 | 0.3% |  | 26,617 | 0.8% |
| - Exports (cumulative) | 35,857 |  | 34,185 | -4.7% |  | 35,312 | -1.5% |  | 36,493 | 1.8% |  | 37,707 | 5.2% |
| - Imports (cumulative) | 35,857 |  | 34,185 | -4.7% |  | 35,312 | -1.5% |  | 36,493 | 1.8% |  | 37,707 | 5.2% |
|  | %-points |  | %-points | %-point change |  | %-points | %-point change |  | %-points | %-point change |  | %-points | %-point change |
| Δsales tax (long run, 2035) | 53.5% |  | 53.9% | 0.33% |  | 53.6% | 0.09% |  | 53.4% | -0.09% |  | 53.3% | -0.24% |
| Δinvestment price index (long run, 2035) | -0.8% |  | -0.8% | 0.01% |  | -0.8% | 0.00% |  | -0.8% | 0.00% |  | -0.9% | -0.01% |
| Δreal exchange rate (long run, 2035) | -0.9% |  | -0.8% | 0.01% |  | -0.9% | 0.00% |  | -0.9% | 0.00% |  | -0.9% | -0.01% |
| ***Real Consumption (cumulative indicators)*** |  |  |  |  |  |  |  |  |  |  |  |  |  |
|  | mn USD |  | mn USD | %-change |  | mn USD | %-change |  | mn USD | %-change |  | mn USD | %-change |
| Δreal Household Consumption | -9,882 |  | -8,794 | -11.0% |  | -9,562 | -3.2% |  | -10,221 | 3.4% |  | -10,785 | 9.1% |
| - sales tax pathway | -9,930 |  | -8,855 | -10.8% |  | -9,614 | -3.2% |  | -10,265 | 3.4% |  | -10,823 | 9.0% |
| - Bangkok^1^ | -362 |  | -261 | -27.9% |  | -332 | -8.1% |  | -392 | 8.5% |  | -443 | 22.5% |
| - Central region (exc Bangkok)^1^ | -3,336 |  | -3,130 | -6.2% |  | -3,278 | -1.7% |  | -3,393 | 1.7% |  | -3,481 | 4.3% |
| - North region^1^ | -935 |  | -803 | -14.1% |  | -896 | -4.1% |  | -976 | 4.4% |  | -1,045 | 11.8% |
| - Northeast region^1^ | -982 |  | -858 | -12.6% |  | -947 | -3.6% |  | -1,019 | 3.7% |  | -1,077 | 9.6% |
| - South region^1^ | -4,315 |  | -3,803 | -11.9% |  | -4,160 | -3.6% |  | -4,484 | 3.9% |  | -4,777 | 10.7% |
| - health pathway | 48 |  | 61 | 27.0% |  | 52 | 7.8% |  | 44 | -8.3% |  | 37 | -22.1% |
| - Bangkok^1^ | 8 |  | 11 | 36.9% |  | 9 | 10.7% |  | 7 | -11.3% |  | 6 | -29.9% |
| - Central region (exc Bangkok)^1^ | 19 |  | 23 | 20.3% |  | 20 | 5.9% |  | 18 | -6.3% |  | 16 | -16.8% |
| - North region^1^ | 7 |  | 9 | 27.2% |  | 7 | 7.9% |  | 6 | -8.3% |  | 5 | -22.2% |
| - Northeast region^1^ | 9 |  | 11 | 26.1% |  | 10 | 7.6% |  | 8 | -8.0% |  | 7 | -21.4% |
| - South region^1^ | 5 |  | 7 | 37.1% |  | 6 | 10.8% |  | 5 | -11.4% |  | 4 | -30.3% |
| Note: Own calculations. ^1^ Regional consumption %-impacts calculated as share of projected regional totals. | | | | | | | | | | | | | |

| **Table C.2.3. Sensitivity Analysis: Variation in workforce participation rates (nutrition, biomarker, health)** | | | | | | | | | | | | | |
| --- | --- | --- | --- | --- | --- | --- | --- | --- | --- | --- | --- | --- | --- |
|  | Policy Scenario (p. rate=72.3%) |  | Sensitivity Scenario 1. (p. rate=65%) | |  | Sensitivity Scenario 2. (p. rate=70%) | |  | Sensitivity Scenario 3. (p. rate=75%) | |  | Sensitivity Scenario 4. (p. rate=80%) | |
| ***Nutrition (long run indicators)*** |  |  |  |  |  |  |  |  |  |  |  |  |  |
|  | %-points |  | %-points | %-change |  | %-points | %-change |  | %-points | %-change |  | %-points | %-change |
| ΔSFA energy intake share (long run, 2035) | -0.322% |  | -0.300% | -6.8% |  | -0.316% | -2.0% |  | -0.329% | 2.1% |  | -0.340% | 5.4% |
| ΔMUFA energy intake share (long run, 2035) | -0.164% |  | -0.139% | -15.1% |  | -0.157% | -4.3% |  | -0.171% | 4.6% |  | -0.184% | 12.0% |
| ΔPUFA energy intake share (long run, 2035) | 0.298% |  | 0.339% | 13.9% |  | 0.309% | 4.0% |  | 0.285% | -4.2% |  | 0.264% | -11.1% |
| ***Biomarker (cumulative indicators)*** |  |  |  |  |  |  |  |  |  |  |  |  |  |
|  | cum. chg. |  | cum. chg. | %-change |  | cum. chg. | %-change |  | cum. chg. | %-change |  | cum. chg. | %-change |
| ΔTotal-to-HDL cholesterol ratio | -0.101 |  | -0.131 | 30.2% |  | -0.110 | 8.8% |  | -0.092 | -9.2% |  | -0.076 | -24.6% |
| ***Health (cumulative indicators)*** |  |  |  |  |  |  |  |  |  |  |  |  |  |
|  | cases |  | cases | %-change |  | cases | %-change |  | cases | %-change |  | cases | %-change |
| ΔPatient Incident Cases | -3,570 |  | -4,537 | 27.1% |  | -3,850 | 7.8% |  | -3,275 | -8.3% |  | -2,786 | -22.0% |
| - myocardial infarction | -2,704 |  | -3,442 | 27.3% |  | -2,918 | 7.9% |  | -2,479 | -8.3% |  | -2,107 | -22.1% |
| - stroke | -866 |  | -1,095 | 26.4% |  | -932 | 7.7% |  | -796 | -8.1% |  | -679 | -21.6% |
| ΔPatient premature deaths | -1,861 |  | -2,356 | 26.6% |  | -2,005 | 7.7% |  | -1,710 | -8.1% |  | -1,459 | -21.6% |
| - myocardial infarction | -1,560 |  | -1,980 | 26.9% |  | -1,682 | 7.8% |  | -1,432 | -8.2% |  | -1,220 | -21.8% |
| - stroke | -301 |  | -376 | 24.9% |  | -323 | 7.3% |  | -278 | -7.7% |  | -239 | -20.7% |
|  | pers-yrs |  | pers-yrs | %-change |  | pers-yrs | %-change |  | pers-yrs | %-change |  | pers-yrs | %-change |
| ΔPatient Disease Burden (YLD) | -777 |  | -970 | 24.8% |  | -833 | 7.2% |  | -718 | -7.6% |  | -619 | -20.3% |
| - myocardial infarction | -4 |  | -6 | 27.3% |  | -5 | 7.9% |  | -4 | -8.3% |  | -3 | -22.1% |
| - stroke | -773 |  | -964 | 24.8% |  | -829 | 7.2% |  | -714 | -7.6% |  | -616 | -20.3% |
| ΔPatient Worktime Loss | -362 |  | -405 | 12.0% |  | -375 | 3.7% |  | -347 | -4.2% |  | -320 | -11.7% |
| - myocardial infarction | -2 |  | -2 | 14.3% |  | -2 | 4.4% |  | -2 | -4.9% |  | -2 | -13.7% |
| - stroke | -360 |  | -403 | 11.9% |  | -373 | 3.7% |  | -345 | -4.2% |  | -318 | -11.7% |
| ΔCaregiver Time Loss | -1,587 |  | -2,002 | 26.1% |  | -1,708 | 7.6% |  | -1,460 | -8.0% |  | -1,248 | -21.4% |
| - stroke | -1,587 |  | -2,002 | 26.1% |  | -1,708 | 7.6% |  | -1,460 | -8.0% |  | -1,248 | -21.4% |
| - work time | -643 |  | -799 | 24.2% |  | -688 | 7.1% |  | -595 | -7.5% |  | -515 | -19.9% |
| - leisure time | -944 |  | -1,203 | 27.4% |  | -1,019 | 8.0% |  | -865 | -8.4% |  | -732 | -22.4% |
|  | mn USD |  | mn USD | %-change |  | mn USD | %-change |  | mn USD | %-change |  | mn USD | %-change |
| ΔHealth Expenses |  |  |  |  |  |  |  |  |  |  |  |  |  |
| - formal hospital | -310 |  | -332 | 6.9% |  | -316 | 2.0% |  | -304 | -2.1% |  | -293 | -5.5% |
| - myocardial infarction | -293 |  | -314 | 7.1% |  | -299 | 2.1% |  | -286 | -2.2% |  | -276 | -5.7% |
| - stroke | -17 |  | -18 | 3.0% |  | -18 | 0.9% |  | -17 | -0.9% |  | -17 | -2.4% |
| Note: Own calculations. | | | | | | | | | | | | | |

| **Table C.2.4. Sensitivity Analysis: Variation in workforce participation rates (demographic, environment)** | | | | | | | | | | | | | |
| --- | --- | --- | --- | --- | --- | --- | --- | --- | --- | --- | --- | --- | --- |
|  | Policy Scenario (p. rate=72.3%) |  | Sensitivity Scenario 1. (p. rate=65%) | |  | Sensitivity Scenario 2. (p. rate=70%) | |  | Sensitivity Scenario 3. (p. rate=75%) | |  | Sensitivity Scenario 4. (p. rate=80%) | |
| ***Demographic (cumulative indicators)*** |  |  |  |  |  |  |  |  |  |  |  |  |  |
|  | pers-yrs |  | pers-yrs | %-change |  | pers-yrs | %-change |  | pers-yrs | %-change |  | pers-yrs | %-change |
| Δpopulation | 13,621 |  | 17,469 | 28.2% |  | 14,742 | 8.2% |  | 12,435 | -8.7% |  | 10,455 | -23.2% |
| - Bangkok^1^ | 886 |  | 1,580 | 78.3% |  | 1,087 | 22.6% |  | 675 | -23.8% |  | 326 | -63.2% |
| - Central region (exc Bangkok)^1^ | 7,387 |  | 8,655 | 17.2% |  | 7,756 | 5.0% |  | 6,995 | -5.3% |  | 6,340 | -14.2% |
| - North region^1^ | 2,544 |  | 3,242 | 27.4% |  | 2,748 | 8.0% |  | 2,329 | -8.5% |  | 1,968 | -22.6% |
| - Northeast region^1^ | 2,721 |  | 3,483 | 28.0% |  | 2,944 | 8.2% |  | 2,484 | -8.7% |  | 2,086 | -23.3% |
| - South region^1^ | 83 |  | 509 | 511.6% |  | 207 | 148.8% |  | -48 | -157.3% |  | -266 | -419.5% |
| - urban^1^ | 5,019 |  | 7,494 | 49.3% |  | 5,739 | 14.4% |  | 4,257 | -15.2% |  | 2,986 | -40.5% |
| - rural^1^ | 8,602 |  | 9,975 | 16.0% |  | 9,002 | 4.7% |  | 8,178 | -4.9% |  | 7,469 | -13.2% |
|  | pers-yrs |  | pers-yrs | %-change |  | pers-yrs | %-change |  | pers-yrs | %-change |  | pers-yrs | %-change |
| Δworkforce | 4,450 |  | 5,146 | 15.6% |  | 4,664 | 4.8% |  | 4,211 | -5.4% |  | 3,781 | -15.0% |
| - urban^1^ | 1,621 |  | 2,152 | 32.8% |  | 1,785 | 10.1% |  | 1,437 | -11.4% |  | 1,101 | -32.1% |
| - rural^1^ | 2,829 |  | 2,994 | 5.8% |  | 2,878 | 1.8% |  | 2,774 | -1.9% |  | 2,680 | -5.3% |
| ***Environment (cumulative indicator)*** |  |  |  |  |  |  |  |  |  |  |  |  |  |
|  | Mt CO_2_-eq |  | Mt CO_2_-eq | %-change |  | Mt CO_2_-eq | %-change |  | Mt CO_2_-eq | %-change |  | Mt CO_2_-eq | %-change |
| ΔGHG emissions | 7.52 |  | 7.21 | -4.1% |  | 7.45 | -0.9% |  | 7.81 | 3.8% |  | 7.91 | 5.2% |
| Note: Own calculations. ^1^ Regional population %-impacts calculated as share of projected regional totals. | | | | | | | | | | | | | |

In terms of the health pathway, reduced p. rates lower labour factor ratios (vis-à-vis land and capital) and increases relative labour wages, and thereby increases the economic reward for improved health among working age groups. Aggregate real GDP impacts vary by USD 73-122mn (policy sim=USD 95mn) or from -23% to +29% (Table C.2.2), while real consumption varies by USD 37-61mn (policy sim=USD 48mn) or from -22% to +27% (Table C.2.2). The health pathway impacts include no major changes in relative prices. Changes in value added generation is therefore mirrored by proportional changes in real consumption.

In terms of the sales tax pathway, our policy scenario analysis showed that palm cooking oil sales taxes give rise to long run real GDP expansion. The reduced p. rates in Scenarios 1-2 lower labour intensity rates, in general, and for investment products and government services, in particular (the latter production sectors are characterized by relatively high labour factor intensities). The increased marginal returns to labour implies that reallocation of labour is less costly, and this improves real GDP outcomes. Furthermore, the small twist in relative consumer vs. investment prices leads to improved private consumption outcomes and slightly worsened investment and government consumption outcomes. Aggregate real GDP impacts vary by 24.6-25.6bn USD (policy sim=25.0bn USD) or from -1.7% to +2.3%, while real consumption impacts vary from -25.6bn USD to -26.3bn USD (policy sim=-26.0bn USD) or from -1.3% to +1.3%. Hence, private consumption accounts for the main adjustment, both in absolute and relative terms, due to the revenue-neutral government budget closure and the twist in relative investment vs. consumption prices.

The negative correlation between p. rates and health and nutrition indicators is more interesting, with indicator impacts varying from -22% to +27% (Table C.2.3). The Total-to-HDL cholesterol biomarker varies between -0.131 to -0.076 (policy sim=-0.101), driven by negatively correlated variation in PUFA energy intake shares. Interestingly, variation in SFA energy intake shares is also negatively correlated with p. rates, but, similar to the policy scenario analysis, the PUFA effect dominates.

Taking a closer look at the impacts on nutritional composition, it is apparent that e.g. demand expansion through reduced p. rates leads Thai consumers to increase energy intake shares of both SFA, MUFA and PUFA. Nonetheless, cholesterol biomarker impacts are dominated by strong variation in PUFA intakes, leading the average long run cholesterol biomarker to vary relatively strongly between -0.131 to -0.076 (policy sim=-0.101). These results underline that Thai consumers, in the aggregate, are likely to respond to increased relative palm oil prices through healthy demand expansion of PUFA-dense food groups, and that this impact is enhanced by a tighter labour market.

Finally, we observe a positive correlation between p. rates and our environmental indicator of GHG emissions. This is a direct consequence of the fact that oil palm production is relatively land intensive, and that land conversion from oil palm to other agricultural use, according to our data, increases emissions. E.g. a reduction in labour supplies (low p. rate) reduces labour intensities and land returns in more labour-intensive agricultural sectors, and this reduces economic incentives for other agricultural sectors to convert land from oil palm production following the introduction of palm oil taxes. Overall, GHG emission impacts vary by 7.21-7.91 Mt CO_2_-eq or from -4.1% to +5.2%.

| **Table C.3.1. Sensitivity Analysis Scenarios: Variation in workforce retirement age parameters** | |
| --- | --- |
| Scenarios | Description |
| Policy Scenario | -50% average household consumption of (palm) cooking oil (workforce retirement age=64) |
| Sensitivity Scenario 1 | -50% average household consumption of (palm) cooking oil (workforce retirement age=59) |
| Sensitivity Scenario 2 | -50% average household consumption of (palm) cooking oil (workforce retirement age=69) |
| Instrument | Product-specific sales tax on (palm) cooking oil |

## C.3. Sensitivity to retirement age

The third set of sensitivity analyses, with a focus on retirement age (r. age), contains the second set of labour market sensitivity analyses (the first focuses on p. rates, see section C.2 above). The specification of r. age defines, together with p. rates, the supply of labour among healthy and sick individuals, and thereby affects the economic impacts of both sales tax and health pathways. In order to investigate the sensitivity of our results to our baseline assumption (r. age = 64), we simulate two equally spaced sensitivity scenarios where r. age is varied between 59 and 69 years (Table C.3.1). Results are presented in Tables C.3.2-C.3.4.

As noted, above, changes in r. age and p. rates work together to determine labour supplies. Hence, it is not surprising that sensitivity results of r. age in this section mirror the sensitivity results of p. rates in the previous section C.2. Again, we find that r. age is mixed correlated with aggregate economic indicators (real GDP: negative, real consumption: positive), negatively correlated with aggregate health indicators (but note discussion of positively correlated patient work time losses), and positively related to our environmental indicator increasing r. age lead to (limited) worsening of all main indicators including economic welfare (real GDP and private consumption) as well as nutrition, health and environmental outcomes. The main exceptions are, again, real government consumption and investment which tend to expand with increasing r. age.

| **Table C.3.2. Sensitivity Analysis: Variation in workforce retirement age parameters (economy)** | | | | | | | |
| --- | --- | --- | --- | --- | --- | --- | --- |
|  | Policy Scenario  (r. age=64) |  | Sensitivity Scenario 1.  (r. age=59) | |  | Sensitivity Scenario 2.  (r. age=69) | |
| ***Real GDP & sales tax (cumulative and long run indicators)*** |  |  |  |  |  |  |  |
|  | mn USD |  | mn USD | %-change |  | mn USD | %-change |
| Δreal GDP (cumulative) | 25,145 |  | 25,780 | 2.5% |  | 24,753 | -1.6% |
| - health pathway (cumulative) | 95 |  | 111 | 16.9% |  | 87 | -9.2% |
| - sales tax pathway (cumulative) | 25,050 |  | 25,669 | 2.5% |  | 24,667 | -1.5% |
|  | mn USD |  | mn USD | %-change |  | mn USD | %-change |
| Δreal GDP (cumulative) | 25,145 |  | 25,780 | 2.5% |  | 24,753 | -1.6% |
| - Private Consumption (cumulative) | -9,882 |  | -8,764 | -11.3% |  | -10,592 | 7.2% |
| - Government Consumption (cumulative) | 8,633 |  | 8,375 | -3.0% |  | 8,810 | 2.0% |
| - Investment (cumulative) | 26,394 |  | 26,169 | -0.9% |  | 26,536 | 0.5% |
| - Exports (cumulative) | 35,857 |  | 34,267 | -4.4% |  | 37,246 | 3.9% |
| - Imports (cumulative) | 35,857 |  | 34,267 | -4.4% |  | 37,246 | 3.9% |
|  | %-points |  | %-points | %-point change |  | %-points | %-point change |
| Δsales tax (long run, 2035) | 53.5% |  | 53.9% | 0.39% |  | 53.3% | -0.23% |
| Δinvestment price index (long run, 2035) | -0.8% |  | -0.8% | 0.01% |  | -0.9% | -0.01% |
| Δreal exchange rate (long run, 2035) | -0.9% |  | -0.8% | 0.01% |  | -0.9% | -0.01% |
| ***Real Consumption (cumulative indicators)*** |  |  |  |  |  |  |  |
|  | mn USD |  | mn USD | %-change |  | mn USD | %-change |
| Δreal Household Consumption | -9,882 |  | -8,764 | -11.3% |  | -10,592 | 7.2% |
| - sales tax pathway | -9,930 |  | -8,819 | -11.2% |  | -10,637 | 7.1% |
| - Bangkok^1^ | -362 |  | -271 | -25.0% |  | -419 | 15.8% |
| - Central region (exc Bangkok)^1^ | -3,336 |  | -3,156 | -5.4% |  | -3,418 | 2.5% |
| - North region^1^ | -935 |  | -746 | -20.2% |  | -1,056 | 13.0% |
| - Northeast region^1^ | -982 |  | -827 | -15.8% |  | -1,083 | 10.3% |
| - South region^1^ | -4,315 |  | -3,819 | -11.5% |  | -4,660 | 8.0% |
| - health pathway | 48 |  | 55 | 13.8% |  | 44 | -7.5% |
| - Bangkok^1^ | 8 |  | 10 | 25.7% |  | 6 | -17.3% |
| - Central region (exc Bangkok)^1^ | 19 |  | 20 | 5.6% |  | 19 | -0.8% |
| - North region^1^ | 7 |  | 8 | 10.5% |  | 6 | -5.3% |
| - Northeast region^1^ | 9 |  | 10 | 13.3% |  | 8 | -6.3% |
| - South region^1^ | 5 |  | 7 | 29.9% |  | 4 | -21.4% |
| Note: Own calculations. ^1^ Regional consumption %-impacts calculated as share of projected regional totals. | | | | | | | |

| **Table C.3.3. Sensitivity Analysis: Variation in workforce retirement age parameters (nutrition, biomarker, health)** | | | | | | | |
| --- | --- | --- | --- | --- | --- | --- | --- |
|  | Policy Scenario (r. age=64) |  | Sensitivity Scenario 1.  (r. age=59) | |  | Sensitivity Scenario 2.  (r. age=69) | |
| ***Nutrition (long run indicators)*** |  |  |  |  |  |  |  |
|  | %-points |  | %-points | %-change |  | %-points | %-change |
| ΔSFA energy intake share (long run, 2035) | -0.322% |  | -0.296% | -8.0% |  | -0.338% | 5.1% |
| ΔMUFA energy intake share (long run, 2035) | -0.164% |  | -0.135% | -17.5% |  | -0.182% | 11.1% |
| ΔPUFA energy intake share (long run, 2035) | 0.298% |  | 0.345% | 16.1% |  | 0.267% | -10.3% |
| ***Biomarker (cumulative indicators)*** |  |  |  |  |  |  |  |
|  | cum. chg. |  | cum. chg. | %-change |  | cum. chg. | %-change |
| ΔTotal-to-HDL cholesterol ratio | -0.101 |  | -0.131 | 29.6% |  | -0.082 | -18.5% |
| ***Health (cumulative indicators)*** |  |  |  |  |  |  |  |
|  | cases |  | cases | %-change |  | cases | %-change |
| ΔPatient Incident Cases | -3,570 |  | -4,532 | 26.9% |  | -2,969 | -16.8% |
| - myocardial infarction | -2,704 |  | -3,438 | 27.1% |  | -2,247 | -16.9% |
| - stroke | -866 |  | -1,094 | 26.3% |  | -722 | -16.6% |
| ΔPatient premature deaths | -1,861 |  | -2,356 | 26.6% |  | -1,551 | -16.7% |
| - myocardial infarction | -1,560 |  | -1,980 | 26.9% |  | -1,298 | -16.8% |
| - stroke | -301 |  | -376 | 25.0% |  | -253 | -16.0% |
|  | pers-yrs |  | pers-yrs | %-change |  | pers-yrs | %-change |
| ΔPatient Disease Burden (YLD) | -777 |  | -969 | 24.7% |  | -656 | -15.6% |
| - myocardial infarction | -4 |  | -6 | 27.1% |  | -4 | -16.9% |
| - stroke | -773 |  | -964 | 24.7% |  | -652 | -15.6% |
| ΔPatient Worktime Loss | -362 |  | -313 | -13.5% |  | -405 | 11.8% |
| - myocardial infarction | -2 |  | -2 | -4.5% |  | -2 | 3.3% |
| - stroke | -360 |  | -311 | -13.6% |  | -403 | 11.8% |
| ΔCaregiver Time Loss | -1,587 |  | -2,001 | 26.1% |  | -1,327 | -16.4% |
| - stroke | -1,587 |  | -2,001 | 26.1% |  | -1,327 | -16.4% |
| - work time | -643 |  | -798 | 24.2% |  | -545 | -15.2% |
| - leisure time | -944 |  | -1,202 | 27.4% |  | -782 | -17.2% |
|  | mn USD |  | mn USD | %-change |  | mn USD | %-change |
| ΔHealth Expenses |  |  |  |  |  |  |  |
| - formal hospital | -310 |  | -332 | 6.9% |  | -297 | -4.2% |
| - myocardial infarction | -293 |  | -314 | 7.1% |  | -280 | -4.4% |
| - stroke | -17 |  | -18 | 3.0% |  | -17 | -1.8% |
| Note: Own calculations. | | | | | | | |

| **Table C.3.4. Sensitivity Analysis: Variation in workforce retirement age parameters (demographic, environment)** | | | | | | | |
| --- | --- | --- | --- | --- | --- | --- | --- |
|  | Policy Scenario (r. age=64) |  | Sensitivity Scenario 1.  (r. age=59) | |  | Sensitivity Scenario 2.  (r. age=69) | |
| ***Demographic (cumulative indicators)*** |  |  |  |  |  |  |  |
|  | pers-yrs |  | pers-yrs | %-change |  | pers-yrs | %-change |
| Δpopulation | 13,621 |  | 17,182 | 26.1% |  | 11,401 | -16.3% |
| - Bangkok^1^ | 886 |  | 1,529 | 72.5% |  | 491 | -44.6% |
| - Central region (exc Bangkok)^1^ | 7,387 |  | 8,562 | 15.9% |  | 6,651 | -10.0% |
| - North region^1^ | 2,544 |  | 3,189 | 25.4% |  | 2,142 | -15.8% |
| - Northeast region^1^ | 2,721 |  | 3,424 | 25.9% |  | 2,279 | -16.2% |
| - South region^1^ | 83 |  | 477 | 472.8% |  | -161 | -294.0% |
| - urban^1^ | 5,019 |  | 7,316 | 45.8% |  | 3,588 | -28.5% |
| - rural^1^ | 8,602 |  | 9,866 | 14.7% |  | 7,813 | -9.2% |
|  | pers-yrs |  | pers-yrs | %-change |  | pers-yrs | %-change |
| Δworkforce | 4,450 |  | 3,987 | -10.4% |  | 4,948 | 11.2% |
| - urban^1^ | 1,621 |  | 1,637 | 1.0% |  | 1,571 | -3.1% |
| - rural^1^ | 2,829 |  | 2,351 | -16.9% |  | 3,377 | 19.4% |
| ***Environment (cumulative indicator)*** |  |  |  |  |  |  |  |
|  | Mt CO_2_-eq |  | Mt CO_2_-eq | %-change |  | Mt CO_2_-eq | %-change |
| ΔGHG emissions | 7.52 |  | 7.25 | -3.7% |  | 7.87 | 4.7% |
| Note: Own calculations. ^1^ Regional population %-impacts calculated as share of projected regional totals. | | | | | | | |

As noted above, r. age sensitivity results are, qualitatively, similar to p. rate sensitivity results, and explanations for the results are also generally the same, but magnitudes differ.

First, we look at economic impacts. In terms of the health pathway, a reduction in r. age (similar to the reduced p. rate) lowers labour factor ratios and increases relative labour wages, and thereby increases the economic reward for improved health among working age groups. Aggregate real GDP impacts vary by USD 87-111mn (policy sim=USD 95mn) or from -9% to +17%, while real consumption impacts vary by USD 44-55mn (policy sim=USD 48mn) or from -8% to +14% (Table C.3.2). Again, the sensitivity health pathway impacts include no major changes in relative prices, and changes in value added generation is therefore mirrored by proportional changes in real private consumption.

In terms of the sales tax pathway, we again find that, due to the labour intensity of non-investment goods, reduced r. age enhances the palm oil tax-driven relative price twist in favour of reduced relative investment prices and increased economic growth, and thereby establishes the observed negative/positive correlations with real GDP and real consumption outcomes. Aggregate real GDP impacts vary by 24.8-25.8bn USD (policy sim=25.1bn USD) or from -1.6% to +2.5%, while real consumption impacts vary from -8.8bn USD to -10.6bn USD (policy sim=-9.9bn USD) or from -11.3% to +7.2%. As noted above, real private consumption again accounts for the main adjustment both in absolute and relative terms.

The r. age sensitivity results closely resemble the p. rate sensitivity results, both in terms of sign and size. For the health pathway, individual reductions of p. rates/r. age from 72.3%/64 years to 65%/59 years raises aggregate real GDP impacts to USD 111-122mn or +17% to +29%, while individual increases to 80%/69 years lowers aggregate real GDP impacts to USD 73-87mn or -9% to -23%. In terms of the sales tax pathway, individual reductions of p. rates/r. age from 72.3%/64 years to 65%/59 years raises aggregate real GDP impacts by USD 25.6-25.7bn or +2.3% to +2.5%, while individual increases to 80%/69 years lowers aggregate real GDP impacts by USD 24.6-24.7bn or -1.5% to -1.7%. Sensitivity results are also similar for aggregate consumption impacts, and for the individual health and sales tax pathways. Hence, for all practical purposes, the two types of labour market sensitivity analyses produce the same economic sensitivity results.

The r. age sensitivity results for health and nutrition indicators are also, generally, the same as the p. rate sensitivity results, both qualitatively and magnitude-wise. Individual reductions of p. rates/r. age from 72.3%/64 years to 65%/59 years yields almost identical results, leading to further reductions in the cholesterol biomarker to -0.131/-0.131 (policy sim=-0.101), further reductions in patient incident cases to -4,537/-4,532 (policy sim=-3,570 cases), and further reductions in premature deaths to -2,357/-2,356 (policy sim=-1,861 deaths). Increases of p. rates and r. age from 72.3%/64 years to 80%/69 years also yield qualitatively similar results. Overall, the r. age sensitivity analysis causes health and nutrition indicators to vary from -17% to +27%, which is close to the p. rate sensitivity results where these indicators vary from -22% to +27% (see section C.2 above).

All health and nutrition indicators are affected similarly by the r. age and p. rate labour market sensitivity scenarios with one exception: patient worktime losses, which was negatively correlated with p. rates, turns out to be positively correlated with r. age. The reason for this result is that variation in r. age affects large groups of workers near (or beyond) retirement, and these are also the (near-)working age groups with the highest risks of cardiovascular disease. Specifically, a reduction in p. rates from 72.3% to 65% raises the reduction in patient worktime losses to -405 patient-years (policy sim=-362 person-years), while a reduction in r. age lowers the reduction in patient worktime losses to -313 patient-years (policy sim=-362 person-years). The variations are of similar magnitude but with opposite sign (±12-14%). ^[[4]](#footnote-4)^

Finally, we note that our environmental indicator is also affected similarly by our two r. age and p. rate labour market sensitivity scenarios. A reduction in p. rates from 72.3% to 65% limits the increase in GHG emissions to +7.21 Mt CO_2_-eq, while a reduction in r. age limits the increase to +7.25 Mt (policy sim=+7.52 Mt). The variations are again almost equivalent in sign and magnitude. Increases of p. rates and r. age from 72.3%/64 years to 80%/69 years also yield qualitatively similar results. Overall, the r. age sensitivity analysis causes our environmental indicator to vary from -4% to +5%, which is similar to the p. rate sensitivity results where these indicators also vary from -4% to +5% (see section C.2 above).

## C.4. Sensitivity Analyses of palm oil own-price elasticities in household demand

The three following sensitivity analyses (sections C.4.1-C.4.3) investigates the sensitivity of results to the palm cooking oil-related elasticities underlying our AIDS demand system: (1) own-price elasticities for palm cooking oil demand (section C.4.1), (2) cross-price elasticities between palm cooking oil and other edible oils (section C.4.2), and (3) income demand elasticities for palm cooking oil (section C.4.3). Since the AIDS demand system parameters are composites of elasticities and household-specific demand shares, and since the AIDS demand parameters have to satisfy a strict set of regularity conditions, it is not possible to fix a set of elasticities without affecting the rest of the system. Hence, for each set of sensitivity analyses, we (1) fixed the relevant household-specific elasticities and proceeded to rebalance the full set of AIDS demand parameters, (2) re-calibrated our model using the new AIDS demand system specification, and (3) implemented the policy scenario of a 50% palm cooking oil energy reduction to investigate how economic, nutritional, health, demographic and environmental outcomes vary with palm oil-specific elasticities (with the caveat that other changes to the AIDS demand system may affect outcomes beyond the change in palm oil-related elasticities).

| **Table C.4.1.1. Sensitivity Analysis Scenario: palm oil own-price elasticities** | |
| --- | --- |
| Scenarios | Description |
| Policy Scenario | -50% average household consumption of (palm) cooking oil + palm oil own-price elasticity ≈ -0.94-0.95 |
| Sensitivity Scenario 1 | -50% average household consumption of (palm) cooking oil + palm oil own-price elasticity = -0.75 |
| Sensitivity Scenario 2 | -50% average household consumption of (palm) cooking oil + palm oil own-price elasticity = -0.85 |
| Sensitivity Scenario 3 | -50% average household consumption of (palm) cooking oil + palm oil own-price elasticity = -0.95 |
| Sensitivity Scenario 4 | -50% average household consumption of (palm) cooking oil + palm oil own-price elasticity = -1.05 |
| Sensitivity Scenario 5 | -50% average household consumption of (palm) cooking oil + palm oil own-price elasticity = -1.15 |
| Instrument | sales tax on palm cooking oil |

### C.4.1. Sensitivity Analyses of palm oil own-price elasticities in household demand

Sensitivity scenarios 1-5 simulate equidistant variation in palm cooking oil own-price elasticities over the range -0.75 to -1.15. The range was chosen to study a symmetric range around baseline elasticities (0.94-0.95). Scenario specifications are included in Table C.4.1.1. Results are presented in Tables C.4.1.2-C.4.1.4, and they show fairly strong variation in health pathway impacts: real GDP impacts vary by USD 58-145mn (policy sim=USD 95mn), real household consumption impacts vary by USD 29-73mn (policy sim=USD 48mn), cholesterol biomarker impacts vary from -0.06 to -0.16 (policy sim=-0.10), saved incident cases and saved premature deaths vary, respectively, by 2,184-5,427 (policy sim=3,570) and by 1,145-2,822 (policy sim=1,861), and population impacts vary by 7,918-21,248 (policy sim=13,621).

In general, aggregate health pathway indicators vary quite strongly in relative terms: macroeconomic impacts vary between -39% and +53%; nutritional impacts between -23% and +31%, biomarker impacts between -43% and +58%, health impacts between -40% and +53%, and population impacts between -42% and +56%. Finally, it can be noticed that aggregate health pathway indicator impacts are mixed correlated with palm cooking oil own-price elasticities: macroeconomic, nutritional, and demographic indicator impact correlations are positive, while cholesterol biomarker and health outcomes correlations are negative (indicating that larger (more negative) elasticities reduce beneficial health pathway outcomes across the board, i.e. lead to smaller economic gains, smaller reductions in cholesterol ratios and incident cases, save fewer lives, and lower population gains).

Results for the sales tax pathway generally show similar strong variation: The required sales tax rate to achieve the policy target vary by 46.3%-61.7% (policy sim=53.5%), real GDP impacts vary by 20.4-31.4bn USD (policy sim=USD 25.1bn), and real private consumption impacts vary by USD -7.0bn to USD -14.6bn (policy sim=USD -9.9mn).

Final demand impacts for the combined health and sales tax pathways are dominated by variation in sales tax pathway impacts, and magnitudes vary moderately: real GDP impacts vary by USD 20.4-31.6bn (policy sim=USD 25.1bn), real private consumption impacts vary by USD -7.0bn to USD -14.5bn (policy sim=USD -9.9bn), real government consumption impacts vary by USD 6.6-11.6bn (policy sim=USD 8.6bn), relative investment price impacts vary by -0.6 to -1.2%-points (policy sim=-0.9%-points), real investment impacts vary by USD 20.8-34.5bn (policy sim=USD 26.4bn), and real trade aggregate impacts vary by USD 29.6-44.5bn (policy sim=USD 35.9bn).

Aggregate real GDP and final demand impacts, generally, vary between ±30% (except for private and government consumption which vary by +46% and +34%, respectively, in the extreme scenario 1 with an own-price elasticity of -0.75), and they are, generally, positively correlated with the palm cooking oil own-price elasticity. The main exception is private consumption which is negatively correlated with real GDP impacts due to positively correlated relative consumer price impacts (or negatively correlated relative investment price impacts).

LUC-related GHG emissions impacts represent an exception to the more general rule of highly sensitive and positively correlated indicator impacts. Emissions vary minimally by 7.51-7.57 Mt CO_2_-eq (policy sim=7.52 Mt) or -0.1% to +0.7%, and correlations with palm cooking oil own price elasticities switch sign around an elasticity value of one: positive below one and negative above one.

Overall, our results turn out to be somewhat sensitive to the specification of palm cooking oil own-price elasticities. The sales tax rates, required to achieve our policy target, vary noticeably, indicating that smaller absolute elasticities (less negative) may somewhat reduce the potency of our sales tax instrument. Nonetheless, most macroeconomic indicators vary by ±30% and aggregate nutrition, health and demographic indicators by ±60% at most, and we observe no sign changes. In that sense, our results turn out to be fairly robust to variation in palm cooking oil own-price elasticities.

| **Table C.4.1.2. Sensitivity to palm oil own price elasticity of demand (50% reduction in palm oil consumption): Cumulative impact indicators for 2016-35 (economic)** | | | | | | | | | | | | | | | | |
| --- | --- | --- | --- | --- | --- | --- | --- | --- | --- | --- | --- | --- | --- | --- | --- | --- |
|  | Policy Scenario (elas ≈ -0.94-0.95) |  | Sensitivity Scenario 1. (elas = -0.75) | |  | Sensitivity Scenario 2. (elas = -0.85) | |  | Sensitivity Scenario 3. (elas = -0.95) | |  | Sensitivity Scenario 4. (elas = -1.05) | |  | Sensitivity Scenario 5. (elas = -1.15) | |
| ***Real GDP & sales tax & price indices***  ***(cumulative and long run indicators)*** | | | | | | | | | | | | | | | | |
|  | mn USD |  | mn USD | %-change |  | mn USD | %-change |  | mn USD | %-change |  | mn USD | %-change |  | mn USD | %-change |
| Δreal GDP (cumulative) | 25,145 |  | 31,590 | 25.6% |  | 28,196 | 12.1% |  | 25,222 | 0.3% |  | 22,649 | -9.9% |  | 20,441 | -18.7% |
| - health pathway (cumulative) | 95 |  | 145 | 52.3% |  | 119 | 24.9% |  | 96 | 0.6% |  | 76 | -20.7% |  | 58 | -39.1% |
| - sales tax pathway (cumulative) | 25,050 |  | 31,445 | 25.5% |  | 28,077 | 12.1% |  | 25,126 | 0.3% |  | 22,573 | -9.9% |  | 20,383 | -18.6% |
|  | mn USD |  | mn USD | %-change |  | mn USD | %-change |  | mn USD | %-change |  | mn USD | %-change |  | mn USD | %-change |
| Δreal GDP (cumulative) | 25,145 |  | 31,590 | 25.6% |  | 28,196 | 12.1% |  | 25,222 | 0.3% |  | 22,649 | -9.9% |  | 20,441 | -18.7% |
| - Private Consumption (cumulative) | -9,882 |  | -14,479 | 46.5% |  | -11,962 | 21.0% |  | -9,931 | 0.5% |  | -8,309 | -15.9% |  | -7,016 | -29.0% |
| - Government Consumption (cumulative) | 8,633 |  | 11,587 | 34.2% |  | 10,003 | 15.9% |  | 8,665 | 0.4% |  | 7,547 | -12.6% |  | 6,617 | -23.4% |
| - Investment (cumulative) | 26,394 |  | 34,483 | 30.6% |  | 30,155 | 14.3% |  | 26,487 | 0.4% |  | 23,410 | -11.3% |  | 20,841 | -21.0% |
| - Exports (cumulative) | 35,857 |  | 44,539 | 24.2% |  | 39,948 | 11.4% |  | 35,956 | 0.3% |  | 32,526 | -9.3% |  | 29,601 | -17.4% |
| - Imports (cumulative) | 35,857 |  | 44,539 | 24.2% |  | 39,948 | 11.4% |  | 35,956 | 0.3% |  | 32,526 | -9.3% |  | 29,601 | -17.4% |
|  | %-points |  | %-points | %-point change |  | %-points | %-point change |  | %-points | %-point change |  | %-points | %-point change |  | %-points | %-point change |
| Δsales tax (long run, 2035) | 53.5% |  | 61.7% | 8.13% |  | 57.6% | 4.09% |  | 53.6% | 0.10% |  | 49.8% | -3.71% |  | 46.3% | -7.26% |
| Δinvestment price index (long run, 2035) | -0.8% |  | -1.2% | -0.32% |  | -1.0% | -0.14% |  | -0.8% | 0.00% |  | -0.7% | 0.11% |  | -0.6% | 0.21% |
| Δreal exchange rate (long run, 2035) | -0.9% |  | -1.2% | -0.32% |  | -1.0% | -0.15% |  | -0.9% | 0.00% |  | -0.7% | 0.11% |  | -0.6% | 0.21% |
| ***Real Consumption (cumulative indicators)*** | | | | | | | | | | | | | | | | |
|  | mn USD |  | mn USD | %-change |  | mn USD | %-change |  | mn USD | %-change |  | mn USD | %-change |  | mn USD | %-change |
| Δreal Household Consumption | -9,882 |  | -14,479 | 46.5% |  | -11,962 | 21.0% |  | -9,931 | 0.5% |  | -8,309 | -15.9% |  | -7,016 | -29.0% |
| - sales tax pathway | -9,930 |  | -14,552 | 46.6% |  | -12,022 | 21.1% |  | -9,979 | 0.5% |  | -8,347 | -15.9% |  | -7,045 | -29.1% |
| - Bangkok^1^ | -362 |  | -693 | 91.6% |  | -508 | 40.6% |  | -357 | -1.3% |  | -234 | -35.3% |  | -134 | -62.8% |
| - Central region (exc Bangkok)^1^ | -3,336 |  | -5,065 | 51.8% |  | -4,128 | 23.8% |  | -3,363 | 0.8% |  | -2,743 | -17.8% |  | -2,243 | -32.7% |
| - North region^1^ | -935 |  | -1,720 | 84.0% |  | -1,287 | 37.6% |  | -943 | 0.9% |  | -673 | -28.0% |  | -462 | -50.6% |
| - Northeast region^1^ | -982 |  | -1,913 | 94.8% |  | -1,403 | 42.8% |  | -992 | 1.0% |  | -666 | -32.2% |  | -407 | -58.6% |
| - South region^1^ | -4,315 |  | -5,161 | 19.6% |  | -4,696 | 8.8% |  | -4,325 | 0.2% |  | -4,031 | -6.6% |  | -3,798 | -12.0% |
| - health pathway | 48 |  | 73 | 52.3% |  | 60 | 24.9% |  | 48 | 0.6% |  | 38 | -20.7% |  | 29 | -39.2% |
| - Bangkok^1^ | 8 |  | 13 | 62.7% |  | 10 | 29.7% |  | 8 | 0.4% |  | 6 | -25.3% |  | 4 | -47.5% |
| - Central region (exc Bangkok)^1^ | 19 |  | 27 | 42.8% |  | 23 | 20.4% |  | 19 | 0.5% |  | 16 | -16.9% |  | 13 | -32.0% |
| - North region^1^ | 7 |  | 10 | 52.0% |  | 9 | 24.8% |  | 7 | 0.6% |  | 5 | -20.6% |  | 4 | -39.0% |
| - Northeast region^1^ | 9 |  | 13 | 50.4% |  | 11 | 24.0% |  | 9 | 0.6% |  | 7 | -19.9% |  | 6 | -37.7% |
| - South region^1^ | 5 |  | 9 | 73.1% |  | 7 | 34.8% |  | 6 | 0.9% |  | 4 | -28.8% |  | 2 | -54.6% |
| Note: Own calculations. ^1^ Regional consumption %-impacts calculated as share of projected regional totals. | | | | | | | | | | | | | | | | |

| **Table C.4.1.3. Sensitivity to palm oil own price elasticity of demand (50% reduction in palm oil consumption): Cumulative impact indicators for 2016-35 (nutrition, biomarker, health)** | | | | | | | | | | | | | | | | |
| --- | --- | --- | --- | --- | --- | --- | --- | --- | --- | --- | --- | --- | --- | --- | --- | --- |
|  | Policy Scenario (elas ≈ -0.94-0.95) |  | Sensitivity Scenario 1. (elas = -0.75) | |  | Sensitivity Scenario 2. (elas = -0.85) | |  | Sensitivity Scenario 3. (elas = -0.95) | |  | Sensitivity Scenario 4. (elas = -1.05) | |  | Sensitivity Scenario 5. (elas = -1.15) | |
| ***Nutrition (long run indicators, 2035)*** |  |  |  |  |  |  |  |  |  |  |  |  |  |  |  |  |
|  | %-points |  | %-points | %-change |  | %-points | %-change |  | %-points | %-change |  | %-points | %-change |  | %-points | %-change |
| ΔSFA energy intake share | -0.322% |  | -0.321% | -0.3% |  | -0.322% | -0.1% |  | -0.322% | 0.0% |  | -0.322% | 0.1% |  | -0.323% | 0.2% |
| ΔMUFA energy intake share | -0.164% |  | -0.144% | -11.9% |  | -0.155% | -5.7% |  | -0.164% | -0.1% |  | -0.172% | 4.7% |  | -0.178% | 8.8% |
| ΔPUFA energy intake share | 0.298% |  | 0.390% | 31.0% |  | 0.341% | 14.7% |  | 0.299% | 0.3% |  | 0.261% | -12.2% |  | 0.229% | -22.9% |
| ***Biomarker (cumulative indicators)*** |  |  |  |  |  |  |  |  |  |  |  |  |  |  |  |  |
|  | cum. chg. |  | cum. chg. | %-change |  | cum. chg. | %-change |  | cum. chg. | %-change |  | cum. chg. | %-change |  | cum. chg. | %-change |
| ΔTotal-to-HDL cholesterol ratio | -0.101 |  | -0.159 | 58.0% |  | -0.129 | 27.6% |  | -0.102 | 0.6% |  | -0.078 | -22.9% |  | -0.057 | -43.3% |
| ***Health (cumulative indicators)*** |  |  |  |  |  |  |  |  |  |  |  |  |  |  |  |  |
|  | cases |  | cases | %-change |  | cases | %-change |  | cases | %-change |  | cases | %-change |  | cases | %-change |
| ΔPatient Incident Cases | -3,570 |  | -5,427 | 52.0% |  | -4,453 | 24.7% |  | -3,590 | 0.6% |  | -2,836 | -20.6% |  | -2,184 | -38.8% |
| - myocardial infarction | -2,704 |  | -4,111 | 52.0% |  | -3,373 | 24.7% |  | -2,719 | 0.6% |  | -2,148 | -20.6% |  | -1,654 | -38.8% |
| - stroke | -866 |  | -1,316 | 52.0% |  | -1,080 | 24.7% |  | -871 | 0.6% |  | -688 | -20.5% |  | -530 | -38.8% |
| ΔPatient premature deaths | -1,861 |  | -2,822 | 51.6% |  | -2,318 | 24.5% |  | -1,872 | 0.6% |  | -1,482 | -20.4% |  | -1,145 | -38.5% |
| - myocardial infarction | -1,560 |  | -2,366 | 51.6% |  | -1,943 | 24.6% |  | -1,569 | 0.6% |  | -1,242 | -20.4% |  | -959 | -38.5% |
| - stroke | -301 |  | -456 | 51.4% |  | -375 | 24.4% |  | -303 | 0.6% |  | -240 | -20.3% |  | -186 | -38.3% |
|  | pers-yrs |  | pers-yrs | %-change |  | pers-yrs | %-change |  | pers-yrs | %-change |  | pers-yrs | %-change |  | pers-yrs | %-change |
| ΔPatient Disease Burden (YLD) | -777 |  | -1,168 | 50.2% |  | -963 | 23.9% |  | -782 | 0.6% |  | -624 | -19.8% |  | -487 | -37.4% |
| - myocardial infarction | -4 |  | -7 | 52.0% |  | -5 | 24.7% |  | -4 | 0.6% |  | -3 | -20.6% |  | -3 | -38.8% |
| - stroke | -773 |  | -1,161 | 50.2% |  | -958 | 23.9% |  | -777 | 0.6% |  | -620 | -19.8% |  | -484 | -37.4% |
| ΔPatient Worktime Loss | -362 |  | -544 | 50.2% |  | -448 | 23.9% |  | -364 | 0.6% |  | -290 | -19.8% |  | -227 | -37.3% |
| - myocardial infarction | -2 |  | -3 | 51.9% |  | -2 | 24.7% |  | -2 | 0.6% |  | -1 | -20.5% |  | -1 | -38.7% |
| - stroke | -360 |  | -541 | 50.2% |  | -446 | 23.9% |  | -362 | 0.6% |  | -289 | -19.7% |  | -226 | -37.3% |
| ΔCaregiver Time Loss | -1,587 |  | -2,407 | 51.7% |  | -1,977 | 24.6% |  | -1,596 | 0.6% |  | -1,263 | -20.4% |  | -975 | -38.5% |
| - stroke | -1,587 |  | -2,407 | 51.7% |  | -1,977 | 24.6% |  | -1,596 | 0.6% |  | -1,263 | -20.4% |  | -975 | -38.5% |
| - work time | -643 |  | -962 | 49.6% |  | -795 | 23.6% |  | -647 | 0.6% |  | -518 | -19.5% |  | -406 | -36.9% |
| - leisure time | -944 |  | -1,445 | 53.1% |  | -1,182 | 25.2% |  | -949 | 0.5% |  | -746 | -21.0% |  | -570 | -39.7% |
|  | mn USD |  | mn USD | %-change |  | mn USD | %-change |  | mn USD | %-change |  | mn USD | %-change |  | mn USD | %-change |
| Δreal Health Expenses |  |  |  |  |  |  |  |  |  |  |  |  |  |  |  |  |
| - formal hospital | -310 |  | -428 | 38.1% |  | -365 | 17.7% |  | -311 | 0.4% |  | -266 | -14.2% |  | -228 | -26.4% |
| - myocardial infarction | -293 |  | -405 | 38.2% |  | -345 | 17.8% |  | -294 | 0.4% |  | -251 | -14.2% |  | -215 | -26.5% |
| - stroke | -17 |  | -24 | 35.8% |  | -20 | 16.5% |  | -18 | 0.4% |  | -15 | -13.1% |  | -13 | -24.3% |
| Note: Own calculations. | | | | | | | | | | | | | | | | |

| **Table C.4.1.4. Sensitivity to palm oil own price elasticity of demand (50% reduction in palm oil consumption): Cumulative impact indicators for 2016-35 (demographic, environment)** | | | | | | | | | | | | | | | | |
| --- | --- | --- | --- | --- | --- | --- | --- | --- | --- | --- | --- | --- | --- | --- | --- | --- |
|  | Policy Scenario (elas ≈ -0.94-0.95) |  | Sensitivity Scenario 1. (elas = -0.75) | |  | Sensitivity Scenario 2. (elas = -0.85) | |  | Sensitivity Scenario 3. (elas = -0.95) | |  | Sensitivity Scenario 4. (elas = -1.05) | |  | Sensitivity Scenario 5. (elas = -1.15) | |
| ***Demographic (cumulative indicators)*** |  |  |  |  |  |  |  |  |  |  |  |  |  |  |  |  |
|  | pers-yrs |  | pers-yrs | %-change |  | pers-yrs | %-change |  | pers-yrs | %-change |  | pers-yrs | %-change |  | pers-yrs | %-change |
| Δpopulation | 13,621 |  | 21,248 | 56.0% |  | 17,248 | 26.6% |  | 13,703 | 0.6% |  | 10,603 | -22.2% |  | 7,918 | -41.9% |
| - Bangkok^1^ | 886 |  | 1,872 | 111.2% |  | 1,348 | 52.1% |  | 883 | -0.4% |  | 475 | -46.4% |  | 120 | -86.4% |
| - Central region (exc Bangkok)^1^ | 7,387 |  | 10,305 | 39.5% |  | 8,779 | 18.9% |  | 7,427 | 0.5% |  | 6,245 | -15.5% |  | 5,221 | -29.3% |
| - North region^1^ | 2,544 |  | 3,945 | 55.0% |  | 3,210 | 26.2% |  | 2,559 | 0.6% |  | 1,990 | -21.8% |  | 1,497 | -41.1% |
| - Northeast region^1^ | 2,721 |  | 4,231 | 55.5% |  | 3,440 | 26.4% |  | 2,738 | 0.7% |  | 2,126 | -21.9% |  | 1,595 | -41.4% |
| - South region^1^ | 83 |  | 896 | 976.1% |  | 471 | 465.8% |  | 95 | 14.3% |  | -233 | -379.6% |  | -516 | -720.1% |
| - urban^1^ | 5,019 |  | 9,674 | 92.8% |  | 7,229 | 44.0% |  | 5,061 | 0.8% |  | 3,166 | -36.9% |  | 1,524 | -69.6% |
| - rural^1^ | 8,602 |  | 11,574 | 34.5% |  | 10,019 | 16.5% |  | 8,642 | 0.5% |  | 7,437 | -13.5% |  | 6,394 | -25.7% |
|  | pers-yrs |  | pers-yrs | %-change |  | pers-yrs | %-change |  | pers-yrs | %-change |  | pers-yrs | %-change |  | pers-yrs | %-change |
| Δworkforce | 4,450 |  | 6,860 | 54.2% |  | 5,596 | 25.8% |  | 4,476 | 0.6% |  | 3,497 | -21.4% |  | 2,649 | -40.5% |
| - urban^1^ | 1,621 |  | 3,048 | 88.0% |  | 2,299 | 41.8% |  | 1,634 | 0.8% |  | 1,053 | -35.0% |  | 551 | -66.0% |
| - rural^1^ | 2,829 |  | 3,812 | 34.8% |  | 3,297 | 16.6% |  | 2,842 | 0.5% |  | 2,443 | -13.6% |  | 2,098 | -25.8% |
| ***Environment (cumulative indicator)*** |  |  |  |  |  |  |  |  |  |  |  |  |  |  |  |  |
|  | Mt CO_2_-eq |  | Mt CO_2_-eq | %-change |  | Mt CO_2_-eq | %-change |  | Mt CO_2_-eq | %-change |  | Mt CO_2_-eq | %-change |  | Mt CO_2_-eq | %-change |
| ΔGHG emissions | 7.52 |  | 7.57 | 0.7% |  | 7.54 | 0.3% |  | 7.51 | -0.1% |  | 7.53 | 0.1% |  | 7.54 | 0.2% |
| Note: Own calculations. | | | | | | | | | | | | | | | | |

| **Table C.4.2.1. Sensitivity Analysis Scenario: edible oil cross-price elasticities** | |
| --- | --- |
| Scenarios | Description |
| Policy Scenario | -50% average household consumption of (palm) cooking oil + palm oil/other edible oils cross-price elasticity ≈ 0.02-0.03 |
| Sensitivity Scenario 1 | '-50% average household consumption of (palm) cooking oil + palm oil/other edible oils cross-price elasticity= 0.00 |
| Sensitivity Scenario 2 | -50% average household consumption of (palm) cooking oil + palm oil/other edible oils cross-price elasticity= 0.05 |
| Sensitivity Scenario 3 | -50% average household consumption of (palm) cooking oil + palm oil/other edible oils cross-price elasticity= 0.10 |
| Sensitivity Scenario 4 | -50% average household consumption of (palm) cooking oil + palm oil/other edible oils cross-price elasticity= 0.15 |
| Sensitivity Scenario 5 | -50% average household consumption of (palm) cooking oil + palm oil/other edible oils cross-price elasticity= 0.20 |
| Sensitivity Scenario 6 | -50% average household consumption of (palm) cooking oil + palm oil/other edible oils cross-price elasticity= 0.25 |
| Instrument | sales tax on palm cooking oil |

### C.4.2. Sensitivity Analyses of edible oil cross-price elasticities in household demand

Sensitivity scenarios 1-6 simulate equidistant variation in cross-price elasticities between palm cooking oil and other edible oils over the range 0.00 to 0.25. The range was chosen to study a range around baseline elasticities (0.02-0.03) and with a focus on positive cross-price elasticities. Scenario specifications are included in Table C.4.2.1. Results are presented in Tables C.4.2.2-C.4.2.4, and, in contrast to the own-price elasticity sensitivity analyses in section C.4.1, they show relatively small variation in sales tax impacts including macroeconomic indicators, but very strong variation in health pathway impacts including nutrition, health and demographic indicators.

The health pathway impacts vary quite strongly: real GDP impacts vary by USD -85mn to USD +1.7bn (policy sim=USD 95mn), real household consumption impacts vary by USD -44mn to USD 865mn (policy sim= USD 48mn), cholesterol biomarker impacts vary from +0.11 to -1.97 (policy sim=-0.10), saved incident cases and saved premature deaths vary, respectively, by -2,880 to +60,848 (policy sim=3,570) and by -1,476 to +31,644 (policy sim=1,861), and population impacts vary by -13,518 to +255,972 (policy sim=13,621).

Aggregate health pathway indicators also vary quite strongly in relative terms: macroeconomic impacts vary between a factor two reduction and factor 17 increase; nutritional impacts between a factor one reduction and a factor eight increase, biomarker impacts between a factor two reduction and a factor 19 increase, health impacts between a factor two reduction and a factor 16 increase, and population impacts between a factor two reduction and a factor 18 increase.

Aggregate health pathway indicator impacts are mixed correlated with cross-price elasticities between palm cooking oil and other edible oils: macroeconomic, nutritional, and demographic indicator impact correlations are positive, while cholesterol biomarker and health outcomes correlations are negative (indicating that larger cross-price elasticities increase beneficial health pathway outcomes across the board, i.e. lead to larger economic gains, larger reductions in cholesterol ratios and incident cases, save more lives, and increase population gains).

The upper bound health pathway results (scenario 6) represent potentially very big health impacts (changes of factor 16-18). However, cross-price elasticities of 0.25 are extreme and not likely to characterize Thai consumption patterns. Nonetheless, we observe that variation of cross-price elasticities in a reasonable range between 0.00-0.05 can change the (relatively small policy sim) health pathway outcomes by a factor two in opposite directions.

Results for the sales tax pathway generally show small variation: The required sales tax rate to achieve the policy target is virtually unchanged (policy sim=53.5%), real GDP impacts vary by USD 25.0-25.1bn (policy sim=USD 25.1bn), and real private consumption impacts vary by USD -9.9bn to USD -10.1bn (policy sim= USD -9.9mn).

Final demand impacts for the combined health and sales tax pathways are dominated by variation in health pathway impacts, but magnitudes remain limited: real GDP impacts vary by USD 25.0-26.7bn (policy sim=USD 25.1bn), real private consumption impacts vary by USD -9.2bn to USD -10.0bn (policy sim=USD -9.9bn), real government consumption impacts vary by USD 8.0-8.7bn (policy sim=USD 8.6bn), real investment impacts vary by USD 26.2-27.9bn (policy sim=USD 26.4bn), and real trade aggregate impacts vary by USD 35.6-38.2bn (policy sim=USD 35.9bn).

Similar to marginal health pathway impacts, aggregate real GDP and final demand impacts are positively correlated with cross-price elasticities between palm cooking oil and other edible oils. The main exception is government consumption which has a small negative correlation with real GDP impacts due to positively correlated relative government price impacts (because of strong variation in demand for health service demand).

LUC-related GHG emissions impacts vary by 7.50-7.88 Mt CO_2_-eq (policy sim=7.52 Mt) or -0.2% to +4.7%, and it is positively correlated with cross-price elasticities between palm cooking oil and other edible oils.

Overall, while sales tax pathway impacts remain unaffected, health pathway impacts turn out to be very sensitive to the specification of cross-price elasticities between palm cooking oil and other edible oils. While our upper bound cross-price elasticity of 0.25 is extreme, variation of cross-price elasticities in a reasonable range between 0.00-0.05 can change our health pathway outcomes by factors of ±2. Hence, while macroeconomic and environmental outcomes are relatively unaffected and fairly robust, our nutrition, health and demographic indicator impacts turn out to be very sensitive to variation in cross-price elasticities between palm cooking oil and other edible oils.

| **Table C.4.2.2. Sensitivity to palm oil-to-edible oil cross-price elasticity of demand (50% reduction in palm oil consumption): Cumulative impact indicators for 2016-35 (economic)** | | | | | | | | | | | | | | | | | | | |
| --- | --- | --- | --- | --- | --- | --- | --- | --- | --- | --- | --- | --- | --- | --- | --- | --- | --- | --- | --- |
|  | Policy Scenario  (elas ≈ 0.02-0.03) |  | Sensitivity Scen. 1.  (elas = 0.00) | |  | Sensitivity Scen. 2.  (elas = 0.05) | |  | Sensitivity Scen. 3.  (elas = 0.10) | |  | Sensitivity Scen. 4.  (elas = 0.15) | |  | Sensitivity Scen. 5.  (elas = 0.20) | |  | Sensitivity Scen. 6.  (elas = 0.25) | |
| ***Real GDP & sales tax & price indices***  ***(cumulative and long run indicators)*** | | | | | | | | | | | | | |  |  |  |  |  |  |
|  | mn USD |  | mn USD | %-change |  | mn USD | %-change |  | mn USD | %-change |  | mn USD | %-change |  | mn USD | %-change |  | mn USD | %-change |
| Δreal GDP (cumulative) | 25,145 |  | 24,971 | -0.7% |  | 25,337 | 0.8% |  | 25,691 | 2.2% |  | 26,036 | 3.5% |  | 26,373 | 4.9% |  | 26,701 | 6.2% |
| - health pathway (cumulative) | 95 |  | -85 | -190% |  | 294 | 208% |  | 660 | 593% |  | 1,017 | 967% |  | 1,365 | 1332% |  | 1,703 | 1687% |
| - sales tax pathway (cumulative) | 25,050 |  | 25,056 | 0.0% |  | 25,043 | 0.0% |  | 25,031 | -0.1% |  | 25,020 | -0.1% |  | 25,009 | -0.2% |  | 24,998 | -0.2% |
|  | mn USD |  | mn USD | %-change |  | mn USD | %-change |  | mn USD | %-change |  | mn USD | %-change |  | mn USD | %-change |  | mn USD | %-change |
| Δreal GDP (cumulative) | 25,145 |  | 24,971 | -0.7% |  | 25,337 | 0.8% |  | 25,691 | 2.2% |  | 26,036 | 3.5% |  | 26,373 | 4.9% |  | 26,701 | 6.2% |
| - Private Consumption (cumulative) | -9,882 |  | -9,961 | 0.8% |  | -9,795 | -0.9% |  | -9,636 | -2.5% |  | -9,481 | -4.1% |  | -9,331 | -5.6% |  | -9,186 | -7.0% |
| - Government Consumption (cumulative) | 8,633 |  | 8,707 | 0.9% |  | 8,553 | -0.9% |  | 8,404 | -2.6% |  | 8,261 | -4.3% |  | 8,122 | -5.9% |  | 7,988 | -7.5% |
| - Investment (cumulative) | 26,394 |  | 26,225 | -0.6% |  | 26,580 | 0.7% |  | 26,922 | 2.0% |  | 27,257 | 3.3% |  | 27,582 | 4.5% |  | 27,900 | 5.7% |
| - Exports (cumulative) | 35,857 |  | 35,603 | -0.7% |  | 36,138 | 0.8% |  | 36,659 | 2.2% |  | 37,171 | 3.7% |  | 37,676 | 5.1% |  | 38,173 | 6.5% |
| - Imports (cumulative) | 35,857 |  | 35,603 | -0.7% |  | 36,138 | 0.8% |  | 36,659 | 2.2% |  | 37,171 | 3.7% |  | 37,676 | 5.1% |  | 38,173 | 6.5% |
|  | %-points |  | %-points | %-point change |  | %-points | %-point change |  | %-points | %-point change |  | %-points | %-point change |  | %-points | %-point change |  | %-points | %-point change |
| Δsales tax (long run, 2035) | 53.5% |  | 53.5% | 0.01% |  | 53.5% | -0.01% |  | 53.5% | -0.02% |  | 53.5% | -0.04% |  | 53.5% | -0.05% |  | 53.5% | -0.07% |
| Δinvestment price index (long run, 2035) | -0.8% |  | -0.8% | 0.00% |  | -0.8% | 0.00% |  | -0.8% | 0.00% |  | -0.8% | 0.00% |  | -0.8% | 0.00% |  | -0.8% | 0.00% |
| Δreal exchange rate (long run, 2035) | -0.9% |  | -0.9% | 0.00% |  | -0.9% | 0.00% |  | -0.9% | 0.00% |  | -0.9% | 0.00% |  | -0.9% | 0.00% |  | -0.9% | 0.00% |
| ***Real Consumption (cumulative indicators)*** | | | | | | | | | | | | | | | | | | | |
|  | mn USD |  | mn USD | %-change |  | mn USD | %-change |  | mn USD | %-change |  | mn USD | %-change |  | mn USD | %-change |  | mn USD | %-change |
| Δreal Household Consumption | -9,882 |  | -9,961 | 0.8% |  | -9,795 | -0.9% |  | -9,636 | -2.5% |  | -9,481 | -4.1% |  | -9,331 | -5.6% |  | -9,186 | -7.0% |
| - sales tax pathway | -9,930 |  | -9,917 | -0.1% |  | -9,944 | 0.1% |  | -9,970 | 0.4% |  | -9,997 | 0.7% |  | -10,024 | 1.0% |  | -10,051 | 1.2% |
| - Bangkok^1^ | -362 |  | -350 | -3.1% |  | -373 | 3.2% |  | -396 | 9.5% |  | -419 | 15.8% |  | -442 | 22.1% |  | -464 | 28.4% |
| - Central region (exc Bangkok)^1^ | -3,336 |  | -3,343 | 0.2% |  | -3,328 | -0.2% |  | -3,314 | -0.7% |  | -3,300 | -1.1% |  | -3,285 | -1.5% |  | -3,271 | -1.9% |
| - North region^1^ | -935 |  | -942 | 0.8% |  | -926 | -0.9% |  | -911 | -2.6% |  | -895 | -4.2% |  | -880 | -5.9% |  | -864 | -7.6% |
| - Northeast region^1^ | -982 |  | -978 | -0.5% |  | -988 | 0.5% |  | -997 | 1.5% |  | -1,007 | 2.5% |  | -1,016 | 3.5% |  | -1,026 | 4.4% |
| - South region^1^ | -4,315 |  | -4,303 | -0.3% |  | -4,328 | 0.3% |  | -4,352 | 0.9% |  | -4,377 | 1.4% |  | -4,401 | 2.0% |  | -4,426 | 2.6% |
| - health pathway | 48 |  | -44 | -191% |  | 149 | 210% |  | 335 | 598% |  | 516 | 976% |  | 693 | 1344% |  | 865 | 1702% |
| - Bangkok^1^ | 8 |  | -9 | -219% |  | 25 | 221% |  | 59 | 647% |  | 91 | 1062% |  | 123 | 1467% |  | 154 | 1862% |
| - Central region (exc Bangkok)^1^ | 19 |  | -10 | -153% |  | 52 | 176% |  | 112 | 493% |  | 170 | 802% |  | 227 | 1103% |  | 282 | 1396% |
| - North region^1^ | 7 |  | -7 | -197% |  | 21 | 213% |  | 49 | 609% |  | 75 | 996% |  | 101 | 1373% |  | 126 | 1740% |
| - Northeast region^1^ | 9 |  | -8 | -189% |  | 27 | 207% |  | 62 | 590% |  | 95 | 963% |  | 128 | 1328% |  | 160 | 1684% |
| - South region^1^ | 5 |  | -10 | -282% |  | 23 | 313% |  | 54 | 887% |  | 84 | 1445% |  | 114 | 1989% |  | 143 | 2518% |
| Note: Own calculations. ^1^ Regional consumption %-impacts calculated as share of projected regional totals. | | | | | | | | | | | | | | | | | | | |

| **Table C.4.2.3. Sensitivity to palm oil-to-edible oil cross-price elasticity of demand (50% reduction in palm oil consumption): Cumulative impact indicators for 2016-35 (nutrition, biomarker, health)** | | | | | | | | | | | | | | | | | | | |
| --- | --- | --- | --- | --- | --- | --- | --- | --- | --- | --- | --- | --- | --- | --- | --- | --- | --- | --- | --- |
|  | Policy Scenario  (elas ≈ 0.02-0.03) |  | Sensitivity Scen. 1.  (elas = 0.00) | |  | Sensitivity Scen. 2.  (elas = 0.05) | |  | Sensitivity Scen. 3.  (elas = 0.10) | |  | Sensitivity Scen. 4.  (elas = 0.15) | |  | Sensitivity Scen. 5.  (elas = 0.20) | |  | Sensitivity Scen. 6.  (elas = 0.25) | |
| ***Nutrition (long run indicators, 2035)*** | | | | | | | | | | | | | | | | | | | |
|  | %-points |  | %-points | %-change |  | %-points | %-change |  | %-points | %-change |  | %-points | %-change |  | %-points | %-change |  | %-points | %-change |
| ΔSFA energy intake share | -0.322% |  | -0.346% | 8% |  | -0.295% | -8% |  | -0.245% | -24% |  | -0.197% | -39% |  | -0.149% | -54% |  | -0.103% | -68% |
| ΔMUFA energy intake share | -0.164% |  | -0.252% | 54% |  | -0.067% | -59% |  | 0.113% | -169% |  | 0.288% | -276% |  | 0.459% | -380% |  | 0.625% | -481% |
| ΔPUFA energy intake share | 0.298% |  | 0.028% | -90% |  | 0.593% | 99% |  | 1.138% | 282% |  | 1.670% | 461% |  | 2.189% | 636% |  | 2.696% | 806% |
| ***Biomarker (cumulative indicators)*** | | | | | | | | | | | | | | | | | | | |
|  | cum. chg. |  | cum. chg. | %-change |  | cum. chg. | %-change |  | cum. chg. | %-change |  | cum. chg. | %-change |  | cum. chg. | %-change |  | cum. chg. | %-change |
| ΔTotal-to-HDL cholesterol ratio | -0.101 |  | 0.109 | -208% |  | -0.331 | 228% |  | -0.756 | 649% |  | -1.171 | 1061% |  | -1.576 | 1462% |  | -1.971 | 1854% |
| ***Health (cumulative indicators)*** | | | | | | | | | | | | | | | | | | | |
|  | cases |  | cases | %-change |  | cases | %-change |  | cases | %-change |  | cases | %-change |  | cases | %-change |  | cases | %-change |
| ΔPatient Incident Cases | -3,570 |  | 2,880 | -181% |  | -10,647 | 198% |  | -23,700 | 564% |  | -36,410 | 920% |  | -48,789 | 1267% |  | -60,848 | 1604% |
| - myocardial infarction | -2,704 |  | 2,188 | -181% |  | -8,068 | 198% |  | -17,945 | 564% |  | -27,549 | 919% |  | -36,888 | 1264% |  | -45,972 | 1600% |
| - stroke | -866 |  | 693 | -180% |  | -2,580 | 198% |  | -5,755 | 565% |  | -8,862 | 923% |  | -11,902 | 1274% |  | -14,876 | 1618% |
| ΔPatient premature deaths | -1,861 |  | 1,476 | -179% |  | -5,529 | 197% |  | -12,302 | 561% |  | -18,910 | 916% |  | -25,356 | 1262% |  | -31,644 | 1600% |
| - myocardial infarction | -1,560 |  | 1,242 | -180% |  | -4,635 | 197% |  | -10,304 | 560% |  | -15,822 | 914% |  | -21,195 | 1259% |  | -26,427 | 1594% |
| - stroke | -301 |  | 234 | -178% |  | -894 | 197% |  | -1,999 | 563% |  | -3,088 | 925% |  | -4,161 | 1281% |  | -5,217 | 1632% |
|  | pers-yrs |  | pers-yrs | %-change |  | pers-yrs | %-change |  | pers-yrs | %-change |  | pers-yrs | %-change |  | pers-yrs | %-change |  | pers-yrs | %-change |
| ΔPatient Disease Burden (YLD) | -777 |  | 592 | -176% |  | -2,306 | 197% |  | -5,115 | 558% |  | -7,863 | 912% |  | -10,551 | 1258% |  | -13,180 | 1596% |
| - myocardial infarction | -4 |  | 4 | -181% |  | -13 | 198% |  | -29 | 564% |  | -44 | 919% |  | -59 | 1264% |  | -74 | 1600% |
| - stroke | -773 |  | 588 | -176% |  | -2,293 | 197% |  | -5,086 | 558% |  | -7,819 | 912% |  | -10,492 | 1258% |  | -13,106 | 1596% |
| ΔPatient Worktime Loss | -362 |  | 277 | -177% |  | -1,077 | 197% |  | -2,390 | 560% |  | -3,675 | 915% |  | -4,932 | 1263% |  | -6,162 | 1602% |
| - myocardial infarction | -2 |  | 1 | -181% |  | -6 | 198% |  | -12 | 563% |  | -19 | 918% |  | -25 | 1263% |  | -32 | 1598% |
| - stroke | -360 |  | 276 | -177% |  | -1,071 | 197% |  | -2,378 | 560% |  | -3,656 | 915% |  | -4,907 | 1263% |  | -6,130 | 1602% |
| ΔCaregiver Time Loss | -1,587 |  | 1,260 | -179% |  | -4,726 | 198% |  | -10,532 | 564% |  | -16,214 | 922% |  | -21,772 | 1272% |  | -27,210 | 1614% |
| - stroke | -1,587 |  | 1,260 | -179% |  | -4,726 | 198% |  | -10,532 | 564% |  | -16,214 | 922% |  | -21,772 | 1272% |  | -27,210 | 1614% |
| - work time | -643 |  | 481 | -175% |  | -1,905 | 196% |  | -4,218 | 556% |  | -6,481 | 908% |  | -8,694 | 1252% |  | -10,858 | 1589% |
| - leisure time | -944 |  | 779 | -183% |  | -2,821 | 199% |  | -6,314 | 569% |  | -9,733 | 931% |  | -13,078 | 1285% |  | -16,352 | 1632% |
|  | mn USD |  | mn USD | %-change |  | mn USD | %-change |  | mn USD | %-change |  | mn USD | %-change |  | mn USD | %-change |  | mn USD | %-change |
| Δreal Health Expenses |  |  |  |  |  |  |  |  |  |  |  |  |  |  |  |  |  |  |  |
| - formal hospital | -310 |  | -196 | -37% |  | -435 | 40% |  | -666 | 115% |  | -890 | 187% |  | -1,108 | 257% |  | -1,321 | 326% |
| - myocardial infarction | -293 |  | -181 | -38% |  | -416 | 42% |  | -642 | 119% |  | -862 | 194% |  | -1,076 | 268% |  | -1,284 | 339% |
| - stroke | -17 |  | -15 | -12% |  | -20 | 13% |  | -24 | 38% |  | -28 | 62% |  | -32 | 86% |  | -36 | 109% |
| Note: Own calculations. | | | | | | | | | | | | | | | | | | | |

| **Table C.4.2.4. Sensitivity to palm oil-to-edible oil cross-price elasticity of demand (50% reduction in palm oil consumption): Cumulative impact indicators for 2016-35 (demographic, environment)** | | | | | | | | | | | | | | | | | | | |
| --- | --- | --- | --- | --- | --- | --- | --- | --- | --- | --- | --- | --- | --- | --- | --- | --- | --- | --- | --- |
|  | Policy Scenario  (elas ≈ 0.02-0.03) |  | Sensitivity Scen. 1.  (elas = 0.00) | |  | Sensitivity Scen. 2.  (elas = 0.05) | |  | Sensitivity Scen. 3.  (elas = 0.10) | |  | Sensitivity Scen. 4.  (elas = 0.15) | |  | Sensitivity Scen. 5.  (elas = 0.20) | |  | Sensitivity Scen. 6.  (elas = 0.25) | |
| ***Demographic (cumulative indicators)*** |  |  |  |  |  |  |  |  |  |  |  |  |  |  |  |  |  |  |  |
|  | pers-yrs |  | pers-yrs | %-change |  | pers-yrs | %-change |  | pers-yrs | %-change |  | pers-yrs | %-change |  | pers-yrs | %-change |  | pers-yrs | %-change |
| Δpopulation | 13,621 |  | -13,518 | -199% |  | 43,332 | 218% |  | 98,390 | 622% |  | 152,168 | 1017% |  | 204,687 | 1403% |  | 255,972 | 1779% |
| - Bangkok^1^ | 886 |  | -2,112 | -338% |  | 3,339 | 277% |  | 8,668 | 878% |  | 13,900 | 1468% |  | 19,035 | 2047% |  | 24,076 | 2616% |
| - Central region (exc Bangkok)^1^ | 7,387 |  | -2,569 | -135% |  | 19,181 | 160% |  | 40,162 | 444% |  | 60,571 | 720% |  | 80,417 | 989% |  | 99,711 | 1250% |
| - North region^1^ | 2,544 |  | -2,815 | -211% |  | 8,196 | 222% |  | 18,845 | 641% |  | 29,247 | 1050% |  | 39,408 | 1449% |  | 49,333 | 1839% |
| - Northeast region^1^ | 2,721 |  | -2,753 | -201% |  | 8,704 | 220% |  | 19,876 | 631% |  | 30,856 | 1034% |  | 41,646 | 1431% |  | 52,249 | 1820% |
| - South region^1^ | 83 |  | -3,269 | -4028% |  | 3,912 | 4600% |  | 10,840 | 12924% |  | 17,595 | 21040% |  | 24,181 | 28954% |  | 30,603 | 36670% |
| - urban^1^ | 5,019 |  | -10,118 | -302% |  | 20,838 | 315% |  | 50,996 | 916% |  | 80,563 | 1505% |  | 109,545 | 2083% |  | 137,950 | 2649% |
| - rural^1^ | 8,602 |  | -3,400 | -140% |  | 22,494 | 161% |  | 47,395 | 451% |  | 71,605 | 732% |  | 95,142 | 1006% |  | 118,022 | 1272% |
|  | persons |  | persons | %-change |  | persons | %-change |  | persons | %-change |  | persons | %-change |  | persons | %-change |  | persons | %-change |
| Δworkforce | 4,450 |  | -4,118 | -193% |  | 13,902 | 212% |  | 31,402 | 606% |  | 48,538 | 991% |  | 65,308 | 1368% |  | 81,714 | 1736% |
| - urban^1^ | 1,621 |  | -3,005 | -285% |  | 6,481 | 300% |  | 15,752 | 872% |  | 24,865 | 1434% |  | 33,819 | 1986% |  | 42,613 | 2529% |
| - rural^1^ | 2,829 |  | -1,112 | -139% |  | 7,420 | 162% |  | 15,650 | 453% |  | 23,673 | 737% |  | 31,489 | 1013% |  | 39,101 | 1282% |
| ***Environment (cumulative indicator)*** |  |  |  |  |  |  |  |  |  |  |  |  |  |  |  |  |  |  |  |
|  | Mt CO_2_-eq |  | Mt CO_2_-eq | %-change |  | Mt CO_2_-eq | %-change |  | Mt CO_2_-eq | %-change |  | Mt CO_2_-eq | %-change |  | Mt CO_2_-eq | %-change |  | Mt CO_2_-eq | %-change |
| ΔGHG emissions | 7.52 |  | 7.50 | -0.2% |  | 7.54 | 0.3% |  | 7.79 | 3.6% |  | 7.81 | 3.8% |  | 7.84 | 4.3% |  | 7.88 | 4.7% |
| Note: Own calculations. | | | | | | | | | | | | | | | | | | | |

| **Table C.4.3.1. Sensitivity Analysis Scenario: palm oil income elasticities** | |
| --- | --- |
| Scenarios | Description |
| Policy Scenario | -50% average household consumption of (palm) cooking oil + palm oil income elasticity ≈ 1.12-1.14 |
| Sensitivity Scenario 1 | -50% average household consumption of (palm) cooking oil + palm oil income elasticity = 0.90 |
| Sensitivity Scenario 2 | -50% average household consumption of (palm) cooking oil + palm oil income elasticity = 1.00 |
| Sensitivity Scenario 3 | -50% average household consumption of (palm) cooking oil + palm oil income elasticity = 1.10 |
| Sensitivity Scenario 4 | -50% average household consumption of (palm) cooking oil + palm oil income elasticity = 1.20 |
| Sensitivity Scenario 5 | -50% average household consumption of (palm) cooking oil + palm oil income elasticity = 1.30 |
| Instrument | sales tax on palm cooking oil |

### C.4.3. Sensitivity Analyses of palm oil income elasticities in household demand

Sensitivity scenarios 1-5 simulate equidistant variation in palm cooking oil income elasticities over the range 0.90 to 1.30. The range was chosen to study a symmetric range around baseline elasticities (1.12-1.14). Scenario specifications are included in Table C.4.3.1. Results are presented in Tables C.4.3.2-C.4.3.4, and they show moderate variation in health pathway impacts: real GDP impacts vary by USD 89-119mn (policy sim=USD 95mn), real household consumption impacts vary by USD 45-60mn (policy sim=USD 48mn), cholesterol biomarker impacts vary from -0.09 to -0.13 (policy sim=-0.10), saved incident cases and saved premature deaths vary, respectively, by 3,193-4,601 (policy sim=3,570) and by 1,665-2,397 (policy sim=1,861), and population impacts vary by 12,441-17,468 (policy sim=13,621).

In general, aggregate health pathway indicators vary moderately in relative terms: macroeconomic impacts vary between -7% and +25%; nutritional impacts between -34% and +29%, biomarker impacts between -12% and +32%, health impacts between -9% and +30%, and population impacts between -9% and +28%. All macroeconomic health pathway indicator impacts are negatively correlated with the palm cooking oil income elasticity, while biomarker and health outcomes show positive correlations (indicating that higher elasticities reduce beneficial outcomes, i.e. lead to smaller reductions in cholesterol ratios and incident cases, and save fewer lives).

Results for the sales tax pathway generally show similar moderate variation: The required sales tax rate to achieve the policy target vary by 53.2%-54.6% (policy sim=53.5%), real GDP impacts vary by USD 24.2-25.3bn (policy sim=USD 25.1bn), and real private consumption impacts vary by USD -10.1bn to USD -10.3bn (policy sim=USD -9.9bn).

Final demand impacts for the combined health and sales tax pathways are dominated by variation in sales tax pathway impacts, but magnitudes are small: real GDP impacts vary by USD 24.4-25.4bn (policy sim=USD 25.1bn), real private consumption impacts vary by USD-10.0bn to USD -10.3bn (policy sim=USD -9.9bn), real government consumption impacts vary by USD 8.6-8.7bn (policy sim=USD 8.6bn), real investment impacts vary by USD 25.7-27.0bn (policy sim=USD 26.4bn), and real trade aggregate impacts vary by USD 34.8-36.7bn (policy sim=USD 35.9bn).

Real GDP and investment impacts are positively correlated with palm cooking oil income elasticities, while real private and government consumption show small negative correlations. In either case, relative changes are always small in the range of -3% to +4%.

LUC-related GHG emissions vary moderately by 7.08-8.53 Mt CO_2_-eq (policy sim=7.52 Mt) or -5.9% to +13.4%, and emissions impacts are positively correlated with palm cooking oil own income elasticities suggesting that higher income feedback effects for palm cooking oil moderates the required price response (i.e. required sales tax) and thereby lessen substitution effects towards other (cereal) crops with beneficial carbon sequestration characteristics.

Overall, while macroeconomic sales tax pathway impacts are limited, all health pathway impacts including nutritional and biomarker outcomes, health outcomes, and demographic impacts turn out to be moderately sensitive to palm cooking oil own income elasticities, and the same goes for LUC-related GHG emissions impacts. However, most macroeconomic indicators only vary by ±4% and aggregate nutrition, health, demographic, and environmental indicators by ±30% at most, and we observe no sign changes. In that sense, our results turn out to be fairly robust to variation in palm cooking oil income elasticities.

| **Table C.4.3.2. Sensitivity to palm oil income elasticity of demand (50% reduction in palm oil consumption): Cumulative impact indicators for 2016-35 (economic)** | | | | | | | | | | | | | | | | |
| --- | --- | --- | --- | --- | --- | --- | --- | --- | --- | --- | --- | --- | --- | --- | --- | --- |
|  | Policy Scenario  (elas ≈ 1.12-1.14) |  | Sensitivity Scenario 1.  (elas = 0.9) | |  | Sensitivity Scenario 2.  (elas = 1.0) | |  | Sensitivity Scenario 3.  (elas = 1.1) | |  | Sensitivity Scenario 4.  (elas = 1.2) | |  | Sensitivity Scenario 5.  (elas = 1.3) | |
| ***Real GDP & sales tax & price indices***  ***(cumulative and long run indicators)*** | | | | | | | | | | | | | | | | |
|  | mn USD |  | mn USD | %-change |  | mn USD | %-change |  | mn USD | %-change |  | mn USD | %-change |  | mn USD | %-change |
| Δreal GDP (cumulative) | 25,145 |  | 24,370 | -3.1% |  | 24,588 | -2.2% |  | 24,827 | -1.3% |  | 25,083 | -0.2% |  | 25,352 | 0.8% |
| - health pathway (cumulative) | 95 |  | 119 | 24.6% |  | 111 | 16.4% |  | 103 | 8.5% |  | 96 | 0.9% |  | 89 | -6.5% |
| - sales tax pathway (cumulative) | 25,050 |  | 24,251 | -3.2% |  | 24,477 | -2.3% |  | 24,724 | -1.3% |  | 24,987 | -0.3% |  | 25,263 | 0.8% |
|  | mn USD |  | mn USD | %-change |  | mn USD | %-change |  | mn USD | %-change |  | mn USD | %-change |  | mn USD | %-change |
| Δreal GDP (cumulative) | 25,145 |  | 24,370 | -3.1% |  | 24,588 | -2.2% |  | 24,827 | -1.3% |  | 25,083 | -0.2% |  | 25,352 | 0.8% |
| - Private Consumption (cumulative) | -9,882 |  | -10,048 | 1.7% |  | -10,068 | 1.9% |  | -10,119 | 2.4% |  | -10,190 | 3.1% |  | -10,278 | 4.0% |
| - Government Consumption (cumulative) | 8,633 |  | 8,711 | 0.9% |  | 8,656 | 0.3% |  | 8,619 | -0.2% |  | 8,595 | -0.4% |  | 8,581 | -0.6% |
| - Investment (cumulative) | 26,394 |  | 25,707 | -2.6% |  | 26,001 | -1.5% |  | 26,327 | -0.3% |  | 26,678 | 1.1% |  | 27,049 | 2.5% |
| - Exports (cumulative) | 35,857 |  | 34,842 | -2.8% |  | 35,263 | -1.7% |  | 35,710 | -0.4% |  | 36,178 | 0.9% |  | 36,661 | 2.2% |
| - Imports (cumulative) | 35,857 |  | 34,842 | -2.8% |  | 35,263 | -1.7% |  | 35,710 | -0.4% |  | 36,178 | 0.9% |  | 36,661 | 2.2% |
|  | %-points |  | %-points | %-point change |  | %-points | %-point change |  | %-points | %-point change |  | %-points | %-point change |  | %-points | %-point change |
| Δsales tax (long run, 2035) | 53.5% |  | 54.6% | 1.09% |  | 54.1% | 0.57% |  | 53.7% | 0.17% |  | 53.4% | -0.13% |  | 53.2% | -0.37% |
| Δinvestment price index (long run, 2035) | -0.8% |  | -0.9% | -0.02% |  | -0.9% | -0.01% |  | -0.8% | 0.00% |  | -0.8% | 0.00% |  | -0.8% | 0.00% |
| Δreal exchange rate (long run, 2035) | -0.9% |  | -0.9% | -0.02% |  | -0.9% | -0.01% |  | -0.9% | -0.01% |  | -0.9% | 0.00% |  | -0.9% | 0.00% |
| ***Real Consumption (cumulative indicators)*** | | | | | | | | | | | | | | | | |
|  | mn USD |  | mn USD | %-change |  | mn USD | %-change |  | mn USD | %-change |  | mn USD | %-change |  | mn USD | %-change |
| Δreal Household Consumption | -9,882 |  | -10,048 | 1.7% |  | -10,068 | 1.9% |  | -10,119 | 2.4% |  | -10,190 | 3.1% |  | -10,278 | 4.0% |
| - sales tax pathway | -9,930 |  | -10,108 | 1.8% |  | -10,124 | 2.0% |  | -10,171 | 2.4% |  | -10,239 | 3.1% |  | -10,323 | 4.0% |
| - Bangkok^1^ | -362 |  | -631 | 74.5% |  | -537 | 48.6% |  | -449 | 24.2% |  | -364 | 0.7% |  | -282 | -22.1% |
| - Central region (exc Bangkok)^1^ | -3,336 |  | -3,426 | 2.7% |  | -3,421 | 2.5% |  | -3,425 | 2.7% |  | -3,436 | 3.0% |  | -3,452 | 3.5% |
| - North region^1^ | -935 |  | -1,014 | 8.5% |  | -996 | 6.5% |  | -981 | 5.0% |  | -970 | 3.7% |  | -960 | 2.7% |
| - Northeast region^1^ | -982 |  | -1,192 | 21.4% |  | -1,113 | 13.3% |  | -1,040 | 5.8% |  | -971 | -1.2% |  | -905 | -7.8% |
| - South region^1^ | -4,315 |  | -3,844 | -10.9% |  | -4,057 | -6.0% |  | -4,276 | -0.9% |  | -4,499 | 4.3% |  | -4,724 | 9.5% |
| - health pathway | 48 |  | 60 | 24.2% |  | 56 | 16.2% |  | 52 | 8.5% |  | 49 | 1.1% |  | 45 | -6.1% |
| - Bangkok^1^ | 8 |  | 10 | 28.8% |  | 9 | 18.7% |  | 9 | 9.0% |  | 8 | -0.4% |  | 7 | -9.6% |
| - Central region (exc Bangkok)^1^ | 19 |  | 22 | 18.9% |  | 21 | 13.0% |  | 20 | 7.3% |  | 19 | 1.9% |  | 18 | -3.4% |
| - North region^1^ | 7 |  | 9 | 24.7% |  | 8 | 16.7% |  | 7 | 8.9% |  | 7 | 1.5% |  | 6 | -5.8% |
| - Northeast region^1^ | 9 |  | 11 | 22.5% |  | 10 | 15.3% |  | 10 | 8.3% |  | 9 | 1.6% |  | 8 | -5.0% |
| - South region^1^ | 5 |  | 8 | 37.7% |  | 7 | 24.5% |  | 6 | 11.8% |  | 5 | -0.5% |  | 5 | -12.4% |
| Note: Own calculations. ^1^ Regional consumption %-impacts calculated as share of projected regional totals. | | | | | | | | | | | | | | | | |

| **Table C.4.3.3. Sensitivity to palm oil income elasticity of demand (50% reduction in palm oil consumption): Cumulative impact indicators for 2016-35 (nutrition, biomarker, health)** | | | | | | | | | | | | | | | | |
| --- | --- | --- | --- | --- | --- | --- | --- | --- | --- | --- | --- | --- | --- | --- | --- | --- |
|  | Policy Scenario  (elas ≈ 1.12-1.14) |  | Sensitivity Scenario 1.  (elas = 0.9) | |  | Sensitivity Scenario 2.  (elas = 1.0) | |  | Sensitivity Scenario 3.  (elas = 1.1) | |  | Sensitivity Scenario 4.  (elas = 1.2) | |  | Sensitivity Scenario 5.  (elas = 1.3) | |
| ***Nutrition (long run indicators, 2035)*** |  |  |  |  |  |  |  |  |  |  |  |  |  |  |  |  |
|  | %-points |  | %-points | %-change |  | %-points | %-change |  | %-points | %-change |  | %-points | %-change |  | %-points | %-change |
| ΔSFA energy intake share | -0.322% |  | -0.255% | -20.9% |  | -0.289% | -10.2% |  | -0.322% | 0.1% |  | -0.355% | 10.3% |  | -0.387% | 20.2% |
| ΔMUFA energy intake share | -0.164% |  | -0.108% | -34.0% |  | -0.135% | -17.7% |  | -0.161% | -1.8% |  | -0.186% | 13.5% |  | -0.211% | 28.5% |
| ΔPUFA energy intake share | 0.298% |  | 0.338% | 13.7% |  | 0.324% | 8.9% |  | 0.311% | 4.7% |  | 0.300% | 0.8% |  | 0.289% | -2.8% |
| ***Biomarker (cumulative indicators)*** |  |  |  |  |  |  |  |  |  |  |  |  |  |  |  |  |
|  | cum. chg. |  | cum. chg. | %-change |  | cum. chg. | %-change |  | cum. chg. | %-change |  | cum. chg. | %-change |  | cum. chg. | %-change |
| ΔTotal-to-HDL cholesterol ratio | -0.101 |  | -0.133 | 32.0% |  | -0.121 | 20.4% |  | -0.110 | 9.2% |  | -0.099 | -1.4% |  | -0.089 | -11.7% |
| ***Health (cumulative indicators)*** |  |  |  |  |  |  |  |  |  |  |  |  |  |  |  |  |
|  | cases |  | cases | %-change |  | cases | %-change |  | cases | %-change |  | cases | %-change |  | cases | %-change |
| ΔPatient Incident Cases | -3,570 |  | -4,601 | 28.9% |  | -4,223 | 18.3% |  | -3,865 | 8.3% |  | -3,523 | -1.3% |  | -3,193 | -10.6% |
| - myocardial infarction | -2,704 |  | -3,485 | 28.9% |  | -3,199 | 18.3% |  | -2,928 | 8.3% |  | -2,669 | -1.3% |  | -2,419 | -10.6% |
| - stroke | -866 |  | -1,116 | 28.9% |  | -1,024 | 18.3% |  | -937 | 8.2% |  | -854 | -1.4% |  | -774 | -10.6% |
| ΔPatient premature deaths | -1,861 |  | -2,397 | 28.7% |  | -2,200 | 18.2% |  | -2,014 | 8.2% |  | -1,836 | -1.4% |  | -1,665 | -10.6% |
| - myocardial infarction | -1,560 |  | -2,009 | 28.8% |  | -1,844 | 18.2% |  | -1,688 | 8.2% |  | -1,539 | -1.4% |  | -1,395 | -10.6% |
| - stroke | -301 |  | -388 | 28.6% |  | -356 | 18.1% |  | -326 | 8.2% |  | -297 | -1.4% |  | -269 | -10.6% |
|  | pers-yrs |  | pers-yrs | %-change |  | pers-yrs | %-change |  | pers-yrs | %-change |  | pers-yrs | %-change |  | pers-yrs | %-change |
| ΔPatient Disease Burden (YLD) | -777 |  | -993 | 27.8% |  | -915 | 17.7% |  | -841 | 8.2% |  | -770 | -0.9% |  | -702 | -9.7% |
| - myocardial infarction | -4 |  | -6 | 28.9% |  | -5 | 18.3% |  | -5 | 8.3% |  | -4 | -1.3% |  | -4 | -10.6% |
| - stroke | -773 |  | -988 | 27.8% |  | -910 | 17.7% |  | -836 | 8.2% |  | -766 | -0.9% |  | -698 | -9.7% |
| ΔPatient Worktime Loss | -362 |  | -460 | 27.2% |  | -425 | 17.4% |  | -392 | 8.2% |  | -360 | -0.7% |  | -329 | -9.2% |
| - myocardial infarction | -2 |  | -2 | 28.1% |  | -2 | 17.9% |  | -2 | 8.2% |  | -2 | -1.0% |  | -2 | -10.0% |
| - stroke | -360 |  | -458 | 27.2% |  | -423 | 17.4% |  | -390 | 8.2% |  | -358 | -0.7% |  | -327 | -9.2% |
| ΔCaregiver Time Loss | -1,587 |  | -2,042 | 28.7% |  | -1,876 | 18.2% |  | -1,718 | 8.2% |  | -1,567 | -1.3% |  | -1,421 | -10.5% |
| - stroke | -1,587 |  | -2,042 | 28.7% |  | -1,876 | 18.2% |  | -1,718 | 8.2% |  | -1,567 | -1.3% |  | -1,421 | -10.5% |
| - work time | -643 |  | -819 | 27.4% |  | -756 | 17.6% |  | -696 | 8.2% |  | -638 | -0.8% |  | -582 | -9.4% |
| - leisure time | -944 |  | -1,223 | 29.5% |  | -1,120 | 18.6% |  | -1,022 | 8.3% |  | -929 | -1.6% |  | -839 | -11.2% |
|  | mn USD |  | mn USD | %-change |  | mn USD | %-change |  | mn USD | %-change |  | mn USD | %-change |  | mn USD | %-change |
| Δreal Health Expenses |  |  |  |  |  |  |  |  |  |  |  |  |  |  |  |  |
| - formal hospital | -310 |  | -333 | 7.2% |  | -323 | 4.3% |  | -315 | 1.6% |  | -308 | -0.8% |  | -301 | -3.0% |
| - myocardial infarction | -293 |  | -314 | 7.4% |  | -306 | 4.4% |  | -298 | 1.7% |  | -290 | -0.8% |  | -284 | -3.1% |
| - stroke | -17 |  | -18 | 3.5% |  | -18 | 1.9% |  | -18 | 0.5% |  | -17 | -0.7% |  | -17 | -1.7% |
| Note: Own calculations. | | | | | | | | | | | | | | | | |

| **Table C.4.3.4. Sensitivity to palm oil income elasticity of demand (50% reduction in palm oil consumption): Cumulative impact indicators for 2016-35 (demographic, environment)** | | | | | | | | | | | | | | | | |
| --- | --- | --- | --- | --- | --- | --- | --- | --- | --- | --- | --- | --- | --- | --- | --- | --- |
|  | Policy Scenario  (elas ≈ 1.12-1.14) |  | Sensitivity Scenario 1.  (elas = 0.9) | |  | Sensitivity Scenario 2.  (elas = 1.0) | |  | Sensitivity Scenario 3.  (elas = 1.1) | |  | Sensitivity Scenario 4.  (elas = 1.2) | |  | Sensitivity Scenario 5.  (elas = 1.3) | |
| ***Demographic (cumulative indicators)*** | | | | | | | | | | | | | | | | |
|  | pers-yrs |  | pers-yrs | %-change |  | pers-yrs | %-change |  | pers-yrs | %-change |  | pers-yrs | %-change |  | pers-yrs | %-change |
| Δpopulation | 13,621 |  | 17,468 | 28.2% |  | 16,137 | 18.5% |  | 14,862 | 9.1% |  | 13,632 | 0.1% |  | 12,441 | -8.7% |
| - Bangkok^1^ | 886 |  | 1,389 | 56.7% |  | 1,187 | 33.9% |  | 993 | 12.0% |  | 805 | -9.2% |  | 623 | -29.7% |
| - Central region (exc Bangkok)^1^ | 7,387 |  | 8,706 | 17.9% |  | 8,291 | 12.2% |  | 7,895 | 6.9% |  | 7,515 | 1.7% |  | 7,148 | -3.2% |
| - North region^1^ | 2,544 |  | 3,281 | 29.0% |  | 3,034 | 19.2% |  | 2,796 | 9.9% |  | 2,567 | 0.9% |  | 2,346 | -7.8% |
| - Northeast region^1^ | 2,721 |  | 3,483 | 28.0% |  | 3,219 | 18.3% |  | 2,967 | 9.0% |  | 2,723 | 0.1% |  | 2,487 | -8.6% |
| - South region^1^ | 83 |  | 609 | 631.2% |  | 406 | 387.9% |  | 211 | 153.0% |  | 21 | -74.6% |  | -163 | -295.8% |
| - urban^1^ | 5,019 |  | 7,815 | 55.7% |  | 6,772 | 34.9% |  | 5,768 | 14.9% |  | 4,797 | -4.4% |  | 3,853 | -23.2% |
| - rural^1^ | 8,602 |  | 9,653 | 12.2% |  | 9,365 | 8.9% |  | 9,094 | 5.7% |  | 8,835 | 2.7% |  | 8,587 | -0.2% |
|  | pers-yrs |  | pers-yrs | %-change |  | pers-yrs | %-change |  | pers-yrs | %-change |  | pers-yrs | %-change |  | pers-yrs | %-change |
| Δworkforce | 4,450 |  | 5,655 | 27.1% |  | 5,240 | 17.8% |  | 4,842 | 8.8% |  | 4,459 | 0.2% |  | 4,088 | -8.1% |
| - urban^1^ | 1,621 |  | 2,476 | 52.7% |  | 2,158 | 33.1% |  | 1,852 | 14.2% |  | 1,555 | -4.0% |  | 1,268 | -21.8% |
| - rural^1^ | 2,829 |  | 3,179 | 12.4% |  | 3,082 | 9.0% |  | 2,991 | 5.7% |  | 2,904 | 2.7% |  | 2,821 | -0.3% |
| ***Environment (cumulative indicator)*** | | | | | | | | | | | | | | | | |
|  | Mt CO_2_-eq |  | Mt CO_2_-eq | %-change |  | Mt CO_2_-eq | %-change |  | Mt CO_2_-eq | %-change |  | Mt CO_2_-eq | %-change |  | Mt CO_2_-eq | %-change |
| Δ emissions | 7.52 |  | 7.08 | -5.9% |  | 7.44 | -1.1% |  | 7.76 | 3.2% |  | 8.13 | 8.1% |  | 8.53 | 13.4% |
| Note: Own calculations. | | | | | | | | | | | | | | | | |

| **Table C.5.1. Sensitivity Analysis Scenario: Iso-household consumption** | |
| --- | --- |
| Scenarios | Description |
| Policy Scenario | -50% average household consumption of (palm) cooking oil (flexible household budget) |
| Sensitivity Scenario 1 | -50% average household consumption of (palm) cooking oil  (iso-household budget) |
| Sensitivity Scenario 2 | -50% average household consumption of (palm) cooking oil  (iso-household real consumption) |
| Sensitivity Scenario 3 | -50% average household consumption of (palm) cooking oil  (iso-household & government real consumption) |
| Instrument | sales tax on palm cooking oil |

## C.5. Sensitivity Analyses of iso-household consumption budget restriction

The iso-household consumption budget sensitivity scenario (Table C.5.1) is designed to measure the hypothesized economic inefficiency and overall economic costs to Thai society of introducing a product-specific sales tax on palm cooking oil to achieve our policy goal of reducing energy intakes from palm oil by 50%. As it turns out, our fiscal food policy instrument actually produces economic benefits when final demand impacts are isolated to real investment, indicating that our tax instrument may, both (1) be improving efficiency in a second-best environment, and (2) twisting relative prices in favour of real investment demand and capital accumulation.

Our iso-household consumption sensitivity analyses include three scenarios: (1) an iso-household budget scenario (scenario 1), where household-specific consumption budgets are fixed (relative to the CPI numeraire), (2) an iso-household real consumption scenario (scenario 2), where household-specific real consumption expenditures are fixed (in terms of 2015 base year prices), and (3) a combined iso-household and government real consumption scenario (scenario 3), where both household and government-specific real consumption expenditures are fixed (in terms of 2015 base period prices). Please be reminded that an iso-government consumption budget constraint is imposed on the policy scenario simulations, implying that scenarios 1 and 2 are also subject iso-government consumption constraints (relative to the CPI numeraire).

Scenarios 1-3 can each, individually, be seen as an approach to fixing real private and government consumption in terms of respectively the overall CPI price numeraire and/or individual 2015 base period prices. Our results suggest, however, that conclusions, which can be drawn from each of the three scenarios, are drastically different. While scenarios 1-2 indicate that our fiscal food policy instrument, the palm cooking oil sales tax, have adverse macroeconomic impacts, scenario 3, which isolates real GDP impacts to the production/investment side, indicates that implementation of our tax instrument, and possibly accompanied by compensating government transfers, can lead to positive macroeconomic, nutritional and health outcomes. However, adverse environmental outcomes persist, implying that the implementation of our proposed tax instrument continues to represent a case of policy trade-offs.

The final demand breakdown of macroeconomic outcomes in scenarios 1-3, furthermore, shows, that (1) real exchange rate changes drive trade aggregates (imports/exports increasing in the policy scenario and scenario 3, and declining in scenarios 1-2), and (2) relative investment prices (relative to (other) traded goods prices) drive real consumption and investment expenditures (consumption declining (or unchanged)/investment increasing in the policy scenario and scenario 3; consumption increasing/ investment declining in scenarios 1-2).

In scenario 1, the government introduces compensating transfers to households to compensate for declining private consumption budgets in the policy scenario. However, due to relative price changes, the transfers to maintain counterfactual consumption budgets (relative to the CPI numeraire) overcompensates households leading to a strong USD 18.4bn private consumption expansion, and strong USD -63.4bn crowding-out of real investment (Table C.5.2).

In scenario 2, the government introduces compensating transfers to households to, more specifically, compensate for declining real private consumption expenses in the policy scenario. Inclusion of this compensation policy leads to a relatively moderate USD 2.9bn real GDP contraction. However, the results also indicate that our iso-government consumption budget constraint, due to relative price changes, lead to a USD 9.6bn expansion in real government consumption and USD -12.6bn crowding-out of real investment (Table C.5.2).

| **Table C.5.2. Sensitivity to iso-household consumption (50% reduction in palm oil consumption): Cumulative impact indicators for 2016-35 (economic)** | | | | | | | | | | | |
| --- | --- | --- | --- | --- | --- | --- | --- | --- | --- | --- | --- |
|  | Policy Scenario  (flex hh cons budget) | |  | Sensitivity Scenario 1. (iso-hh cons budget) | |  | Sensitivity Scenario 2. (iso-hh real cons) | |  | Sensitivity Scenario 3. (iso-hh & gov real cons) | |
| ***Real GDP & sales tax & price indices (cumulative and long run indicators)*** | | | |  |  |  |  |  |  |  |  |
|  | mn USD | % of GDP |  | mn USD | % of GDP |  | mn USD | % of GDP |  | mn USD | % of GDP |
| Δreal GDP | 25,145 | 0.227% |  | -34,006 | -0.306% |  | -2,905 | -0.026% |  | 20,954 | 0.189% |
| - health pathway | 95 | 0.001% |  | 156 | 0.001% |  | 149 | 0.001% |  | 35 | 0.000% |
| - sales tax pathway | 25,050 | 0.226% |  | -34,162 | -0.308% |  | -3,054 | -0.028% |  | 20,919 | 0.189% |
|  | mn USD | %-change |  | mn USD | %-change |  | mn USD | %-change |  | mn USD | %-change |
| Δreal GDP (cumulative) | 25,145 | 0.227% |  | -34,006 | -0.306% |  | -2,905 | -0.026% |  | 20,954 | 0.189% |
| - Private Consumption (cumulative) | -9,882 | -0.177% |  | 18,443 | 0.331% |  | 0 | 0.000% |  | 0 | 0.000% |
| - Government Consumption (cumulative) | 8,633 | 0.854% |  | 10,830 | 1.071% |  | 9,670 | 0.957% |  | 0 | 0.000% |
| - Investment (cumulative) | 26,394 | 0.558% |  | -63,279 | -1.338% |  | -12,575 | -0.266% |  | 20,954 | 0.443% |
| - Exports (cumulative) | 35,857 | 0.468% |  | -26,007 | -0.340% |  | 7,800 | 0.102% |  | 37,059 | 0.484% |
| - Imports (cumulative) | 35,857 | 0.455% |  | -26,007 | -0.330% |  | 7,800 | 0.099% |  | 37,059 | 0.470% |
|  | %-points |  |  | %-points |  |  | %-points |  |  | %-points |  |
| Δsales tax (long run, 2035) | 53.5% |  |  | 54.2% |  |  | 53.7% |  |  | 53.7% |  |
| Δinvestment price index (long run, 2035) | -0.8% |  |  | -0.8% |  |  | -0.8% |  |  | -0.8% |  |
| Δreal exchange rate (long run, 2035) | -0.9% |  |  | -0.7% |  |  | -0.8% |  |  | -0.8% |  |
| ***Real Consumption (cumulative indicators)*** |  |  |  |  |  |  |  |  |  |  |  |
|  | mn USD | % of rHC |  | mn USD | % of rHC |  | mn USD | % of rHC |  | mn USD | % of rHC |
| Δreal Household Consumption (rHC) | -9,882 | -0.177% |  | 18,443 | 0.331% |  | 0 | 0.000% |  | 0 | 0.000% |
| - sales tax pathway | -9,930 | -0.178% |  | 18,443 | 0.331% |  | 0 | 0.000% |  | 0 | 0.000% |
| - Bangkok^1^ | -362 | -0.035% |  | 4,958 | 0.479% |  | 0 | 0.000% |  | 0 | 0.000% |
| - Central region (exc Bangkok)^1^ | -3,336 | -0.211% |  | 4,707 | 0.298% |  | 0 | 0.000% |  | 0 | 0.000% |
| - North region^1^ | -935 | -0.120% |  | 1,924 | 0.246% |  | 0 | 0.000% |  | 0 | 0.000% |
| - Northeast region^1^ | -982 | -0.077% |  | 4,477 | 0.349% |  | 0 | 0.000% |  | 0 | 0.000% |
| - South region^1^ | -4,315 | -0.481% |  | 2,378 | 0.265% |  | 0 | 0.000% |  | 0 | 0.000% |
| - health pathway | 48 | 0.0009% |  | 0 | 0.0000% |  | 0 | 0.0000% |  | 0 | 0.0000% |
| - Bangkok^1^ | 8 | 0.0008% |  | 0 | 0.0000% |  | 0 | 0.0000% |  | 0 | 0.0000% |
| - Central region (exc Bangkok)^1^ | 19 | 0.0012% |  | 0 | 0.0000% |  | 0 | 0.0000% |  | 0 | 0.0000% |
| - North region^1^ | 7 | 0.0009% |  | 0 | 0.0000% |  | 0 | 0.0000% |  | 0 | 0.0000% |
| - Northeast region^1^ | 9 | 0.0007% |  | 0 | 0.0000% |  | 0 | 0.0000% |  | 0 | 0.0000% |
| - South region^1^ | 5 | 0.0006% |  | 0 | 0.0000% |  | 0 | 0.0000% |  | 0 | 0.0000% |
| Note: Own calculations. ^1^ Regional consumption %-impacts calculated as share of projected regional totals. | | | | | | | | | | | |

| **Table C.5.3. Sensitivity to iso-household consumption (50% reduction in palm oil consumption): Cumulative impact indicators for 2016-35 (nutrition, biomarker, health)** | | | | | | | | | | | |
| --- | --- | --- | --- | --- | --- | --- | --- | --- | --- | --- | --- |
|  | Policy Scenario  (flex hh cons budget) | |  | Sensitivity Scenario 1.  (iso-hh cons budget) | |  | Sensitivity Scenario 2.  (iso-hh real cons) | |  | Sensitivity Scenario 3.  (iso-hh & gov real cons) | |
| ***Nutrition (long run indicators)*** |  |  |  |  |  |  |  |  |  |  |  |
|  | %-points | % of share |  | %-points | % of share |  | %-points | % of share |  | %-points | % of share |
| ΔSFA energy intake share (long run, 2035) | -0.322% | -3.58% |  | -0.323% | -3.59% |  | -0.322% | -3.59% |  | -0.322% | -3.58% |
| ΔMUFA energy intake share (long run, 2035) | -0.164% | -2.29% |  | -0.157% | -2.18% |  | -0.161% | -2.25% |  | -0.161% | -2.24% |
| ΔPUFA energy intake share (long run, 2035) | 0.298% | 5.80% |  | 0.335% | 6.54% |  | 0.311% | 6.07% |  | 0.312% | 6.09% |
| ***Biomarker (cumulative indicators)*** |  |  |  |  |  |  |  |  |  |  |  |
|  | cum. chg. | % of total |  | cum. chg. | % of total |  | cum. chg. | % of total |  | cum. chg. | % of total |
| ΔTotal-to-HDL cholesterol ratio | -0.101 | -2.16% |  | -0.115 | -2.47% |  | -0.107 | -2.29% |  | -0.112 | -2.39% |
| ***Health (cumulative indicators)*** |  |  |  |  |  |  |  |  |  |  |  |
|  | cases | % of total |  | cases | % of total |  | cases | % of total |  | cases | % of total |
| ΔPatient Incident Cases | -3,570 | -0.095% |  | -4,058 | -0.108% |  | -3,782 | -0.101% |  | -3,921 | -0.105% |
| - myocardial infarction | -2,704 | -0.160% |  | -3,073 | -0.181% |  | -2,864 | -0.169% |  | -2,970 | -0.175% |
| - stroke | -866 | -0.042% |  | -985 | -0.048% |  | -917 | -0.045% |  | -951 | -0.046% |
| ΔPatient premature deaths | -1,861 | -0.098% |  | -2,117 | -0.111% |  | -1,972 | -0.104% |  | -2,043 | -0.107% |
| - myocardial infarction | -1,560 | -0.152% |  | -1,774 | -0.173% |  | -1,653 | -0.161% |  | -1,712 | -0.167% |
| - stroke | -301 | -0.034% |  | -343 | -0.039% |  | -319 | -0.036% |  | -330 | -0.038% |
|  | pers-yrs | % of total |  | pers-yrs | % of total |  | pers-yrs | % of total |  | pers-yrs | % of total |
| ΔPatient Disease Burden (YLD) | -777 | -0.043% |  | -879 | -0.049% |  | -821 | -0.046% |  | -850 | -0.047% |
| - myocardial infarction | -4 | -0.160% |  | -5 | -0.181% |  | -5 | -0.169% |  | -5 | -0.175% |
| - stroke | -773 | -0.043% |  | -874 | -0.049% |  | -816 | -0.046% |  | -845 | -0.047% |
| ΔPatient Worktime Loss | -362 | -0.060% |  | -408 | -0.068% |  | -382 | -0.063% |  | -396 | -0.066% |
| - myocardial infarction | -2 | -0.182% |  | -2 | -0.206% |  | -2 | -0.192% |  | -2 | -0.200% |
| - stroke | -360 | -0.060% |  | -406 | -0.067% |  | -380 | -0.063% |  | -394 | -0.066% |
| ΔCaregiver Time Loss | -1,587 | -0.042% |  | -1,803 | -0.048% |  | -1,681 | -0.045% |  | -1,742 | -0.046% |
| - stroke | -1,587 | -0.042% |  | -1,803 | -0.048% |  | -1,681 | -0.045% |  | -1,742 | -0.046% |
| - work time | -643 | -0.043% |  | -726 | -0.049% |  | -679 | -0.046% |  | -702 | -0.047% |
| - leisure time | -944 | -0.041% |  | -1,077 | -0.047% |  | -1,002 | -0.044% |  | -1,039 | -0.046% |
|  | mn USD | % of GDP |  | mn USD | % of GDP |  | mn USD | % of GDP |  | mn USD | % of GDP |
| ΔHealth Expenses |  |  |  |  |  |  |  |  |  |  |  |
| - formal hospital | -310 | -0.0028% |  | -321 | -0.0029% |  | -314 | -0.0028% |  | -329 | -0.0030% |
| - myocardial infarction | -293 | -0.0026% |  | -303 | -0.0027% |  | -297 | -0.0027% |  | -311 | -0.0028% |
| - stroke | -17 | -0.0002% |  | -18 | -0.0002% |  | -18 | -0.0002% |  | -18 | -0.0002% |
| Note: Own calculations. | | | | | | | | | | | |

| **Table C.5.4. Sensitivity to iso-household consumption (50% reduction in palm oil consumption): Cumulative impact indicators for 2016-35 (demographic, environment)** | | | | | | | | | | | |
| --- | --- | --- | --- | --- | --- | --- | --- | --- | --- | --- | --- |
|  | Policy Scenario  (flex hh cons budget) | |  | Sensitivity Scenario 1.  (iso-hh cons budget) | |  | Sensitivity Scenario 2.  (iso-hh real cons) | |  | Sensitivity Scenario 3.  (iso-hh & gov real cons) | |
| ***Demographic (cumulative indicators)*** |  |  |  |  |  |  |  |  |  |  |  |
|  | pers-yrs | % of total |  | pers-yrs | % of total |  | pers-yrs | % of total |  | pers-yrs | % of total |
| Δpopulation (cummulative) | 13,621 | 0.0010% |  | 15,268 | 0.0011% |  | 14,405 | 0.0011% |  | 15,116 | 0.0011% |
| - Bangkok^1^ | 886 | 0.0005% |  | 1,142 | 0.0007% |  | 1,017 | 0.0006% |  | 1,115 | 0.0007% |
| - Central region (exc Bangkok)^1^ | 7,387 | 0.0018% |  | 8,014 | 0.0020% |  | 7,680 | 0.0019% |  | 7,948 | 0.0019% |
| - North region^1^ | 2,544 | 0.0011% |  | 2,843 | 0.0013% |  | 2,687 | 0.0012% |  | 2,812 | 0.0013% |
| - Northeast region^1^ | 2,721 | 0.0007% |  | 3,051 | 0.0008% |  | 2,891 | 0.0008% |  | 3,034 | 0.0008% |
| - South region^1^ | 83 | 0.0000% |  | 217 | 0.0001% |  | 131 | 0.0001% |  | 207 | 0.0001% |
| - urban^1^ | 5,019 | 0.0007% |  | 6,058 | 0.0008% |  | 5,530 | 0.0007% |  | 5,975 | 0.0008% |
| - rural^1^ | 8,602 | 0.0015% |  | 9,210 | 0.0016% |  | 8,875 | 0.0015% |  | 9,141 | 0.0016% |
|  | pers-yrs | % of total |  | pers-yrs | % of total |  | pers-yrs | % of total |  | pers-yrs | % of total |
| Δworkforce (cumulative) | 4,450 | 0.0007% |  | 4,974 | 0.0007% |  | 4,697 | 0.0007% |  | 4,919 | 0.0007% |
| - urban^1^ | 1,621 | 0.0004% |  | 1,943 | 0.0005% |  | 1,778 | 0.0005% |  | 1,914 | 0.0005% |
| - rural^1^ | 2,829 | 0.0010% |  | 3,031 | 0.0010% |  | 2,918 | 0.0010% |  | 3,005 | 0.0010% |
| ***Environment (cumulative indicator)*** |  |  |  |  |  |  |  |  |  |  |  |
|  | Mt CO_2_-eq |  |  | Mt CO_2_-eq |  |  | Mt CO_2_-eq |  |  | Mt CO_2_-eq |  |
| ΔGHG emissions | 7.52 |  |  | 6.87 |  |  | 7.39 |  |  | 7.62 |  |
| Note: Own calculations. | | | | | | | | | | | |

Turning to our final scenario 3, the government introduces the above mentioned compensating transfers to compensate for declining real private consumption expenses, and restricts real government consumption to be unchanged from the counterfactual. This scenario, which amounts to isolating real GDP impacts to the production/investment side, shows that our tax instrument combined with appropriate government compensating transfers and government real budget restraint leads to a strong USD 20.9bn real GDP expansion. Combined with the observation that real GDP expands continuously from the 2016 impact period onwards (not shown) indicates, that the positive real GDP impacts are due to a combination of (1) improving efficiency in a second-best environment, and (2) increased real investment demand and capital accumulation due to favourable relative price changes.

The Total-to-HDL cholesterol ratio biomarker improves in all three scenarios 1-3 (Table C.5.3), indicating that any of the three configurations of government compensating transfers and budget restraint enhances the biomarker outcomes, with increased biomarker impacts ranging from -0.107 to -0.115 (policy sim=-0.101) or -2.3% to -2.5% (policy sim=-2.2%). The biomarker impacts are also reflected in the nutrition and health impacts with the former varying between –2.2% and +6.6% (policy sim=-2.3% to +5.8%) and the latter varying between -4% and -21% (policy sim=-3% to -18%).

For the key scenario 3, SFA and MUFA energy intake shares change by less than ±0.1% (from policy sim impacts), while the average PUFA energy intake shares expands by +6.1% (policy sim=5.8%), and this improvement of the nutritional profile of Thai diets also causes an increased drop in the cholesterol biomarker -2.5% (policy sim=-2.2%), and clinical health outcomes to improve accordingly: patient incident cases averted jump from +3,570 without compensation to +3,925 averted cases with compensation and government real budget restraint, and premature deaths averted jump from +1,861 to +2,044.

The environmental emissions indicator (Table C.5.4) vary in relation to domestic agricultural land use changes. Since Thai agricultural export shares are relatively small around 4% (not shown), relative demands for agricultural crops are governed by private consumption. Perhaps surprisingly, the overcompensating budget policy in scenario 1, which leads to a strong increase in household consumption, also leads to reduced substitution towards crops with less favourable carbon sequestration characteristics and, hence, to a notable drop in the adverse LUC-related GHG emissions impact to +6.9 Mt CO_2_-eq (policy sim=+7.5 Mt). The two remaining scenarios 2-3 result in unchanged private consumption impacts, and emission impacts vary relatively little between 7.4-7.6 Mt CO_2_-eq (policy sim=7.5 Mt).

In line with nutritional and health indicators, demographic (Table C.5.4) indicators generally improve. The number of population life years gained increases to 14,405-15,268 (policy sim=13,621) with 15,134 life years gained in scenario 3. Furthermore, looking at the geographic population indicators, rural life years gained increases to 8,875-9,210 (policy sim=8,602; scenario 3=9,148) while urban life years gained increases to 5,530-6,058 (policy sim=5,019; scenario 3=5,987). While compensation improves demographic indicators more in urban compared to rural areas, our fiscal food sales tax (with compensation) still has larger progressive health and demographic impacts on rural areas both in absolute and relative terms.

Overall, our iso-household consumption sensitivity scenarios have demonstrated that our fiscal food policy instrument, if accompanied by appropriately specified government compensation transfers and budget restraint, can produce positive outcomes for both macroeconomic, dietary, health and demographic indicators. However, while certain government compensating transfer patterns were shown to reduce environmental impacts, all scenarios continue to produce adverse environmental outcomes.

At the general level, our scenario with isolation of real GDP impacts to the production/investment side (scenario 3) surprisingly produced sizable positive tax-related economic efficiency gains amounting to 0.19% of future real GDP (Table C.5.2). Our analysis showed that this is due to a combination of (1) improved efficiency in a second-best environment, and (2) increased real investment demand and capital accumulation due to favourable relative price changes. In sum, our sensitivity results emphasize that Thai palm oil demand is fairly responsive to our fiscal food sales tax, and, in addition, that efficiency gains are sizable in both absolute and relative terms.

We conclude that our tax instrument, combined with appropriate government compensating transfers and government real budget restraint, can both be effective in achieving nutritional and health targets and, at the same time, bring positive real GDP and (future) welfare outcomes for the Thai population. Nonetheless, we also conclude that, unless the government undertake complementary reductions in their budget (relative to the CPI numeraire), household welfare will be declining. Furthermore, adverse environmental outcomes persist, implying that, regardless of government action and the sign of welfare outcomes, the implementation of our proposed tax instrument continues to represent a case of policy trade-offs between nutritional and health benefits on the one hand, and environmental harms on the other.

# References (Appendices)

Lewington S, Whitlock G, Clarke R, Sherliker P, Emberson J, Halsey J, Qizilbash N, Peto R, Collins R. 2007. Blood cholesterol and vascular mortality by age, sex, and blood pressure: a meta-analysis of individual data from 61 prospective studies with 55 000 vascular deaths. Lancet 370(9604):1829-39.

Lim SS, Gaziano TA, Gakidou E, Reddy KS, Farzadfar F, Lozano R, Rodgers A. 2007. Prevention of cardiovascular disease in high-risk individuals in low-income and middle-income countries: health effects and costs. Lancet 370(9604):2054-62.

Lozano et al. 2012. Global and regional mortality from 235 causes of death for 20 age groups in 1990 and 2010: a systematic analysis for the Global Burden of Disease Study 2010. Lancet 380(9859):2095-2128.

Mensink RP, Zock PL, Kester ADM, Katan MB. 2003. Effects of dietary fatty acids and carbohydrates on the ratio of serum total to HDL cholesterol and on serum lipids and apolipoproteins: a meta-analysis of 60 controlled trials. American Journal of Clinical Nutrition 77(5):1146-55.

NESDB. 2013a. Population projection for Thailand 2010-2040. Office of the National Economic and Social Development Board. Bangkok.

NESDB. 2013b. Population projections at regional and municipality level 2010-2035. Electronic data. Office of the National Economic and Social Development Board. Bangkok.

NHESO. 2009. Thailand National Health and Examination Survey 2008-2009. National Health Examination Survey Office. Health Systems Research Institute. Thailand.

Prentice RL, Kalbfleisch JD, Peterson Jr JD, Flournoy N, Farewelland VT, Breslow NE. 1978. The Analysis of Failure Times in the Presence of Competing Risks. Biometrics 34(4):541-54

Riewpaiboon A, Riewpaiboon W, Ponssongnern K, van den Berg B. 2009. Economic valuation of informal care in Asia: A case study of care for disabled stroke survivors in Thailand. Social Science & Medicine 69(4):648-53.

UN. 2015. Electronic data. World Population Prospects, the 2015 Revision. United Nations. URL: <http://esa.un.org/unpd/wpp/index.htm>. (accessed 14. September 2015)

WHO. 2013. "WHO methods and data sources for global burden of disease estimates 2001-2011". Global Health Estimates Technical Paper WHO/HIS/HSI/GHE/2013.4. WHO: Geneva.

WHO. 2014. World Health Organization Mortality Database. World Health Organization. Geneva. URL: <http://www.who.int/healthinfo/mortality_data/en/>

1. A few initial Total-to-HDL ratio biomarker outliers were outside the chosen range and eliminated from the database. [↑](#footnote-ref-1)
2. Based on the simulated lookup tables, the clinical health outcome module was developed to encompass both a set of discontinuous spline-functions and a set of fitted 10th degree polynomials – all gender, rural/urban, and age group-specific. [↑](#footnote-ref-2)
3. Lower and upper limits on age-, gender-, and household-specific transition probabilities were imposed to limit annual transition probabilities between 5 year age groups to the interval [0.15;0.25]. For age-, gender, household-, and time-specific transition probabilities outside the specified interval, imposition of transition probability limits were ensured by a final ex-post dynamic calibration procedure where calibration of transition probabilities was replaced by calibration of net immigration rates. [↑](#footnote-ref-3)
4. Caregiver time losses should, in principle, be subject to the same mechanism. Our model specification, which specifies constant caregiver worktime losses per incident case, does not, however, allow for modelling of the age distribution of caregivers, and, hence, does not allow for modelling of r. age variation impacts on caregiver time losses. This represents a (minor) limitation of our current model specification, but it only affects the results of the sensitivity analyses – not the results of the baseline analysis. [↑](#footnote-ref-4)
